# Ribosomal architecture and rRNA modification landscape in the tick-borne parasite *Babesia divergens*

**DOI:** 10.1038/s41467-026-75282-4

**Published:** 2026-08-06

**Authors:** Cristina Gutierrez-Vargas, Lee S. Izhaki-Tavor, Diana G. Calvopina-Chavez, Caroline D. Keroack, Pablo Copello, Manoj T. Duraisingh, Mélissa Léger-Abraham

**Affiliations:** 1 Department of Microbiology, Harvard Medical School | Blavatnik Institute, Boston, MA USA; 2 Department of Immunology and Infectious Diseases, Harvard T. H. Chan School of Public Health, Boston, MA USA; 3 Division of Molecular Medicine, Boston Children’s Hospital, Boston, MA USA; 4 Present Address: Department of Internal Medicine, University of Utah, Salt Lake City, UT USA; 5 Present Address: Department of Molecular Microbiology and Immunology, Brown University, Providence, RI USA; 6 Present Address: École Supérieure de Chimie Physique Électronique de Lyon, Villeurbanne, France

**Keywords:** Parasitic infection, Electron microscopy, Ribosome, RNA modification

## Abstract

*Babesia* are tick-borne intracellular apicomplexan parasites that infect a wide range of wild and domestic animals (e.g., cattle), resulting in significant economic losses to the livestock industry. Humans are considered accidental hosts for a few *Babesia* species. *Babesia microti* and *B. divergens* are the most prevalent causes of human babesiosis that are showing a broadening geographic distribution. Due to the complex life cycle of *Babesia* species, their survival depends on the precise control of gene expression, which is primarily regulated by epigenetic, transcriptional, and post-transcriptional mechanisms. High-resolution structural information on key components of the translation machinery, such as ribosomes, could aid in the development of antiparasitic drugs. Here, we report cryogenic electron microscopy ribosome structures from *B. divergens*, showing associated tRNAs, an mRNA fragment, and RACK1, a signaling scaffold crucial to translation regulation. Density map analysis displays ribosome regions at high resolution, which, when combined with nanopore sequencing, enabled the comprehensive identification of rRNA modifications. The rRNA modifications localize not only to the reduced *B. divergens* rRNA expansion segments but also to functionally essential ribosomal sites.

## Abstract (web summary)

*Babesia microti* and *B. divergens* are tick-borne parasites that are the most prevalent causes of human babesiosis. Here, the authors present cryo-EM structures of ribosomes from *B. divergens*. Nanopore sequencing reveals distinct rRNA modifications.

## Introduction

*Babesia* species are tick-borne parasites that infect a broad spectrum of wild and domestic animals worldwide (e.g., cattle), resulting in significant economic losses to the livestock industry^1,2^. At least nine *Babesia* species (*B. divergens, B. duncani, B. crassa-*like, *B. microti, B. venatorum*, *B. odocoilei, B. bovis*, *B. hegotelforum*, and *B. bigemina)* are known to be pathogenic to humans, who are considered accidental hosts^3^. *Babesia microti* (in the United States) and *B. divergens* (in Europe) are the most prevalent species that cause human babesiosis^3–9^. In humans, *Babesia* parasites infect erythrocytes, causing malaria-like symptoms^10,11^. Treatment typically involves a combination of antibiotics and antimalarials, including azithromycin with atovaquone or clindamycin with quinine^8^. Human babesiosis can also be fatal, especially in immunocompromised individuals, in whom prolonged treatment, sometimes extending over two years, is often required to clear the infection^12–14^. Drug pressure increases the risk of resistance mutations, potentially leading to chronic infection or relapsing disease^15^. The development of new therapeutics targeting *Babesia*-specific molecular pathways, rather than relying on repurposed drugs (e.g., a combination of antibiotics and antimalarials), is urgently needed. This need is further underscored by the expanding geographic distribution of babesiosis in northern regions, particularly in the mid-Atlantic region of the USA^16,17^.

Apicomplexans, including *Toxoplasma*, *Plasmodium*, and *Babesia*, are specialized parasitic protozoans that utilize an apical complex to invade host cells. Their life cycle typically involves alternating between sexual reproduction in a definitive host and asexual reproduction within an intermediate host. Notably, *Babesia spp*. that are pathogenic to humans and that belong to the *sensu stricto* phylogenetic clade (e.g., *B. divergens*, *B. crassa-like*, *B. venatorum*, *B. odocoilei*, *B. bovis*, *B. hegotelforum*, and *B. bigemina*) distinguish themselves by their ability to survive tick molting (Ixodidae) and to pass from mother to offspring (transovarial transmission), allowing it to persist without a vertebrate host and therefore, establish reservoirs^18–20^. These survival strategies are maintained through gene regulation at the epigenetic, transcriptional, and post-transcriptional levels^21,22^. As they transition between vectors and mammalian hosts and progress through the various stages of their life cycle, translation regulation becomes crucial so that parasites can quickly adapt their protein expression level accordingly. Nonetheless, the mechanisms underlying protein synthesis in apicomplexan parasites remain understudied compared to those in bacteria, yeast, or humans.

Protozoan ribosomes present unique regulation opportunities, as they exhibit heterogeneity and plasticity in their ribosomal protein composition, recruitment of accessory proteins, and the types of ribosomal RNA (rRNA) incorporated^23^. Ribosomal RNA modifications are also of particular interest because they have been linked to altered translation landscapes with biological relevance^24–26^; for example, they differ in patterns between healthy and tumor cells^27–29^, or alter antibiotic binding in bacteria as a mechanism of antibiotic resistance^30^. Although only a few rRNA modifications have been reported in a recent cryogenic electron microscopy (cryo-EM) ribosome structure from *P. falciparum*^31^, no comprehensive characterization of rRNA modifications has been conducted concomitantly with ribosome structural analysis from any apicomplexans^31–36^, limiting our understanding of the mechanisms and potential roles of individual modified nucleotides in these parasites.

In this work, we report an atomic model of the cytoplasmic ribosome from the tick-borne apicomplexan pathogen, *B. divergens*, at a global resolution of 2.7 Å, providing a view of both the ribosomal protein and rRNA architecture. The model shows bound transfer RNAs (tRNAs) associated with a messenger RNA (mRNA) fragment and the regulatory scaffold protein RACK1, which is integral to translation control. Using high-resolution cryo-EM maps in combination with nanopore sequencing, we thoroughly characterize rRNA modifications. These modifications are present not only within the minimized rRNA expansion segments of *B. divergens* but also at key functional sites of the ribosome, highlighting additional avenues for therapeutic intervention against babesiosis.

## Results

### Overview of the *Babesia* ribosome high-resolution structure

**Fig. 1:**
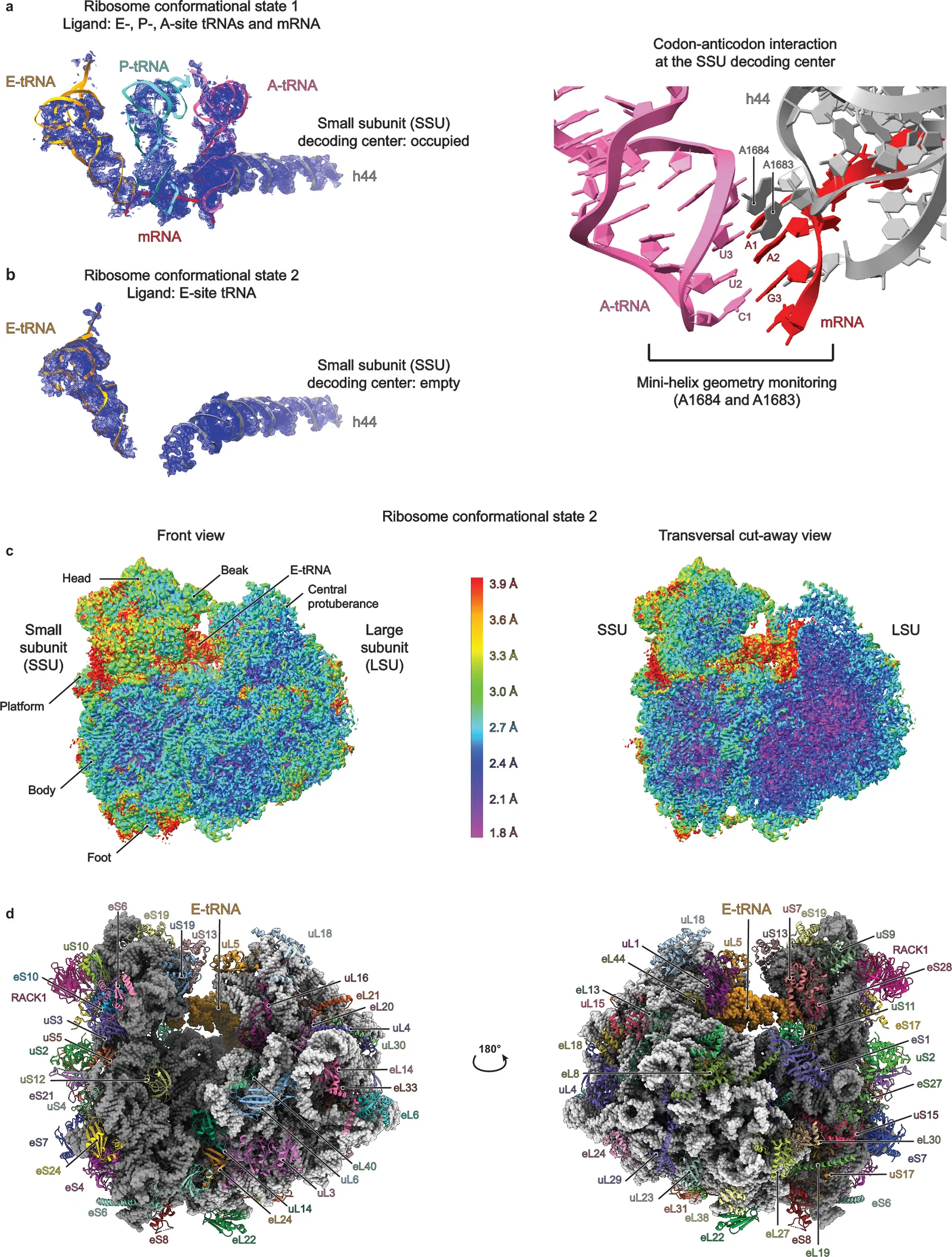
Overview of *B. divergens* ribosome structures. **a** Left panel: Partial density from the consensus maps corresponding to the three tRNAs (E-, P-, and A-site tRNAs) and an mRNA fragment bound to the ribosome (Conformational state 1, 3D Class 1), see Supplementary Figs. 1, 2, and Supplementary Table 1). Right panel: Enlarged view of the decoding center. A1683 and A1684 are in a flipped-out conformation relative to helix 44 (h44) of the small subunit (SSU) and monitor the geometry of the first two base-pairs of the codon-anticodon mini-helix. **b** As described in a, but for the conformational state 2 (3D Class 3), where a single E-tRNA is bound to the ribosome, and that is further described in c and d (see Supplementary Figs. 1, 2, and Supplementary Table 2). **c** Left panel: Sharpened multi-body refinement maps of the *Babesia* ribosome bound to an E-site tRNA. The combined individual bodies are colored according to the estimated local resolution (color code in the middle). The morphological features of both subunits are labeled. LSU: large subunit. Right panel: Transversal cut-away view showing the highest resolution (1.8 Å) within the ribosome core. **d** Left panel: Ribosome model for the corresponding cryo-EM maps shown in c. The ribosomal proteins are shown in ribbon-colored representations. The rRNA is represented in dark and light gray spheres for the SSU and the LSU, respectively, and the E-site tRNA is in orange. Right panel: 180-degree rotation along the Y axis.

**Fig. 2:**
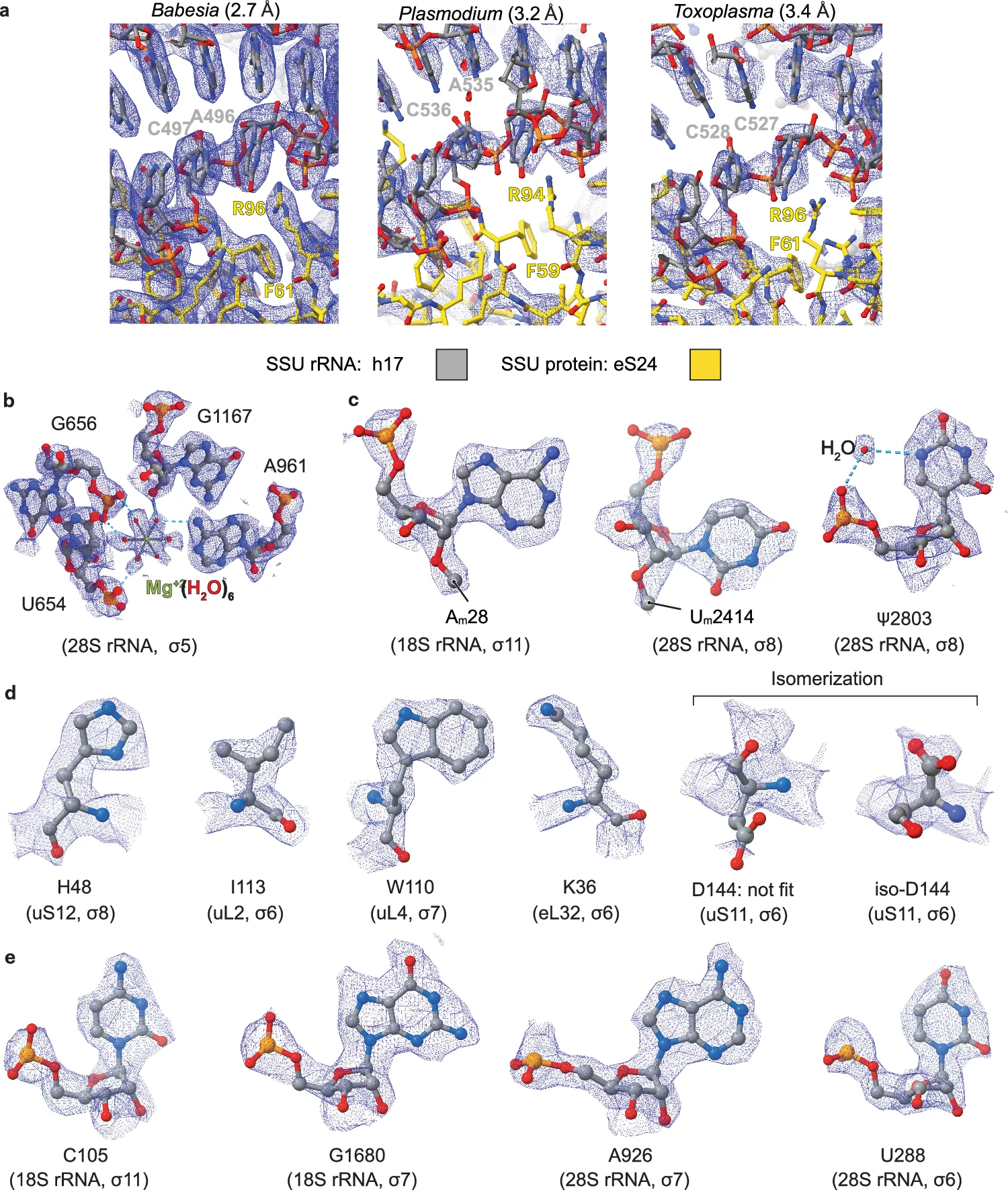
High-resolution details of the *B. divergens* ribosome bound to an E-site tRNA. **a** Global views of the SSU estimated with RELION (FSC = 0.143) centered on the body (h17: C497 and A496 in gray, and eS24: R96 and F61 in yellow), illustrating the quality of the cryo-EM maps presented in this study when compared to those of *P. falciparum* (EMD-2660, PDB 3J7A, contour level of 0.2) and *T. gondii* (EMD-6780, PDB 5XXU, threshold level of 0.06). **b** Atomic view of the interaction between the H25.1 loop from the LSU with a magnesium ion (green). Gray dashed lines correspond to the first-shell interactions between water molecules (red spheres) and the magnesium ion, while cyan dashed lines indicate hydrogen bonds formed between water molecules and rRNA nucleotides. **c** An atomic view of a few rRNA modifications observed throughout the ribosome structure identified by cryo-EM density analysis, such as methylations (indicated by a line) and pseudouridylation (coordinated by a water molecule). Nanopore sequencing experiments confirmed the methylation and pseudouridylation of A28, U2414, and U2803, respectively (see “Methods”). **d** An atomic view of residues from the SSU and LSU, where the side chains are clearly defined for different residue types, including the fitting of Asp144 isomerization from uS11. **e** Examples of nucleotides from the 18S (SSU) and 28S (LSU) rRNA, where the ring structures of the bases and riboses are well resolved.

Among the *Babesia sp*. pathogenic to humans, *B. microti* and *B. divergens* are the most prevalent. The ease and readily available continuous in vitro culture propagation of *B. divergens*, compared to *B. microti*, made it the ideal organism for ribosome structure determination. We isolated ribosomes from an asynchronously grown *B. divergens* Rouen 1987 strain. Following ribosome purification, cryo-EM sample preparation, and image processing, including particle sorting, we obtained two main conformational states with the typical and distinctive morphology of ribosomes, in which both subunits are present (Supplementary Figs. 1–3, see “Methods”). In one conformational state (3D Class 1), the ribosome particles contain three tRNAs occupying the E-, P-, and A-sites, and an mRNA fragment (approximately nine nucleotides) encompassing the tRNA binding sites (Fig. 1a, left panel and Supplementary Table 1). Consistent with this conformational state, the two adenosines within the small subunit helix 44 (h44), responsible for monitoring the first two base-pairs of the codon-anticodon mini helix (A1683 and A1684, corresponding to human A1824 and A1825^37,38^ are in a flipped-out conformation, stabilizing and favoring the accommodation of the A-site tRNA in the ribosome during the tRNA selection step (Fig. 1a, right panel). In the second conformational state (3D Class 3), the ribosome particles contain an E-site tRNA (Fig. 1b and Supplementary Table 2), where A1683 remains stacked within h44 (Supplementary Fig. 4). The corresponding density for A1684 is unresolved, consistent with increased flexibility when the A-site is unoccupied. We used local contrast transfer function (CTF) refinement, 3D re-refinement, and post-processing in RELION for both conformational states, followed by masking and multibody refinement. For the three tRNAs-bound ribosome conformational state, we obtained final 80S maps with a global resolution of 3.1 Å. For the single E-site tRNA-bound ribosome, the global resolution is 2.7 Å. Although we built atomic models for both conformational states (ribosomes with three tRNAs and mRNA versus ribosomes with a single E-site tRNA), all our analysis originates from ribosomes bound to the single E-site tRNA, due to its higher resolution, with areas within the ribosome core reaching high resolution (local resolution approximation of 1.8 Å, Fig. 1c). To build the ribosome protein component of the atomic model (see “Methods”), we initially used a blast search originating from *P. falciparum* 3D7 (PDB IDs: 3J7A, 3J79)^32^ to *B. divergens* 1802A strain (Supplementary Tables 3 and 4). The identified sequences were then implemented in Alphafold2^39^ to obtain structure prediction, which were then docked into the *B. divergens* maps using ChimeraX^40^. We also analyzed and confirmed the complement of ribosomal proteins from purified ribosome samples by liquid chromatography-tandem mass spectrometry (LC/MS-MS) (Supplementary Tables 5–7). For the rRNA component, we used sequences retrieved from RNAcentral^41^, generated sequence alignment between *B. divergens* and *T. gondii* using Clustal Omega^42^, and obtained secondary structure prediction maps using RNAcentral. We selected conserved fragments to initiate the modeling of the rRNA. Iterative rounds of manual model adjustment (Coot)^43^ and model refinement (PHENIX)^44^ led to the final atomic model of the *B. divergens* cryo-EM ribosome structure (Fig. 1d, Supplementary Table 2). The *B. divergens* atomic model has a higher resolution than any other apicomplexan ribosome structure previously determined^31–36^ (Fig. 2), as reflected in the better-defined cryo-EM maps, especially in the small subunit (Fig. 2a). Our structure includes water molecules and ions (Zn^+2^ and Mg^+2^, Fig. 2b), which were assigned based on their typical chemical coordination environments (e.g., tetrahedral vs octahedral)^45^, coupled with the strength and quality of the density maps within specific areas. We also observed post-transcriptional modifications (see below), such as rRNA modifications (Fig. 2c), as well as post-translational modifications, including residue isomerization (Fig. 2d), which was also recently described in human ribosomes^45^.

### Features of the *Babesia* ribosomal proteins

We modeled more than 94% of the ribosomal protein within the *B. divergens* ribosome atomic model, identifying 33 and 46 ribosomal proteins for the small and large subunits (SSU and LSU), respectively (Supplementary Tables 3 and 4). We found that the cryo-EM density maps could not accommodate several sequence regions from *B. divergens* uS7 (residues 70–97), eS10 (residues 26–46), uS12 (residues 64–106) (Supplementary Fig. 5), eL18 (residues 122–146) and eL24 (residues 61–72) (Supplementary Fig. 6), as previously annotated in the genome of the *B. divergens* 1802A strain. After reexamining the corresponding RNA-seq data for each of these proteins, which likewise lacked these sequences, we conclude that the genomic regions are, in fact, misannotated as introns^46^.

We did not identify an eS17 protein sequence in *B. divergens* 1802A using the *P. falciparum* sequence (PF3D7_1242700) in a blast search, but did identify a corresponding sequence in *B. bovis* (BBOV_III001250). Genomic analysis using *B. bovis*’s sequence led us to identify a corresponding protein sequence in *B. divergens* Rouen 1987 strain at the CCSG02000001.1: 1890202.1890772 supercontig, for which there is a genome sequence gap in the *B. divergens* 1802A reference strain. The eS17 *B. divergens* sequence differs from the *B. bovis* sequence at four positions, which were modeled accordingly in the atomic model (*B. bovis* numbering: R3G, A22G, S25G, and Q40L) (Supplementary Fig. 7a). A similar strategy, using *T. gondii* as a reference sequence (TGME49_213580), led us to identify the unannotated genomic sequence corresponding to eL41 in the *B. divergens* 1802A reference strain (JAHBMH010000034: 30674.30988 supercontig). The protein sequence matched the density maps, including the N-terminal sequence of eL41 (residues 1–14), which is absent in humans but present in *P. falciparum*, *T. gondii*, and in *B. divergens* (Supplementary Fig. 7b). We did not model the ribosomal proteins forming the P stalk (uL10, uL11, P1, P2) because their corresponding densities were too weak, despite their presence in our sample as determined by mass spectrometry analysis (Supplementary Tables 5-7). As for the L1 stalk, formed by the ribosomal protein uL1, we were able to dock uL1 into a low-pass-filtered map at 6 Å^47^. However, only partial density remained visible in the composite maps (Supplementary Fig. 8). Both stalks are known to be flexible, with the L1 stalk binding the deacylated-tRNA during translocation and regulating its release, while the P stalk helps recruit and stimulate the ribosome-associated GTPase translation factors^48–51^.

### Features of *Babesia* RACK1

**Fig. 3:**
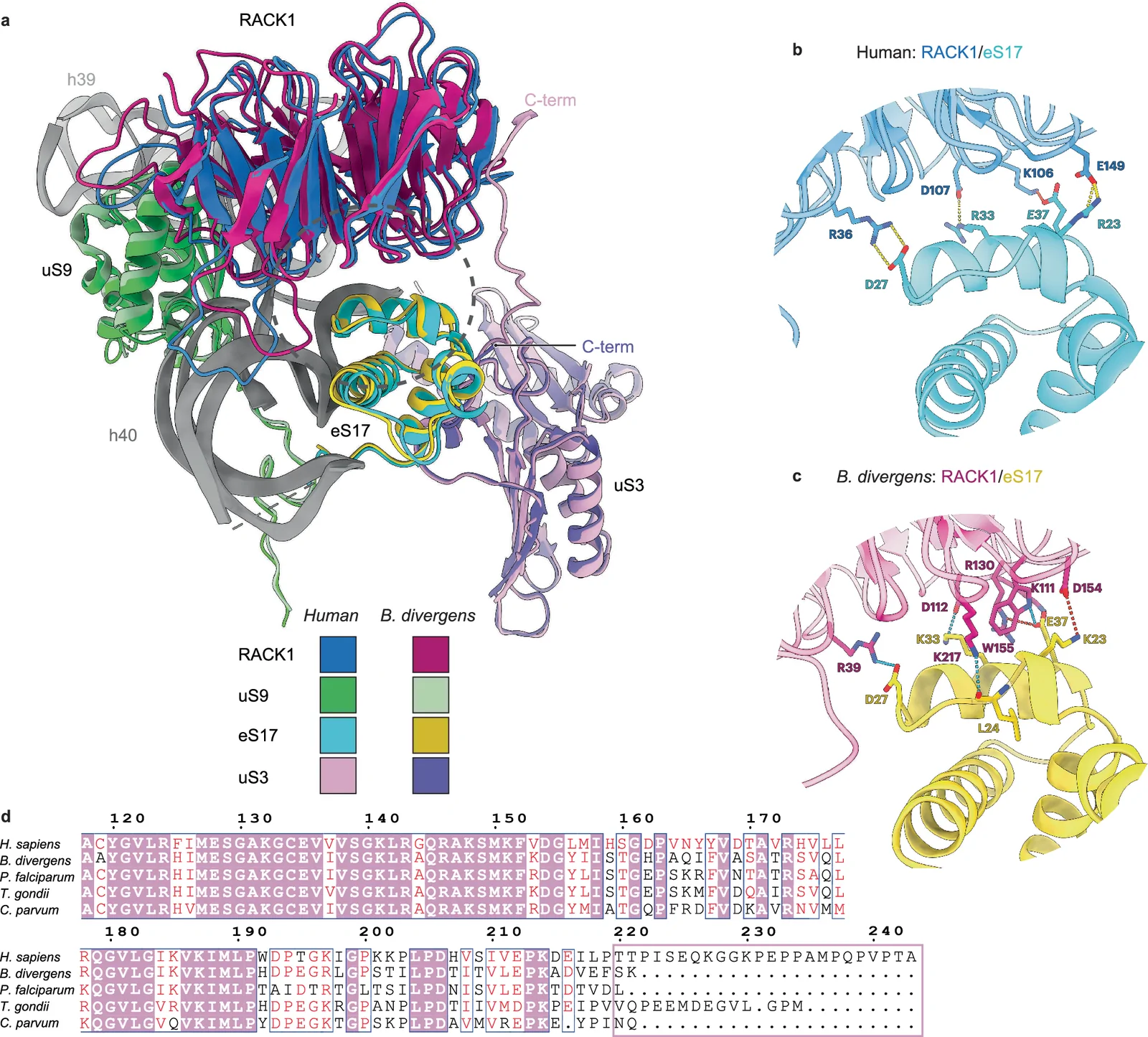
Molecular details of the interaction between RACK1 and eS17. **a** An overview of the localization of RACK1 within the head of the SSU, where it is surrounded by uS3, uS9, eS17, and helices 39 and 40 (see also Fig. 1). Human (PDB 8QOI) and *B. divergens* proteins are colored and labeled accordingly, with their respective C-terminus indicated (C-term). A dotted gray circle corresponds to the zoomed region shown in panels **b** and **c**. **b** Key interactions between RACK1 and eS17 from humans. The hydrogen bonds and salt bridges are displayed with dotted yellow and red lines, respectively. **c** Key interactions between RACK1 and eS17 from *B. divergens*. The hydrogen bonds and salt bridges are displayed with dotted cyan and red lines, respectively. **d** Partial sequence alignment of uS3 showing the sequence divergence at the C-terminus of uS3 (pink rectangle). Sequence alignment was generated using Clustal Omega and displayed using ESPript3. White letters on a pink background correspond to identical residues, while red letters on a white background indicate conservation. The numbering is based on the human sequence. VEuPathDB gene IDs: *H. sapiens*: P23396, *B. divergens*: Bdiv_011570, *P. falciparum*: PF3D7_1465900, *T. gond*ii: TGME49_232300, *C. parvum*: cgd7_2250.

In addition to the ribosomal protein complement, the eukaryotic ribosome binds to the receptor for activated C kinase 1 (RACK1)^52–54^, a scaffolding and signaling protein^55^ that increases the translation efficiency of capped mRNA and contributes to heterogeneity and diversification within the ribosome population^23^. RACK1 has been implicated in various diseases, including cancer progression, inflammation, and neurological disorders^54^. RACK1 interacts with the SSU head domain near the mRNA exit tunnel (Figs. 1d, 3, and Supplementary Fig. 9)^53^. In the human ribosome, the C-terminal tail of uS3 mediates contact with the propeller blades 4 and 5 of RACK1 (Supplementary Fig. 10)^37^. At the same time, RACK1 interacts mainly with eS17 and uS9, and with h39 and h40 through a network of electrostatic interactions (Arg/Lys patch, Fig. 3 and Supplementary Fig. 9)^38,45,52^. In previous single-particle cryo-EM ribosome structures from *Plasmodium* and *Toxoplasma*, density for RACK1 was either not observed or very weak, and unmodeled^32,34,35^. However, using homology modeling and cellular and molecular biology studies, RACK1 from *P. falciparum* was found to associate with ribosomes from polysome fractions^56^. Moreover, in situ structures of ribosomes from cryo-FIB milled *P. falciparum*-infected human erythrocytes across all asexual stages show the presence of RACK1 on the SSU, although the lower resolution limited the detailed characterization of the interaction^33^. In apicomplexans, including *Babesia*, the C-terminal tail of uS3 is significantly shorter than in humans (Fig. 3d)^37^. It was thus suggested that this loss of contacts would substantially destabilize the RACK1/ribosome interactions in apicomplexans. The *B. divergens* ribosome structure presented in this study includes RACK1, for which the apparent density corresponds to a high level of occupancy on the ribosome. Our atomic model lacks the last ten residues from the C-terminus of uS3, but based on the human ribosome structure comprising RACK1^37^, four additional residues would be required to reach the propeller blades of RACK1. Therefore, even if uS3 were fully modeled, uS3 would not reach RACK1. Although the interactions between RACK1 and eS17 are of the same nature in human ribosomes and in *B. divergens* ribosomes (hydrogen bonds, salt bridges, and hydrophobic contacts), the binding interface is slightly larger in *B. divergens*, where five eS17 residues interact with RACK1 compared to four in humans. The molecular details of the interaction between RACK1 and the SSU in *B. divergens* are consistent with a recent study describing the interaction between RACK1 and the ribosomes from *P. falciparum* when treated with artemisinin^31^.

### Features of the *Babesia* rRNA

The secondary structure of rRNA is highly conserved across all domains of life. Eukaryotes have acquired multi-nucleotide insertions, termed expansion segments (ESs), that emerge from the universal rRNA core relative to prokaryotes. These ESs are mostly located on the ribosome’s surface, distant from the conserved functional centers, and exhibit remarkable variability in both sequence and length across species. While the functions of these ESs remain poorly understood, several studies have reported implications in ribosome biogenesis^57–59^, translation regulation^60^, and in the fidelity and accuracy of protein synthesis^61,62^. We thus compared human ESs^38^ with those of *B. divergens* to identify differences that could further our understanding of their roles across evolution.

The human SSU ESs are all found in *B. divergens* (Table 1). While most are conserved, with less than 20% sequence length variation, a few are truncated (ES3S, ES6S, and ES12S), all located on the solvent-exposed side of the SSU. No function has been attributed to these ESs thus far. The ESs from the LSU exhibit significantly greater variability than those from the SSU. For example, the higher-eukaryote (vertebrate) specific segments (ES30L and ES44L) are absent from *B. divergens*, many ESs are substantially truncated, and several ES-specific helices (ES7Ld-h, ES9La, and ES20La) are missing.

**Table 1:** Conservation degree analysis of *B. divergens* ESs when compared to human ESs

rRNA	Expansion segments		Conservation type
	Humans		*B. divergens*
**18S**	ES2S		S
	ES3S	a, b	T
	ES6S	a	C
		b	T
		c, d, e	C
	ES7S, ES9S, ES10S		C
	ES12S		T
	ES13S		S
	ES14S		C
**5.8S**	ES3L, ES4L		C
**28S**	ES4L, ES5L		C
	ES7L	a, b, c	T
		d, e, f, g, h	A
	ES8L		S
	ES9L	a	A
		H30, H31	S
	ES10L		S
	ES12L, ES13L		C
	ES15L	a	T
	ES19L		T
	ES20L	a	A
		b	C
	ES26L		C
	ES27L	a, b, c	T
	ES30L		A
	ES31L	a	C
		b, c	T
	ES39L	a-b	T
	ES41L		C
	ES43L		S
	ES44L		A
	ES45L		C

C Conserved ES ( < 20%).

S Shorter ES ( > 20% decrease in size but <10 nucleotides).

T Truncated ES ( > 20%).

A Absent.

*Babesia divergens* ESs are generally conserved or truncated compared to *T. gondii* and *P. falciparum*, respectively (Table 2)^32,35^. Despite a closer phylogenetic relationship to *P. falciparum*, *B. divergens* ES lengths are more comparable to those of *T. gondii*. This is likely due to lineage-specific expansion of rRNA in *Plasmodium* species, which contains several species-specific insertions (ES7Lb1, ES34L, ES36L) and unusually long ESs (ES6S, ES7S, ES9S, ES10S). In contrast, the rRNA of *B. divergens* is notably compact, consistent with its reduced genome size^63–65^.

**Table 2:** Conservation degree analysis of *B. divergens* ESs when compared to *T. gondii* and *P. falciparum* ESs

rRNA	Expansion segments		Conservation type	
	*B. divergens*		*T. gondii*	*P. falciparum*
**18S**	ES2S		C	C
	ES3S	a	E	E
		b	C	C
	ES6S	a	C	E
		b	E	E
		c	C	E
		d	C	C
		e	E	E
	ES7S, ES9, ES10		C	E
	ES12S		C	C
	ES13S		L	L
	ES14S		C	C
**5.8S**	ES3L, ES4L		C	S
**28S**	ES4L, ES5L		C	C
	ES7L	a	E	C
		b	C	C
		b1		*Pf-specific*
		c	C	C
	ES8L		C	E
	ES9L	H30	C	E
		H31	C	C
	ES10L		T	T
	ES12L, ES13L		C	C
	ES15L	a	E	E
	ES19L		C	C
	ES20L	b	C	C
	ES26L		C	E
	ES27L	a, b, c	E	E
	ES31L	a	C	C
		b, c	L	E
	ES34L, ES36L			*Pf-specific*
	ES39L	a-b	C	E
	ES41L		C	C
	ES43L		C	L
	ES45L		C	C

C Conserved ES ( < 20%).

S Shorter ES ( > 20% decrease in size but <10 nucleotides).

T Truncated ES ( > 20%).

L Longer ES ( > 20% increase in size but <10 nucleotides).

E Expanded ES ( > 20%).

A Absent.

**Fig. 4:**
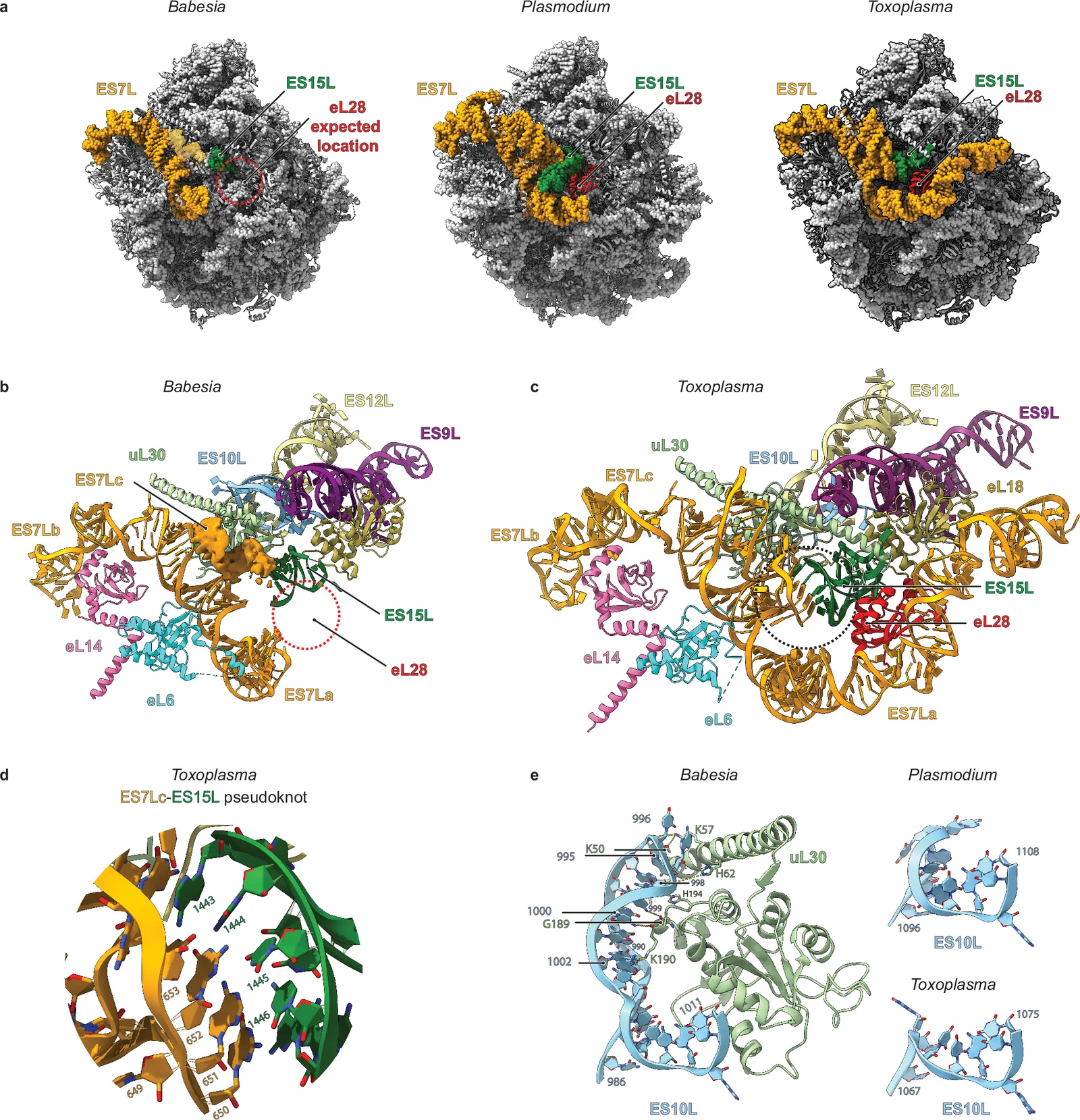
Molecular details of the expansion segments (ES) from the *B. divergens* large subunit (LSU) in comparison to *P. falciparum* and *T. gon*dii. **a** The large ribosomal subunit is shown from the solvent side. General overview of the ESs localization that are mentioned in the text: ES7L and ES15L are labeled and colored, respectively, in orange and green. The ribosomal protein eL28 (red), present in *P. falciparum* and *T. gondii*, is absent from the *B. divergens* ribosome atomic model (indicated by a dashed red circle). **b** Interaction details within the vicinity of ES7L, ES10L and ES15L in *B. divergens*, emphasizing the shorter ES7L and ES15L, the unmodeled ES7Lc, and the absence of eL28. **c** Interaction details within the vicinity of ES7L, eS10L and ES15L in *T. gondii*, highlighting the longer ES7L RNA fragment. **d** ES7Lc and ES15L form a pseudoknot between nucleotides 650-653 and 1443-1446, which is shown as an enlargement of (**c**). **e** Comparison of ES10L between *B. divergens*, *P. falciparum*, and *T. gondii* ES10L. Nucleotides from *B. divergens* ES10L interacting with uL30 residues are indicated, while the shorter ES10L from *P. falciparum* and *T. gondii* do not sustain these interactions.

Much of the structural difference between *B. divergens* and closely related apicomplexans is concentrated in a cluster of expansion segments located at the back of the LSU: ES7L, ES10L, and ES15L (Fig. 4). In eukaryotes, ES7L is of particular interest, as it interacts with numerous ribosomal assembly factors, highlighting its significance during ribosome biogenesis^66^. ES7L is the largest eukaryotic expansion segment and is subdivided into eight helices (ES7La-h). In *B. divergens* and *T. gondii*, ES7L comprises only three helices (ES7La, b, and c), but ES7La is truncated by more than 50% in *B. divergens* relative to *T. gondii* (Fig. 4a and Table 2). The adjacent *B. divergens* ES15L is also markedly truncated when compared to *P. falciparum* and *T. gondii* (Fig. 4a). While ES7Lb interacts with the ribosomal protein eL14, we were unable to model ES7Lc due to blurred density, indicative of its flexibility (Fig. 4b). This is contrasting with the equivalent area in *T. gondii*, where ES15L forms a pseudoknot with ES7Lc, which most likely reduces its flexibility (Fig. 4c, d). Within the same vicinity, we found that eL28 is absent from the *B. divergens* ribosome cryo-EM density (Fig. 4a, c). Although we identified eL28 homologs in other *Babesia* species that are pathogenic to humans, such as in *B. duncani* and *B. microti*, we did not identify eL28 in the genome of *B. divergens* 1802A strains. eL28 was also absent from the corresponding mass spectrometry data (Supplementary Table 6).

Sitting towards the back of ES7Lc, we found that ES10L is expanded in *B. divergens* compared with *P. falciparum* and *T. gondii*, respectively (Fig. 4b, c, and e). While *T. gondii* ES10L is located underneath ES9L and ES12L (Fig. 4c), B*. divergens* ES10L extends along the solvent-exposed surface and predominantly contacts uL30 (Fig. 4b, e). In combination, the rearrangement of ES7L, ES10L, and ES15L results in a significant modification of the LSU surface.

### *Babesia* rRNA modifications

Ribosomal RNA modifications are of interest due to their roles in translation regulation and antibiotic resistance^30^. Yet, they have not been comprehensively characterized alongside ribosome structures in apicomplexans, where only a few were reported in a recent *P. falciparum* cryo-EM ribosome structure^31^. We assigned rRNA modifications based on protruding density that is continuous with the ribose or base in the high-resolution maps of the *B. divergens* ribosome containing a single E-site tRNA (Conformational state 2, 3D Class 3). Our search was also cross-referenced with known regions of the ribosome containing rRNA modifications, as described in human ribosomes (near atomic resolution) and in *Leishmania* parasites, where both studies reported a high number of rRNA modifications^26,45,67^. We complemented our strategy with nanopore sequencing^68,69^, which could enable the identification of rRNA modifications in regions of the ribosome with lower resolution. Also, because we used asynchronous cultures, nanopore sequencing could allow the identification of rRNA modifications that may be present at a specific developmental stage. We hypothesize that if that were the case, these modifications would have a lower occurrence, which would be averaged out in the cryo-EM dataset despite being present within high local resolution (e.g., 2.2 to 2.6 Å), and therefore, would be within the noise level. For this reason, we applied two different rRNA modification occurrence thresholds during nanopore sequencing analysis: modifications identified and present more than 50% (≥50%) and between 30 and 50% (≥30% and < 50%) for a given position (see “Methods”).

**Fig. 5:**
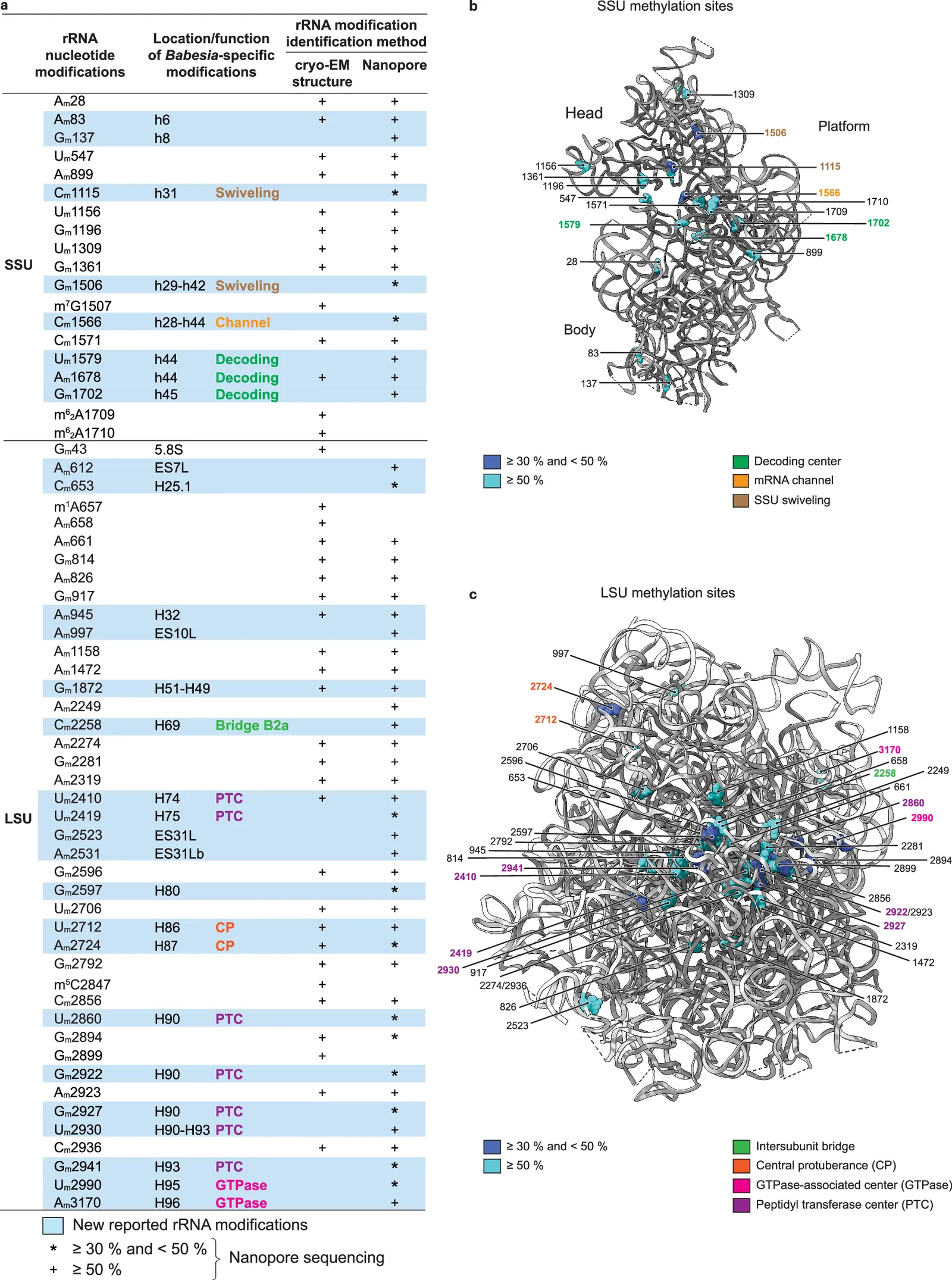
*B. divergens* ribosome rRNA methylations. **a** rRNA modification list identified in this study, either by density map analysis (cryo-EM structure) and/or nanopore sequencing methods (technical replicate). For nanopore sequencing, methylations identified at a threshold of 50% or higher are indicated by a plus sign (+). In comparison, those indicated with an asterisk (*) correspond to modifications identified following an applied threshold between 30 and 50%. A hyphen (–) relates to a modification located between two rRNA helices (e.g., h29–h42). Nucleotides 612 and 2531 are unmodeled within the ribosome. **b** Methylation sphere representation in the 3D structure of the *B. divergens* SSU, with a threshold set either between 30 and 50%, or higher than 50%. Base methylations and ribosomal proteins are omitted for clarity. **c** Methylation sphere representation in the 3D structure of the *B. divergens* LSU, with a threshold set either between 30 and 50%, or higher than 50%. Ribosomal proteins are omitted for clarity.

By combining cryo-EM density map analysis with nanopore sequencing, we identified a total of 61 methylation sites. Among these, five corresponds to base methylations (m^7^G, m^6^_2_A (2), m^1^A, and m^5^C), which were observed in the cryo-EM density map, while the others correspond to 2’-*O*-ribose methylations (2’-*O*-Me) (Fig. 5). Out of the 56 2’-*O*-Me sites identified, three modifications were identified exclusively by cryo-EM density maps analysis (Gm43, Am658, and Gm2899), while 31 modifications were identified by cryo-EM and nanopore sequencing. Nanopore sequencing allowed the identification of 22 additional sites, among which more than 50% exceed the ≥ 50% threshold.

Irrespective of the identification method used (cryo-EM and nanopore sequencing), we identified 28 new 2’-*O*-Me sites throughout *B. divergens* cytosolic ribosomes. In the SSU, most of the 2’-*O*-Me are located within the decoding center, the mRNA channel, and the head–body interface that mediates the SSU swiveling along with tRNA dynamics (Fig. 5a, b). In the LSU, methylations cluster within the peptidyl transferase center (PTC), including helix 80 (LSU H80), which binds the amino-acid extremity (CCA-end) of the P-site tRNA. We observed 2’-*O*-Me in the central protuberance (H86 and H87), in the intersubunit bridge B2a, located between the A- and P-tRNA binding sites (H69), and in the GTPase-associated center **(**H95 and H96) (Fig. 5a, c). We also detected a subset of rRNA methylations in the *B. divergens* ESs (ES7L, ES10L, and ES31L, Fig. 5a).

**Fig. 6:**
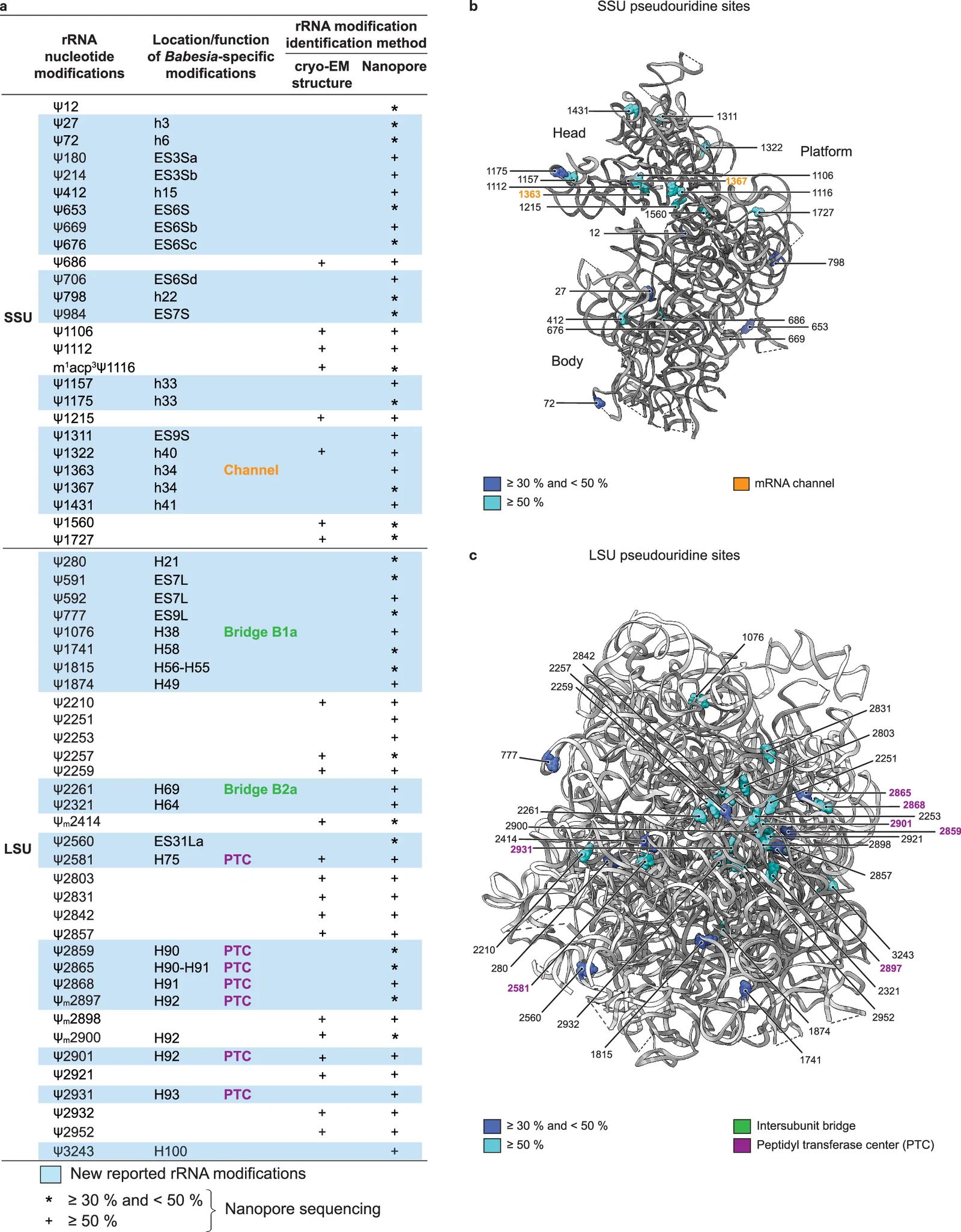
*B. divergens* ribosome rRNA pseudouridylations. **a** rRNA modification list identified in this study, either by density map analysis (cryo-EM structure) and/or nanopore sequencing methods (technical replicate). Among all the pseudouridylations observed by cryo-EM, all are coordinated with a planar water molecule. For nanopore sequencing, pseudouridylations identified at a threshold of 50% or higher are indicated by a plus sign (+). In comparison, those indicated with an asterisk (*) correspond to modifications identified following an applied threshold between 30 and 50%. A hyphen (–) relates to a modification located between two helices (e.g., H90-H91). Nucleotides 180, 214, 706, 984, 591 and 592 are unmodeled within the ribosome. **b** Pseudouridylations sphere representation in the 3D structure of the *B. divergens* SSU, with a threshold set either between 30 and 50%, or higher than 50%. Ribosomal proteins are omitted for clarity. **c** Pseudouridylations sphere representation in the 3D structure of the *B. divergens* LSU, with a threshold set either between 30 and 50%, or higher than 50%. Ribosomal proteins are omitted for clarity.

Pseudouridylations are more challenging to identify in cryo-EM maps because they require high local resolution to observe the characteristic hydrogen bond formed between the N1 imino group (N1H) and a water molecule, as previously described in human ribosomes (Fig. 2c)^45^. Among the various methods available to identify pseudouridine modification positions^67,70–73^, we again turned to nanopore sequencing, as described above. We identified a total of 60 pseudouridylation sites, including five pseudouridine methylations (Ψ_m_). More than half of the pseudouridines identified were present at a threshold higher than 50% (Fig. 6). The pseudouridines in the SSU are generally located within the head, including in the mRNA channel, with a large group of pseudouridines found in the truncated ESs (ES3S, ES6S, ES7S, and in ES9S) (Fig. 6a, b). In the LSU, pseudouridines are generally clustered at the PTC, including in H92, which interacts with the CCA extremity of the A-site tRNA. Pseudouridines found in the line of the PTC (H90-H91) extend toward the surface, positioning them at the interface between the catalytic core and the binding site for translation factors. Modifications were also identified in inter-subunit bridges, including in H38 (A-site finger; bridge B1a) and in H69 (bridge B2a with SSU h44). Pseudouridines were also present in the truncated LSU ESs (ES7L, ES9L, and ES31L) (Fig. 6a, c). Thus, a large proportion of the herein identified modifications (methylations and pseudouridylations) are in functional centers, rather than in purely structural elements.

## Discussion

In this study, we present high-resolution cryo-EM structures of the *B. divergens* ribosome and its rRNA modification landscape. Through our analyses of the two conformational states of the 80S ribosome, we show that the compact architecture of the *B. divergens* rRNA, particularly within the LSU, results in significant modification of its surface. This change may provide species-specific opportunities for translation regulation in *Babesia*. Along with the rRNA modification annotation for the *B. divergens* ribosome, we report modification sites enriched in functional and critical ribosomal regions, including the SSU decoding center and the LSU PTC.

High-resolution cryo-EM density maps of *B. divergens* ribosome enabled us to model all ribosomal proteins except those associated with the P stalks of the LSU^48–51^. Owing to the high-quality maps, we identified, in a few instances, discrepancies between the density and the corresponding protein sequence from the *B. divergens* 1802A reference genome annotation. Consequently, we corrected these genome annotations in the VEuPathDB, including missing introns (e.g., uS7, eS10, uS12, eL18, and eL24) and the identification of, to our knowledge, previously unannotated genes (e.g., eS17, eL41). Moreover, the cryo-EM dataset showed no corresponding density for the ribosomal protein eL28, whereas this protein is present in *P. falciparum*^31–33^ and *T. gondii* cryo-EM ribosome structures^35^. In *P. falciparum*, eL28 interacts at the junction of two expansion segments, ES7La and ES15L, both of which are much shorter in *B. divergens*. We hypothesize that the reduction of the ESs in *B. divergens* may have led to the loss of eL28. The genomes of *B. microti* and *B. duncani* include a gene encoding eL28. Although the 28S rRNA is not yet annotated in *B. duncani*, we found that in *B. microti*, the ES7La rRNA is about the same length as in *P. falciparum*, suggesting that ES7La contains the necessary rRNA elements to anchor eL28 on the *B. microti* ribosome. *Babesia divergens* has therefore simplified its ribosomal architecture over a short evolutionary period compared to *B. microti* and potentially *B. duncani*. These features collectively distinguish the *B. divergens* ribosome from those of other apicomplexans, potentially influencing ribosomal assembly, stability, and translational control in this lineage.

Our insights into the composition and evolution of *B. divergens* ribosomal proteins prompted us to examine the association of other ribosome-bound factors that have previously shown divergent profiles among apicomplexan parasites. For example, we observed RACK1 associated with the head of the SSU, as previously described for yeast and human ribosomes^37,52,53,74^. This finding contrasts with previous studies in *P. falciparum* and *T. gondii*, in which RACK1 density was absent or weak and unmodeled^31,32,34,35^. The robust presence of RACK1 may reflect either improved sample preservation during freezing and imaging or the heterogeneity of the host-parasite life stages sampled, given that the parasites in this study were grown asynchronously. The latter suggestion is supported by a recent study in which in situ structures of ribosomes from cryogenic-focused ion beam (cryo-FIB) milled *P. falciparum*-infected human erythrocytes across all asexual stages show the presence of RACK1 on the SSU, although the lower resolution limited the detailed characterization of the interaction^33^. In contrast, a recent cryo-EM ribosome structure in *Plasmodium* suggests that artemisinin treatment increases the association of RACK1 with the SSU. Given the well-established biological importance of RACK1 association with ribosomes in other eukaryotic systems^75^, additional work will be required to delineate how RACK1 association with apicomplexan ribosomes influences their functional specialization.

In this study, we also present a comprehensive mapping of rRNA modifications in *B. divergens*. By integrating nanopore sequencing with high-resolution cryo-EM structure determination, we identified an extensive set of methylation and pseudouridylation sites. We identified rRNA modifications at functional sites within the SSU, including the decoding center, mRNA channel, and intersubunit bridges b1a and b2a, as well as within the LSU, including the PTC and the GTPase-associated region. Several of the modifications identified are within the truncated ESs. Although the biological consequences of these modifications remain to be fully elucidated, their presence at functional hotspots points toward roles in modulating translation. Consistent with this observation, a single *Leishmania* pseudouridylation in H69 (Ψ512) (*L. donovani* and *L. major*) has been shown to be preferentially hypermodified in the host stage (amastigotes). This pseudouridylation affects tRNA selection, thereby increasing the translation of mRNAs that carry high-biased codons^26^. Interestingly, the corresponding site in *B. divergens* (Ψ2259) is also highly modified in the *B. divergens* ribosome structure from parasites maintained in human blood culture, but whether it is hypomodified in the vector stage is unknown.

Beyond their potential impact on ribosome function and translation, unique rRNA modifications may also influence the susceptibility of *B. divergens* ribosomes to small-molecule inhibitors. Several compounds targeting apicomplexan translation machinery have been described to date^76^. For example, mefloquine has been shown to bind the GTPase-associated center of the *Plasmodium* cytosolic ribosome, and certain derivatives exhibit enhanced potency by exploiting *P. falciparum*-specific ribosomal residues^36^. Emetine, a well-characterized translation inhibitor, binds to the ribosomal E-site and disrupts translocation, but its clinical application is limited by its high toxicity^32^. Similarly, pactamycin, another E-site binder, has inspired the development of analogs with reduced cytotoxicity that retain potent antimalarial activity^77^. Collectively, these studies highlight that the translational machinery is a promising yet underexplored target for antiparasitic drug development, analogous to strategies employed against bacterial pathogens.

## Methods

### Parasite culture

We cultivated *B. divergens* Rouen 1987 strain at high parasitemia ( > 20%), 37 °C, and 4% hematocrit (HCT) in purified Caucasian male O-positive human red blood cells (RBCs from Research Blood Components) under low-oxygen conditions (1% O_2_, 5% CO_2_). We maintained cells in RPMI-1640 medium (Thermo Fisher Scientific) supplemented with 25 mM HEPES, 367.5 µM hypoxanthine, and 25 µg/mL gentamicin (to maintain sterility in parasite cultures without compromising viability) at pH 6.75. This medium was then supplemented with 22.2 mM sodium bicarbonate and 4.31 mg/mL AlbuMAX II (Invitrogen). We isolated parasites from RBCs by mechanical release, followed by filtration through a 1.2 µm Luer-lock syringe filter and centrifugation at 3000 x *g* for five min^78,79^. We processed a 30 mL culture volume (4% HCT, 1.2 mL packed RBCs) according to this procedure, which we repeated for a total of three liters of culture volume. To isolate free merozoites from the supernatant, we centrifuged at 4100 × *g* for 15 min.

### Ribosome purification

We washed parasite pellets twice with 1X PBS at 16,000 x *g* for five min. We then resuspended the pellet in a lysis buffer (LB: 20 mM HEPES pH 7.4, 300 mM KCl, 25 mM Mg(CH_3_COO)_2_, 0.15% Triton X-100, 5 mM 2-β-mercaptoethanol, and complete protease inhibitor tablet [Roche]), incubated at 4 °C for one hour, followed by two freeze-thaw cycles. We centrifuged the lysates at 12,000 x *g* for 10 min. We loaded the supernatant on a sucrose cushion buffer (SCB: 20 mM HEPES pH 7.4, 150 mM KCH_3_COO, 10 mM NH_4_CH_3_COO, 10 mM Mg(CH_3_COO)_2_, 30% (w/v) sucrose, and 5 mM 2-β-mercaptoethanol). We centrifuged at 4 °C for 16 h at 146,944 x *g* in a 70.1 Ti rotor (Beckman Coulter). We resuspended the crude ribosomal pellet in 300 µL of ribosome buffer (RB: SCB devoid of sucrose) and then centrifuged it at 14,000 x *g* for 15 min. We layered the supernatant onto a 10–40% (w/v) sucrose gradient (Biocomp Gradient Master) and centrifuged at 4 °C for 3 h at 288,056 x *g* using an SW41 Ti rotor (Beckman Coulter). We collected and pooled the fractions containing ribosomes according to the UV absorbance profile (Biocomp Gradient Fractionator equipped with a Triax flow cell). We back-exchanged the buffer into the RB described above using an Amicon Ultra-4 centrifugal filter unit with a 50 kDa cutoff (Millipore).

### Data collection by cryo-EM

We glow-discharged cryo-EM grids (Quantifoil R1.2/1.3 with Ultrathin Carbon) using a Pelco Easiglow plasma discharge system (Ted Pella) at 15 mA for 30 s. We plunge-freeze glow-discharged cryo-EM grids, previously incubated with 3 µL of *B. divergens* ribosomes ( ~ 100 nM) for one min, using a FEI Vitrobot Mark IV (Thermo Fisher Scientific) operating at 100% humidity, 4 °C, and a blot force of 12 for 3 s. We then transferred the grids to an FEI Titan Krios electron microscope, operated at 300 kV, using Serial EM for automated data acquisition^80^. We collected one data set (Supplementary Tables 1 and 2) at a nominal underfocus of −0.8 to −2.2 µm at a magnification of 105,000 x, yielding a pixel size of 0.825 Å. We recorded the micrographs as a movie stack (12,114 frame stacks/movies) on a K3 direct electron detector (Gatan) operated in counting mode. We collected 40 frames per 1.4-s movie, with a total dose of 42 e/Å^2^.

### Single-particle image processing

We gain corrected, aligned (5 × 5 patch), and dose-weighted the dose-fractionated movies using MotionCor2^81^. We used the unweighted, aligned micrographs for CTF estimation with CTFFIND-4.1.13^82^. We used crYOLO to select 1,259,788 particles^83^. We used the software RELION 3.1.2 for 2D and 3D classification and refinements^84–86^. We extracted the particles with a box size of 448 pixels (370 Å) and binned them fourfold. We split the particles into three subsets (size split 500,000), and each subset was subjected to two rounds of 2D classification. We used a subset of the well-resolved 2D class averages (418,357 particles) for the ab initio 3D reconstruction model. We used this model as a starting reference (filtered to 40 Å) for a 3D consensus refinement. We then subjected each subset to 3D classification without alignments and selected 3D classes that showed well-formed ribosome particles. We created a mask of the inter-subunit space (PDB 6GZ3)^87^ and carried out focused classification. We reextracted the particles at the original pixel size. We refined each class independently using an initial angular sampling set of 15 degrees and local searches from an auto sampling set at 3.7 degrees. Three classes displayed clear morphology for 80S particles. We initially selected the most stable and populated class (3D Class 3) and did per-particle CTF refinement, aberration refinement, followed by signal-subtracted focused refinement and classification without alignments^88^. We then did another round of 3D auto-refinement. Before sharpening (post-processing), we created a mask for the 80S using a 15 Å low-pass filter and an initial binarization threshold of 0.003. We extended the binary map by 4 pixels in every direction. We added a cosine-shaped soft edge of 4 pixels, resulting in an 80S map with a global estimated resolution of 2.7 Å^89–91^. We performed a multibody refinement to improve the resolution of the distinct subdomains, focusing on the SSU head and body and the LSU regions separately^92^. We combined the resulting focus-refined maps using ChimeraX^40^ to generate a full 80S map. We also tried partial signal subtraction with centering (center-subtracted images on mask option enabled), followed by focused refinement of the SSU head^93^. Still, it did not result in any additional interpretable density for model building compared with the map obtained from multibody refinement. We also further processed a class (3D Class 1) containing density for E-, P-, and A-site tRNAs and mRNA. Briefly after a round of 3D auto-refinement, we performed a focused classification (without alignments) of the tRNAs to isolate a more homogeneous subset, followed by another round of 3D auto-refinement. We then performed CTF and aberration refinement, and re-refined and post-processed the map using an 80S mask (low-pass filtered at 15 Å, binarization threshold 0.004, 4-pixel extension, and 4-pixel soft edge), yielding a global estimated resolution of 3.1 Å relative to the gold-standard FSC criterion. Multibody refinement was also used to improve the resolution of individual subdomains, and a composite map was generated for this class.

### Atomic model building and refinement

We retrieved ribosomal protein sequences from *B. divergens* 1802A (the reference strain) using *P. falciparum* 3D7 sequences (PDB IDs: 3J7A, 3J79)^32^ and the BLAST tool from PiroplasmaDB and PlasmoDB, a part of the VEuPathDB^94^. We input the extracted *B. divergens* sequences into Alphafold2 using the Colab server with template and amber relaxation options enabled^39^. We used AlphaFold2-validated metrics to assess model prediction reliability and selected the best-relaxed structures with the highest pLDDT for docking into the post-processed *B. divergens* maps using ChimeraX^40^. We retrieved rRNA sequences from *B. divergens* using the RNAcentral database^41^. We aligned the sequences with those of *T. gondii* structures (PDB IDs: 5XXB, 5XXU)^35^ using Clustal Omega^42^. We used predicted secondary structure maps from RNAcentral to aid in rRNA modeling and mutated individual rRNA residues based on the *B. divergens* sequence. We built de novo less conserved regions and linker sequences using Coot^95^. We reviewed base-pairing after manual adjustment with Coot. We initially focused on building the atomic model corresponding to ribosomes bound to an E-site tRNA (Conformational state 2), given the highest-resolution maps. We refined the atomic model against the corresponding density map using real-space refinement (phenix.real_ space_refine) in PHENIX^44^. We applied iterative rounds of manual model adjustment in Coot and model refinement in PHENIX. The E-site tRNA (PDB 3J7A)^32^ was docked into the ribosome using ChimeraX and further refined in PHENIX. We evaluated the quality of the final model using MolProbity^96^. We also evaluated the overall fit between the atomic model and the composite map using the *Q*-score metric^97^ (overall *Q*_model per chain = 0.600). We docked this model into 3D Class 1, docked the P and A-site tRNAs (PDB 9BUP), and then manually rebuilt the rRNA. We used ModelAngelo^98^ to generate ribosomal protein coordinates, which showed local rearrangements compared to the 3D Class 3. The model was further refined with iterative rounds of manual adjustment in Coot and real-space refinement as described above (overall *Q*_model per chain = 0.524). Based on our atomic ribosome models, we improved the VEuPathDB annotation set in PiroplasmaDB through manual curation in Apollo^94^, by correcting misannotations of gene models coding for ribosomal proteins uS7, eS10, uS12, eL15, eL18, eL24, and eL41.

### Mass spectrometry sample preparation

We denatured ribosomal proteins with Laemmli buffer supplemented with 2-β-mercaptoethanol to *B. divergens* ribosomes (1:1 ratio), previously purified, and boiled at 100 °C for three min. We precipitated the proteins by adding five volumes of 0.1 M ammonium acetate in 100% methanol to the Laemmli-containing samples. We centrifuged the samples at 12,000 x *g* at 4 °C for 15 min. We washed the resulting protein pellet twice with 0.1 M ammonium acetate in 80% methanol and dried the pellet under vacuum (Speed-Vac concentrator). We submitted the dried pellet to the Harvard Center for Mass Spectrometry (HCMS). A dried protein sample of 5 µg was dissolved in 100 µL of 50 mM triethylammonium bicarbonate (TEAB) and reduced by the addition of 20 mM tris(2-carboxyethyl) phosphine (TCEP) and incubated at 37 °C for 45 min. Proteins were alkylated in 40 mM iodoacetamide for 45 min in the dark at room temperature. Proteins were trypsinized at a 1:50 ratio (w/w, substrate/enzyme) and incubated overnight at 37 °C. Digested samples were cleaned with C18 Spin Tips (Pierce, Thermo Scientific) and then reconstituted with 0.1% formic acid before LC-MS/MS analysis.

### LC-MS/MS analysis

LC-MS/MS experiments (*n* = 1 sample analyzed) were performed on a Lumos Tribrid Orbitrap Mass Spectrometer equipped with Ultimate 3000 (Thermo Fisher) nano-HPLC. Peptides were separated on a 150 μm inner diameter microcapillary trapping column packed with approximately 2 cm of C18 Reprosil resin (5 μm, 100 Å, Dr. Maisch GmbH, Germany), followed by an analytical column (µPAC, C18 pillar surface, 50 cm bed, Thermo Scientific). Separation was achieved by applying a 5–35% acetonitrile gradient in 0.1% formic acid over 180 min at a flow rate of 300 nL/min. Electrospray ionization occurred with a voltage of 2 kV using a homemade electrode junction at the end of the microcapillary column, sprayed from metal tips (PepSep, Denmark). The mass spectrometry survey scan was performed in the Orbitrap in the range of 410–1800 m/z at a resolution of 12 × 10^4^, followed by the selection of the twenty most intense ions (TOP20) for CID-MS2 fragmentation in the Ion trap using a precursor isolation width window of 2 m/z, AGC setting of 10,000, and a maximum ion accumulation of 100 ms. Singly charged ion species were not subjected to CID fragmentation. The normalized collision energy was set to 35 V with an activation time of 10 ms. Ions in a ten-ppm m/z window around ions selected for MS2 were excluded from further selection for fragmentation for 60 s. Raw data from LC-MS/MS experiments were submitted for analysis in Proteome Discoverer 2.4 software (Thermo Scientific). The MS/MS data were searched against the *B. divergens* 1802A annotated protein sequence dataset^21^ from PiroplasmaDB and against known contaminants, including human keratins and typical laboratory contaminants. Sequest HT searches were performed using the following guidelines: a 15 ppm MS tolerance and 0.6 Da MS/MS tolerance; trypsin digestion (lysine and arginine cleavage sites) with up to two missed cleavages; carbamidomethyl group on cysteine as static modification; oxidation of methionine, N-terminal acetylation, and deamidation of asparagine and glutamine as variable modifications; and required peptide length ≥ 6 amino acids. At least one unique peptide per protein group is needed to identify proteins. An MS2 spectra assignment FDR of 1% at both the protein and peptide levels was achieved by applying a target-decoy database search with Percolator.

### rRNA purification

We cultured and harvested the parasites as described above. We used a 50 mL culture volume of *B. divergens* at 2% HCT and ~ 15–20% parasitemia. The RBCs were resuspended in 0.33% saponin to release parasites, and free merozoites were also collected from the supernatant by additional rounds of centrifugation. Pellets were washed in Dulbecco’s PBS (DPBS) and homogenized in TRI Reagent (ThermoFisher). Total RNA was extracted from *B. divergens* using a Direct-zol RNA Miniprep Kit (Zymo Research) according to the manufacturer’s instructions. We performed on-column DNase I treatment for 20 min at room temperature. We assessed the concentration and quality of the total RNA using a NanoDrop spectrophotometer and RNA Flash Gel Fragment Separation (Lonza).

### Oxford nanopore sequencing

We targeted *B. divergens* 28S and 18S rRNAs using custom oligos complementary (MilliporeSigma) to their specific 3′ ends (18S: 5′-GAGGCGAGCGGTCAATTTTCCTAAGAGCAAGAAGAAGCCGAATGATCCT-3′, 28S: 5′-GAGGCGAGCGGTCAATTTTCCTAAGAGCAAGAAGAAGCCATACACACAA-3′). These target-specific oligos were annealed to an adapter oligo (5′- /5PHOS/GGCTTCTTCTTGCTCTTAGGTAGTAGGTTC-3′) to generate functional reverse transcriptase adaptors (RTAs) according to Oxford Nanopore Technologies (ONT) sequence-specific SQK-RNA004 protocol. Briefly, the adaptor oligo and target-specific oligo were mixed in a 1:1 molar ratio at a final concentration of 1.4 µM in an annealing buffer (10 mM Tris-HCl, 50 mM NaCl, pH 7.5), heated to 95 °C for two min, and allowed to cool slowly to room temperature. We submitted the extracted total RNA samples described above, along with the custom RTAs, to the Harvard University Bauer Core Facility. The facility evaluated the precise concentration, RNA integrity, and quality using QuBit and TapeStation before proceeding to RNA library preparation (SQK-RNA004 kit) and nanopore sequencing. The 28S or 18S RNA libraries were loaded onto a PromethION flow cell (FLO-PRO004RA) twice (technical replicate). The raw signals were recorded and stored in POD5 format. Real-time basecalling was enabled in MinKNOW v24.06.15, using the high-accuracy model (v3.0.1). Reads were filtered with a minimum quality (Q) score ≥ 9.

### RNA modification analysis and statistics

We rebasecalled the raw sequencing signals (POD5) using Oxford Nanopore’s basecaller Dorado 0.9.0 in super-accurate mode (rna004_130pbs_sup@v5.1.0) with pseudouridine base detection enabled. We used Dorado v1.0.2 (*rna004_130bps_sup@v5.2.0*) to additionally detect 2′-O-methylations, a feature introduced in this version of the basecaller. We mapped the base-called reads to the *B. divergens* 18S or 28S rRNA using the Dorado aligner. The aligned reads were filtered, sorted, and indexed using SAMtools^99^. We used Modkit (v0.5.0) to identify modification sites, aggregate per-read calls, and compute counts of modified (Nmod) and unmodified reads at each position. Per-site coverage was capped (maximum depth) at 7.5 million reads. Modkit automatically determined a confidence threshold (10th percentile of the site-specific probability distribution) to exclude low-confidence basecalls. The resulting thresholds were 73.8% (C), 84.0% (U), 92.4% (G), and 75.6% (A) for 18S rRNA, and 73.0% (C), 73.4% (U), 83.4% (G), and 65.8% (A) for 28S rRNA. For pseudouridine modification calls, the thresholds were 79.1% and 77.9% for the 18S and 28S, respectively. Modification levels (occurrence) were calculated as the percentage of modified calls passing thresholds among the total number of calls at a given nucleotide (Nmod/Nvalid_cov × 100). In addition to this standard % Modified reported by Modkit we calculated an adjusted modification percentage defined as Nmod / (Nvalid_cov + Ndiff) × 100. The Ndiff counts, provided directly by Modkit, represent reads at that position that do not contain the nucleotide evaluated by the model and are therefore excluded from the default calculation (Nvalid_cov), even when such reads make up the majority of coverage. Including Ndiff in the denominator prevents inflated values at sites where the nucleotide subject to modification is rare.

### Reporting summary

Further information on research design is available in the Nature Portfolio Reporting Summary linked to this article.

## Supplementary information

Supplementary Information

Reporting Summary

Transparent Peer Review file

## Data Availability

The atomic coordinates and cryo-EM maps generated in this study have been deposited in the PDB and Electron Microscopy Data Bank (EMDB), respectively. For the conformational state 1 (3D Class 1, 80S ribosome with three tRNAs and mRNA), the PDB accession code is 9YXB with the following associated EMDB entries: EMD-73602 (composite: [https://www.ebi.ac.uk/emdb/EMD-73602]), EMD-73574 (consensus: [https://www.ebi.ac.uk/emdb/EMD-73574]), EMD-73576 (LSU: [https://www.ebi.ac.uk/emdb/EMD-73576]), EMD-73577 (SSU body: [https://www.ebi.ac.uk/emdb/EMD-73577]), EMD-73581 (SSU head: [https://www.ebi.ac.uk/emdb/EMD-73581]). For the conformational state 2 (3D Class 3, 80S ribosome with E-site tRNA), the PDB accession code is 9YGM with the following associated EMDB entries: EMD-72933 (composite: [https://www.ebi.ac.uk/emdb/EMD-72933]), EMD-72052 (consensus: [https://www.ebi.ac.uk/emdb/EMD-72052]), EMD-72055 (LSU: [https://www.ebi.ac.uk/emdb/EMD-72055]), EMD-72069 (SSU body: [https://www.ebi.ac.uk/emdb/EMD-72069]), EMD-72070 (SSU head: [https://www.ebi.ac.uk/emdb/EMD-72070]). The mass spectrometry proteomics data have been deposited to the ProteomeXchange Consortium via the PRIDE partner repository with the dataset identifier PXD069329. Nanopore sequencing datasets have been deposited in NCBI Sequence Read Archive (SRA) under BioProject ID PRJNA1358264 and SRA ID SAMN53107339.
