# Supplementary material for: Ribosomal architecture and rRNA modification landscape in the tick-borne parasite Babesia divergens
Source: Nat Commun. 2026 Aug 6;17:9475. doi: 10.1038/s41467-026-75282-4 (PMC13542104; doi:10.1038/s41467-026-75282-4)
Supplement: Supplementary file 1 [file 41467_2026_75282_MOESM1_ESM.pdf]

Supplementary Table 1 Cryo-EM data collection, refinement, and validation statistics for ribosomes with an A-, P-, E-site tRNAs and an mRNA fragment (conformational state 1, related to Figs. 1 and 2).

**Data collection and processing**					
Microscope	FEI Titan Krios				
Detector	K3 Summit Direct Electron Detector				
Detector mode	Counting				
Voltage (kV)	300				
Magnification	105,000 x				
Pixel size (Å)	0.825				
Defocus range (µm)	-0.8 to -2.2				
Electron exposure (e-/Å^2)	42				
Number of frames per movie	40				
Exposure rate (e-/Å^2/frame^{-1})	1.05				
Data collection software	SerialEM 3.8.6				
Data processing software	RELION (versions 3.1.3 and 5.0)				
Usable micrographs	12,097				
Particles extracted	1,259,788				
Particles used for reconstruction	26,467				
FSC threshold	0.143				
EMDB accession code (EMD)	73574	73576	73577	73581	73602
Map Description	Consensus	LSU	SSU body	SSU head	Composite
Map resolution (Å) global	3.1	3.0	3.0	3.1	-
Unmasked (Å, 0.143 criterion)	3.5	3.5	3.8	4.0	-
Masked (Å, 0.143 criterion)	3.1	3.0	3.0	3.1	-
PDB accession code (80S)	9YXB				
**Refinement Statistics**					
Initial model(s) used	PDB 9YGM (conformation 2 with an E-site tRNA)				
Refinement package	PHENIX 1.21.1-5286				
Model resolution (Å, 0.5 criterion)	3.1				
Map sharpening *B* factor (Å^2)	LSU (75.1), SSU body(74.0), SSU head (76.1)				
Map CC	0.80				
Model composition					
Number of chains	82				
Non-hydrogen atoms	186499				
Residues	10180				
Nucleotides	4910				
Ligands/ions	K:12, MG:124				
Water	0				
B factors (Å^2) (min/max/mean)					
Proteins	24.32/176.06/76.09				
Nucleotides	24.28/643.95/79.84				
R.m.s. deviations					
Bond lengths (Å)	0.006				
Bond angles (°)	0.732				
**Validation Statistics**					
MolProbity score	2.12				
Clash score	5.90				
Rotamer outliers (%)	3.19				
C-beta deviations	0				
CaBLAM outliers	2.14				
Ramachandran plot					
Favored (%)	93.80				
Allowed (%)	6.09				
Outliers (%)	0.11				

Supplementary Table 2 Cryo-EM data collection, refinement, and validation statistics for ribosomes with an E-site tRNA (conformational state 2, related to Figs. 1 and 2).

**Data collection and processing**					
Microscope			FEI Titan Krios		
Detector			K3 Summit Direct Electron Detector		
Detector mode			Counting		
Voltage (kV)			300		
Magnification			105,000 x		
Pixel size (Å)			0.825		
Defocus range (µm)			-0.8 to -2.2		
Electron exposure (e-/Å^2)			42		
Number of frames per movie			40		
Exposure rate (e-/Å^2/frame^{-1})			1.05		
Data collection software			SerialEM 3.8.6		
Data processing software			RELION 3.1.3		
Usable micrographs			12,097		
Particles extracted			1,259,788		
Particles used for reconstruction			261,009		
FSC threshold			0.143		
EMDB accession code (EMD)	72052	72055	72069	72070	72933
Map Description	Consensus	LSU	SSU body	SSU head	Composite
Map resolution (Å) global	2.6	2.5	2.6	2.7	-
Unmasked (Å, 0.143 criterion)	2.8	2.8	2.9	3.1	-
Masked (Å, 0.143 criterion)	2.6	2.5	2.6	2.7	-
PDB accession code (80S)			9YGM		
**Refinement Statistics**					
Initial model(s) used			Alphafold2 (ribosomal proteins), PDB 5XXB and 5XXU (rRNA)		
Refinement package			PHENIX 1.21.1-5286		
Model resolution (Å, 0.5 criterion)			2.8		
Map sharpening *B* factor (Å^2)			LSU (93.9), SSU body(60.1), SSU head (64.5)		
Map CC			0.84		
Model composition					
Number of chains			80		
Non-hydrogen atoms			189059		
Residues			10898		
Nucleotides			4762		
Ligands/ions			ZN: 5, K: 26, MG: 136, IAS:1		
Water			34		
B factors (Å^2) (min/max/mean)					
Proteins			2.54/232.78/68.22		
Nucleotides			1.18/188.88/60.92		
R.m.s. deviations					
Bond lengths (Å)			0.006		
Bond angles (°)			0.641		
**Validation Statistics**					
MolProbity score			1.36		
Clash score			2.35		
Rotamer outliers (%)			1.65		
C-beta deviations			0		
CaBLAM outliers			1.47		
Ramachandran plot					
Favored (%)			96.96		
Allowed (%)			2.90		
Outliers (%)			0.14		

Supplementary Table 3 Reference table of the *B. divergens* ribosomal proteins from the small subunit (related to Fig. 1). The chain IDs correspond to the ribosomal proteins (r-prot.) modeled in the 80S ribosome structure as identified by the PDB code 9YGM. The sequence alignment identity percentage is provided for comparison between *B. divergens* 1802A and *P. falciparum* 3D7, or between *B. bovis* T2Bo and PF3D7, for eS17.

Gene ID (*Babesia*)	Unified r-prot. name	Chain ID	Number of residues modeled/sequence	Gene ID (*Plasmodium*)	Sequence residue number	Sequence alignment identity (%)
Bdiv_040040	eS1	SA	215/264	PF3D7_0322900	262	66
Bdiv_033120	uS2	SB	196/274	PF3D7_1026800	263	67
Bdiv_011570	uS3	SC	209/223	PF3D7_1465900	221	79
Bdiv_010520c	uS4	SD	178/184	PF3D7_0520000	189	85
Bdiv_034010	eS4	SE	260/266	PF3D7_1105400	261	60
Bdiv_029510	uS5	SF	180/196	PF3D7_1447000	272	80
Bdiv_037560c	eS6	SG	216/239	PF3D7_1342000	306	62
Bdiv_017820c	uS7	SH	178/192	PF3D7_0721600	195	85
Bdiv_032090	eS7	SI	178/194	PF3D7_1302800	194	51
Bdiv_016440c	uS8	SJ	129/130	PF3D7_0316800	130	79
Bdiv_012600	eS8	SK	181/192	PF3D7_1408600	218	54
Bdiv_027730	uS9	SL	134/149	PF3D7_0813900	144	62
Bdiv_009240	uS10	SM	97/120	PF3D7_1003500	118	59
Bdiv_032170	eS10	SN	96/113	PF3D7_0719700	137	65
Bdiv_010180c	uS11	SO	125/157	PF3D7_0516200	151	92
Bdiv_040390c	uS12	SP	143/145	PF3D7_0306900	145	86
Bdiv_040260c	eS12	SQ	115/135	PF3D7_0307100	141	47
Bdiv_027650	uS13	SR	144/154	PF3D7_1126200	156	71
Bdiv_036780	uS14	SS	50/66	PF3D7_0705700	54	65
Bdiv_001750c	uS15	ST	149/151	PF3D7_1358800	151	67
Bdiv_008760	uS17	SU	151/156	PF3D7_0317600	161	78
BBOV_III001250*	eS17	SV	79/134	PF3D7_1242700	137	73
Bdiv_002790	uS19	SW	118/149	PF3D7_1317800	145	67
Bdiv_040250	eS19	SX	159/174	PF3D7_0422400	170	47
Bdiv_033240c	eS21	SY	78/79	PF3D7_1144000	82	55
Bdiv_010070	eS24	SZ	96/135	PF3D7_0519400	133	60
Bdiv_022980c	eS25	Sa	75/104	PF3D7_1421200	105	48
Bdiv_038810c	eS26	Sb	97/115	PF3D7_0217800	107	70
Bdiv_036570	eS27	Sc	70/82	PF3D7_1308300	82	72
Bdiv_019090c	eS28	Sd	63/67	PF3D7_1461300	67	82
Bdiv_039110c	eS30	Se	40/61	PF3D7_0219200	58	72
Bdiv_033080	eS31	Sf	64/77	PF3D7_1402500	73	63
Bdiv_010290	RACK1	Sg	322/323	PF3D7_0826700	323	63

* *B. bovis* corresponding sequence in *B. divergens* Rouen 1987 is within CCSG02000001.1: 1890202..1890772 supercontig.

Supplementary Table 4 Reference table of the *B. divergens* ribosomal proteins from the large subunit (related to Figs. 1 and 3). The chain IDs correspond to the ribosomal proteins (r-prot.) modeled in the 80S ribosome structures as identified by the PDB code 9YGM. The sequence alignment identity percentage is provided for comparison between *B. divergens* 1802A and *P. falciparum* 3D7, or between *T. gondii* ME49 and PF3D7, for eL41.

Gene ID (*Babesia*)	Unified r-prot. name	Chain ID	Number of residues modeled/sequence	Gene ID (*Plasmodium*)	Sequence residue number	Sequence alignment identity (%)
Bdiv_038620	uL1	LA	217/217	PF3D7_1441200	217	65
Bdiv_015470	uL2	LB	248/257	PF3D7_0516900	260	69
Bdiv_003320c	uL3	LC	381/395	PF3D7_1027800	386	74
Bdiv_004040c	uL4	LD	343/360	PF3D7_0507100	411	50
Bdiv_027640	uL5	LE	166/171	PF3D7_0719600	173	79
Bdiv_027090	uL6	LF	186/190	PF3D7_1323100	190	64
Bdiv_030770c	eL6	LG	149/212	PF3D7_1338200	221	35
Bdiv_016730	eL8	LH	224/285	PF3D7_1424400	283	53
Bdiv_028610c	uL10	N.M.	0/313	PF3D7_1130200	316	57
Bdiv_010230c	uL11	N.M.	0/165	PF3D7_0517000	165	66
Bdiv_010650	uL13	LI	201/202	PF3D7_1004000	202	63
Bdiv_027720c	eL13	LJ	196/203	PF3D7_0814000	215	43
Bdiv_035030	uL14	LK	129/139	PF3D7_1331800	139	80
Bdiv_023300	eL14	LL	128/133	PF3D7_1431700	165	51
Bdiv_034950	uL15	LM	146/147	PF3D7_0618300	148	64
Bdiv_018330	eL15	LN	202/204	PF3D7_0415900	205	74
Bdiv_016810c	uL16	LO	212/227	PF3D7_1414300	219	72
Bdiv_016720c	uL18	LP	289/306	PF3D7_1424100	294	53
Bdiv_037540	eL18	LQ	186/222	PF3D7_1341200	187	61
Bdiv_001430	eL19	LR	175/194	PF3D7_0614500	182	63
Bdiv_037530	eL20	LS	184/188	PF3D7_1341200	184	57
Bdiv_016780c	eL21	LT	154/160	PF3D7_1426000	161	46
Bdiv_004980c	uL22	LU	152/194	PF3D7_1351400	203	56
Bdiv_011350c	eL22	LV	97/122	PF3D7_0821700	139	58
Bdiv_039940	uL23	LW	118/153	PF3D7_1323400	190	53
Bdiv_032900	uL24	LX	123/137	PF3D7_0312800	126	61
Bdiv_036590c	eL24	LY	61/156	PF3D7_1309100	162	54
Bdiv_016640	eL27	LZ	138/146	PF3D7_1460700	146	50
N.A.	eL28	N.D.	-	PF3D7_1142500	127	-
Bdiv_020740c	uL29	La	119/123	PF3D7_1124900	124	46
Bdiv_016690	eL29	Lb	53/59	PF3D7_1460300	67	70
Bdiv_040380c	uL30	Lc	215/259	PF3D7_0307200	257	55
Bdiv_034830c	eL30	Ld	93/108	PF3D7_1019400	108	61
Bdiv_021180	eL31	Le	105/117	PF3D7_0503800	120	55
Bdiv_038500	eL32	Lf	125/132	PF3D7_0903900	131	53

Bdiv_039460	eL33	Lg	108/115	PF3D7_1142600	140	45
Bdiv_019820c	eL34	Lh	99/150	PF3D7_0710600	150	46
Bdiv_009850c	eL36	Li	108/116	PF3D7_1109900	112	47
Bdiv_037200	eL37	Lj	88/98	PF3D7_0706400	92	66
Bdiv_028600c	eL38	Lk	65/70	PF3D7_1130100	87	31
Bdiv_008570	eL39	Ll	49/59	PF3D7_0611700	51	61
Bdiv_024380c	eL40	Lm	50/55	PF3D7_1365900	52	76
TGME49_213580*	eL41	Ln	38/39	PF3D7_1144300	39	95
Bdiv_030880c	eL42	Lo	97/105	PF3D7_0304400	104	74
Bdiv_029900c	eL43	Lp	93/94	PF3D7_0210100.1	96	68
Bdiv_033480c	P1	N.M.	0/117	PF3D7_1103100	118	50
Bdiv_035540	P2	N.M.	0/61	PF3D7_0309600	112	43

N.A. Ribosomal protein not annotated in PiroplasmaDB, and no sequence was retrieved with a Blast search using *P. falciparum*'s sequence.
N.D. Unmodeled ribosomal protein because no density was observed within the cryo-EM ribosome map.
N.M. Unmodeled ribosomal proteins within the cryo-EM ribosome map because of poor density.
* *T. gondii* corresponding sequence in *B. divergens* 1802 is within JAHBMH010000034: 30674..30988 supercontig.

Supplementary Table 5 Ribosomal proteins identification by LC-MS/MS from the *B. divergens* small subunit (related to Fig. 1). Sample purified from 80S ribosomes as identified by the PDB code 9YGM.

**Gene ID *B. divergens* 1802A**	**Unified r-prot. name**	**Coverage (%)**	**Length (amino acids)**	**Molecular weight (kDa)**	**Number of peptides**	**Spectral counts**
Bdiv_040040	eS1	49	264	30.0	15	216
Bdiv_033120	uS2	27	274	31.0	6	89
Bdiv_011570	uS3	53	223	24.7	10	188
Bdiv_010520c	uS4	35	184	21.6	8	70
Bdiv_034010	eS4	31	266	30.1	8	114
Bdiv_029510	uS5	30	196	21.1	5	48
Bdiv_037560c	eS6	44	239	27.5	11	168
Bdiv_017820c	uS7	38	220	24.7	10	79
Bdiv_032090	eS7	18	194	22.0	8	69
Bdiv_016440c	uS8	13	130	14.8	2	12
Bdiv_012600	eS8	54	192	21.9	9	121
Bdiv_027730	uS9	44	149	16.7	7	52
Bdiv_009240	uS10	36	120	13.5	6	41
**Bdiv_032170**	eS10	0	134	15.5	0	0
Bdiv_010180c	uS11	54	157	16.6	10	121
Bdiv_040390c	uS12	53	188	20.8	3	65
Bdiv_040260c	eS12	65	135	14.7	7	44
Bdiv_027650	uS13	34	154	17.5	6	82
Bdiv_036780	uS14	23	66	7.5	2	11
Bdiv_001750c	uS15	32	151	17.4	6	41
Bdiv_008760	uS17	19	156	18.1	5	21
**N.A.**	eS17	0	132	15.4	0	0
Bdiv_002790	uS19	44	149	17.2	4	42
Bdiv_040250	eS19	49	174	20.1	8	90
Bdiv_033240c	eS21	44	79	8.7	4	29
Bdiv_010070	eS24	65	135	15.6	5	51
Bdiv_022980c	eS25	22	104	11.9	2	15
Bdiv_038810c	eS26	30	115	13.2	3	19
Bdiv_036570	eS27	29	82	9.1	2	26
Bdiv_019090c	eS28	36	67	7.5	2	74
Bdiv_039110c	eS30	72	61	6.8	1	4
Bdiv_033080	eS31	54	157	17.3	1	1
Bdiv_010290	RACK	38	323	35.5	11	161

N.A. Ribosomal protein not annotated in *B. divergens* 1802A.
Ribosomal proteins undetected by mass spectrometry analysis are indicated in bold.

Supplementary Table 6 Ribosomal proteins identification by LC-MS/MS from the *B. divergens* large subunit (related to Fig. 1). Sample purified from 80S ribosomes as identified by the PDB code 9YGM.

**Gene ID *B. divergens* 1802A**	**Unified r-prot. name**	**Coverage (%)**	**Length (amino acids)**	**Molecular weight (kDa)**	**Number of peptides**	**Spectral counts**
Bdiv_038620	uL1	49	217	24.4	8	138
Bdiv_015470	uL2	42	257	27.9	9	150
Bdiv_003320c	uL3	43	395	44.5	16	161
Bdiv_004040c	uL4	44	360	39.4	13	186
Bdiv_027640	uL5	29	171	19.7	5	58
Bdiv_027090	uL6	36	190	21.4	6	139
Bdiv_030770c	eL6	43	212	23.6	9	136
Bdiv_016730	eL8	38	285	32.1	11	178
Bdiv_028610c	uL10	26	313	34.0	9	97
Bdiv_010230c	uL11	38	165	18.1	7	43
Bdiv_010650	uL13	33	202	23.3	8	115
Bdiv_027720c	eL13	28	203	22.9	7	63
Bdiv_035030	uL14	56	139	14.8	7	79
Bdiv_023300	eL14	27	133	14.9	4	12
Bdiv_034950	uL15	20	147	16.5	3	31
Bdiv_018330	eL15	16	198	23.7	3	47
Bdiv_016810c	uL16	28	227	25.7	6	37
Bdiv_016720c	uL18	41	306	35.3	11	213
Bdiv_037540	eL18	18	247	27.1	4	49
Bdiv_001430	eL19	18	194	22.6	6	83
Bdiv_037530	eL20	53	188	21.6	10	126
Bdiv_016780c	eL21	45	160	18.4	10	157
Bdiv_004980c	uL22	18	194	22.1	3	34
Bdiv_011350c	eL22	35	122	14.2	3	64
Bdiv_039940	uL23	26	153	17.3	4	33
Bdiv_032900	uL24	40	137	16.1	7	123
Bdiv_036590c	eL24	19	199	22.1	4	44
Bdiv_016640	eL27	42	146	16.8	6	126
**N.A.**	eL28	0	-	-	0	0
Bdiv_020740c	uL29	37	123	14.4	6	65
**Bdiv_016690**	eL29	0	59	6.8	0	0
Bdiv_040380c	uL30	21	259	30.3	5	51
Bdiv_034830c	eL30	56	108	11.9	4	43
Bdiv_021180	eL31	45	117	13.7	3	7
Bdiv_038500	eL32	24	132	15.4	3	9
Bdiv_039460	eL33	30	115	13.3	4	24
Bdiv_019820c	eL34	5	150	17.0	1	2
Bdiv_009850c	eL36	34	116	13.1	4	52
Bdiv_037200	eL37	7	98	10.9	1	1
Bdiv_028600c	eL38	57	70	8.1	5	59
**Bdiv_008570**	eL39	0	59	7.2	0	0
Bdiv_024380c	eL40	29	131	14.9	3	12
**N.A.**	eL41	0	-	-	0	0
Bdiv_030880c	eL42	21	105	12.0	3	52
Bdiv_029900c	eL43	27	94	10.6	2	71
Bdiv_033480c	P1	45	117	12.1	3	27
Bdiv_035540	P2	72	61	6.5	2	22

N.A. Ribosomal protein not annotated in *B. divergens* 1802A.
Ribosomal proteins undetected by mass spectrometry analysis are indicated in bold.

Supplementary Table 7 Non-ribosomal protein identification by LC-MS/MS from *B. divergens* (related to Fig. 1). Sample purified from 80S ribosomes as identified by the PDB code 9YGM.

Gene ID *B. divergens* 1802A	PiroplasmaDB protein names	Coverage (%)	Length (amino acids)	Molecular weight (kDa)	Number of Peptides	Spectral Counts
P02769	serum albumin precursor [*Bos taurus*]	31	607	69.2	18	100
P13645	Keratin, type I cytoskeletal 10 OS=*Homo sapiens*	28	593	59.5	12	29
P35908	Keratin, type II cytoskeletal 2 epidermal OS=Homo sapiens	27	639	65.4	12	21
Bdiv_010550	splicing factor, arginine/serine-rich 3	24	235	27.5	5	15
Bdiv_027340	ATP synthase F1 beta chain	23	515	55.4	7	12
Bdiv_031040	vesicle-associated membrane protein	22	225	25.2	3	6
P08515	Glutathione S-transferase class-mu 26 kDa isozyme OS=*Schistosoma japonicum*	22	218	25.5	6	19
Bdiv_031930	NAC domain containing protein	21	208	22.3	3	5
P00761	Trypsin precursor - sus scrofa	20	741	79.4	5	15
Bdiv_016790c	NAC domain containing protein	19	156	16.7	2	6
Bdiv_037120c	hsp90 protein	15	708	81.6	7	19
P04264	Keratin, type II cytoskeletal 1 OS=*Homo sapiens*	15	644	66.0	8	19
P35527	Keratin, type I cytoskeletal 9 OS=*Homo sapiens*	15	623	62.0	6	10
Bdiv_001940	aspartate transcarbamoylase	14	382	42.9	4	10
Bdiv_007280*	RNA chaperone ProQ; FinO,	14	115	12.1	1	1

	[*Escherichia coli* (strain K12)]					
Bdiv_037240c	nucleolar GTP-binding protein 1	13	590	67.5	6	12
Bdiv_015690c	50 kDa surface antigen	13	445	47.9	5	9
Bdiv_033760	Apicomplexa-specific secreted signal peptide [*Babesia ovis*]	13	250	27.9	2	2
P81605	Dermcidin precursor - *Homo sapiens*	13	110	11.3	2	2
Bdiv_039420	BOP1NT (NUC169) domain containing protein	12	709	81.3	7	43
Bdiv_014120c	mRNA turnover protein 4 mrto4 like protein [*Babesia gibsoni*]	12	225	25.2	2	8
Bdiv_007890	actin	12	376	41.9	3	9
Bdiv_004780	transmembrane protein, putative [*Babesia ovis*]	11	234	27.6	2	2
Bdiv_031450	glycosylphosphatidylinositol-anchored merozoite surface protein	11	324	35.0	2	6
Bdiv_011020	gar1 protein RNA binding region containing protein	10	191	20.0	1	1
Bdiv_009840c*	pre-60S ribosome [*Saccharomyces cerevisiae* S288C]	9	161	18.4	1	1
Bdiv_009750	pescadillo N-terminus family protein	8	468	53.2	3	14
Bdiv_027520c	proliferation-associated protein 2g4	8	387	42.0	3	10
Bdiv_015840c	apolipoprotein N-acyltransferase [*Babesia caballi*]	8	144	15.3	1	1
Bdiv_009230c	p29 protein	8	201	23.5	1	1

Bdiv_017280c	adenine nucleotide translocase	7	299	33.4	2	6
Bdiv_002420	gliding-associated protein 45	7	184	21.4	1	2
Bdiv_032380	WD domain, G-beta repeat containing protein	7	439	48.4	3	6
Bdiv_025320c	transporter	7	467	50.7	3	6
Bdiv_035860	Translocation protein [Babesia sp. Xinjiang]	7	404	46.6	2	2
Bdiv_032250	proteasome A-type and B-type family protein	7	257	28.3	1	1
Bdiv_026360c	hypothetical protein, conserved	7	172	18.1	1	1
Bdiv_023990c	apical membrane antigen 1	6	605	67.5	3	8
Bdiv_002930c	60S ribosomal protein L7-like 1	6	234	26.5	1	2
Bdiv_018040c	proteasome A-type and B-type family protein	6	252	27.5	1	1
Bdiv_038840c	secreted antigen 1	6	533	57.7	2	2
Bdiv_032480	vacuolar protein sorting 29	6	246	27.0	1	1
Bdiv_021150c	Ser/Arg-rich splicing factor	6	199	24.0	1	3
Bdiv_005420	heat shock protein 60	6	556	59.7	2	6
Bdiv_011500	cytochrome c oxidase subunit ApiCOX25, putative [*Plasmodium yoelii*]	6	254	29.3	1	1
P00766	Chymotrypsinogen A - *Bos taurus*	6	245	25.7	1	1
Bdiv_017140	COPI associated protein, putative [*Babesia caballi*]	6	276	31.2	1	1
Bdiv_014380	secreted antigen 1	6	313	34.8	1	1
Bdiv_020050c	major facilitator superfamily MFS-1 [*Babesia ovata*]	5	486	53.3	2	8

Bdiv_018730	RNA processing factor 1	5	301	35.2	1	7
Bdiv_007850	secreted antigen 1	5	497	54.5	2	2
Bdiv_036170c	fatty acyl-CoA synthetase family protein	5	673	74.7	2	2
Bdiv_011410c	cytochrome oxidase subunit I, putative [*Babesia ovata*]	5	311	34.2	1	2
Bdiv_040150	Mak16 family protein	5	222	26.2	1	2
Bdiv_019270	proliferating cell nuclear antigen 1	5	277	30.8	1	1
Bdiv_031730c	membrane protein	5	367	40.5	1	1
Bdiv_025500c	erythrocyte binding protein 37.2 precursor	5	316	34.7	1	1
Bdiv_003280	Brix domain containing protein	4	323	36.5	1	1
Bdiv_009700	Ribosome biogenesis protein nsa1; 60S [*Schizosaccharomyces*]	4	590	66.1	2	3
Bdiv_036030	Pre mRNA splicing factor CWF19, cell cycle control protein, putative [*Babesia bigemina*]	4	310	35.6	1	1
Bdiv_006980c	hypothetical protein, conserved	4	241	25.8	1	1
Bdiv_033560c	enolase (2-phosphoglycerate dehydratase)	4	442	48.0	1	2
Bdiv_013400c	secreted antigen 1	4	567	62.3	1	2
Bdiv_025200c	aspartyl aminopeptidase	4	447	49.3	1	2
2497269	Keratin, type i cytoskeletal 12 [*Homo sapiens*]	4	494	53.5	1	1
Bdiv_036250c	2-methylcitrate synthase [*Babesia caballi*]	4	170	19.0	1	1

Bdiv_039450c	eukaryotic porin protein [*Babesia caballi*]	4	298	32.8	1	1
Bdiv_023170	glutamate dehydrogenase	3	455	50.6	1	1
Bdiv_003860c	rhomboid 4	3	632	69.8	1	1
Bdiv_003340	Clathrin interactor EPSIN 1 [*Babesia sp. Xinjiang*]	3	462	51.9	1	2
Bdiv_011370c	PCI domain containing protein	3	431	48.9	1	2
Bdiv_012730c	CAAX metallo endopeptidase	3	448	52.1	1	2
Bdiv_036160c	secreted antigen 1	3	566	62.1	1	2
Bdiv_022170c	nonsense-mediated mRNA decay protein	3	493	56.5	1	1
Bdiv_005070	nucleolar GTP-binding protein 2	3	636	71.9	1	1
Bdiv_012850	putative protein phosphatase 2C 8 [*Babesia sp. Xinjiang*]	3	393	45.2	1	1
Bdiv_025360c	transcription or splicing factor-like protein	3	483	54.1	1	1
P08659	Luciferin 4-monooxygenase Firefly luciferase OS=*Photinus pyralis*	3	550	60.7	1	1
Bdiv_019870	GTP binding protein	3	423	46.8	1	1
Bdiv_003940	sulfate transporter	3	723	80.1	1	1
Bdiv_003010c	secreted antigen 1	3	298	33.4	1	1
Bdiv_011800	membrane protein	2	716	80.4	1	2
Bdiv_002620	myb-like DNA-binding domain containing protein	2	537	61.5	1	1
Bdiv_036530	ATP synthase [*Babesia duncani*]	2	544	62.7	1	2
Bdiv_018060*	Mitochondrial RNA binding complex 1	2	643	71.3	1	2

	subunit [*Trypanosoma brucei*]					
Bdiv_036610c	carbamoyl phosphate synthetase	2	1630	179.8	2	2
Q7Z794	Keratin, type II cytoskeletal 1b OS=*Homo sapiens*	2	576	61.7	1	2
Bdiv_011530	RIBONUCLEASE Z domain containing protein, putative	2	661	74.1	1	1
Bdiv_014590c	Phosphatidate cytidylyltransferase domain-containing protein	2	543	59.9	1	2
P13647	Keratin, type II cytoskeletal 5 OS=*Homo sapiens*	2	590	62.3	1	1
Bdiv_024840	WD repeat domain containing protein	2	848	94.6	1	1
Bdiv_034280	DNA repair protein (mre11) family protein	2	967	108.0	1	1
Bdiv_034410c*	mL113 Mitochondria [*Chlamydomonas reinhardtii*]	2	597	67.4	1	1
547751	Keratin, type i cytoskeletal 17 (cytokeratin 17) [*Homo sapiens*]	2	432	48.1	1	1
Bdiv_010860	micro-fibrillar-associated protein 1 C-terminus containing protein	2	450	51.9	1	1
Bdiv_010090	eukaryotic translation initiation factor 3 subunit	2	732	85.2	1	1
Bdiv_028060c	translation initiation factor 3 subunit 10	2	990	117.0	1	1
Bdiv_031890	kynurenine 3-monooxygenase and-related flavo monooxygenas	1	1163	129.6	1	10

	e family protein, putative [*Babesia ovata*]					
Bdiv_003100	helicase	1	1920	217.0	1	1
Bdiv_012610c	ubiquitin thioesterase [*Babesia gibsoni*]	1	4400	497.2	3	3
Bdiv_012920	AP2 domain transcription factor AP2X-11 [Babesia caballi]	1	629	68.4	1	3
Bdiv_030760	ATP-dependent metalloprotease FtsH family protein	1	705	78.4	1	1
Bdiv_010010c	RNA polymerase I specific transcription initiation factor	1	1030	117.3	1	1
Bdiv_020780c	vacuolar sorting-associated protein 36 [*Babesia ovis*]	1	468	53.3	1	1
Bdiv_014910	exportin 1	1	1189	138.1	1	2
Bdiv_014470c	ubiquitin-activating enzyme E1	1	1010	113.7	1	1

* Protein name/identification resulting from Blast using HHpred interactive server for protein homology detection and structure prediction.

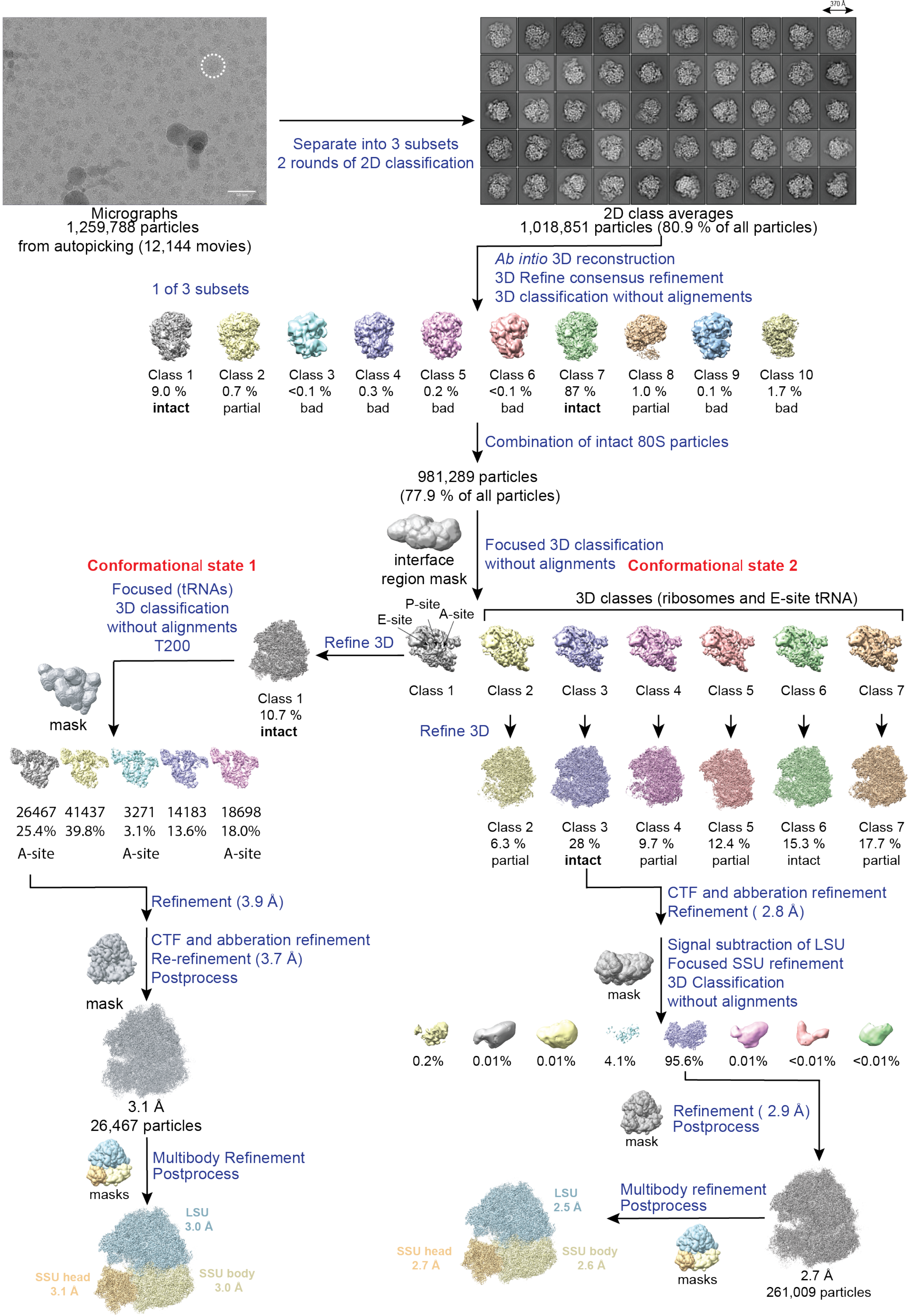

Supplementary Fig. 1 Data collection and data processing scheme for the structure of *B. divergens* ribosomes by single-particle cryo-EM (related to Fig. 1). A drift-corrected cryo-EM micrograph of purified ribosomes is shown (a white dotted circle shows a representative particle). 2D class averages show densities corresponding to the large (LSU) and the small subunits (SSU). The flowchart of the 3D classification illustrates the processing scheme used. The data was split into three subsets before 2D classification, where one of the three subsets is shown. We combined intact particles for further processing (see *Methods*). The experiments were repeated at least twice showing similar micropgraphs.

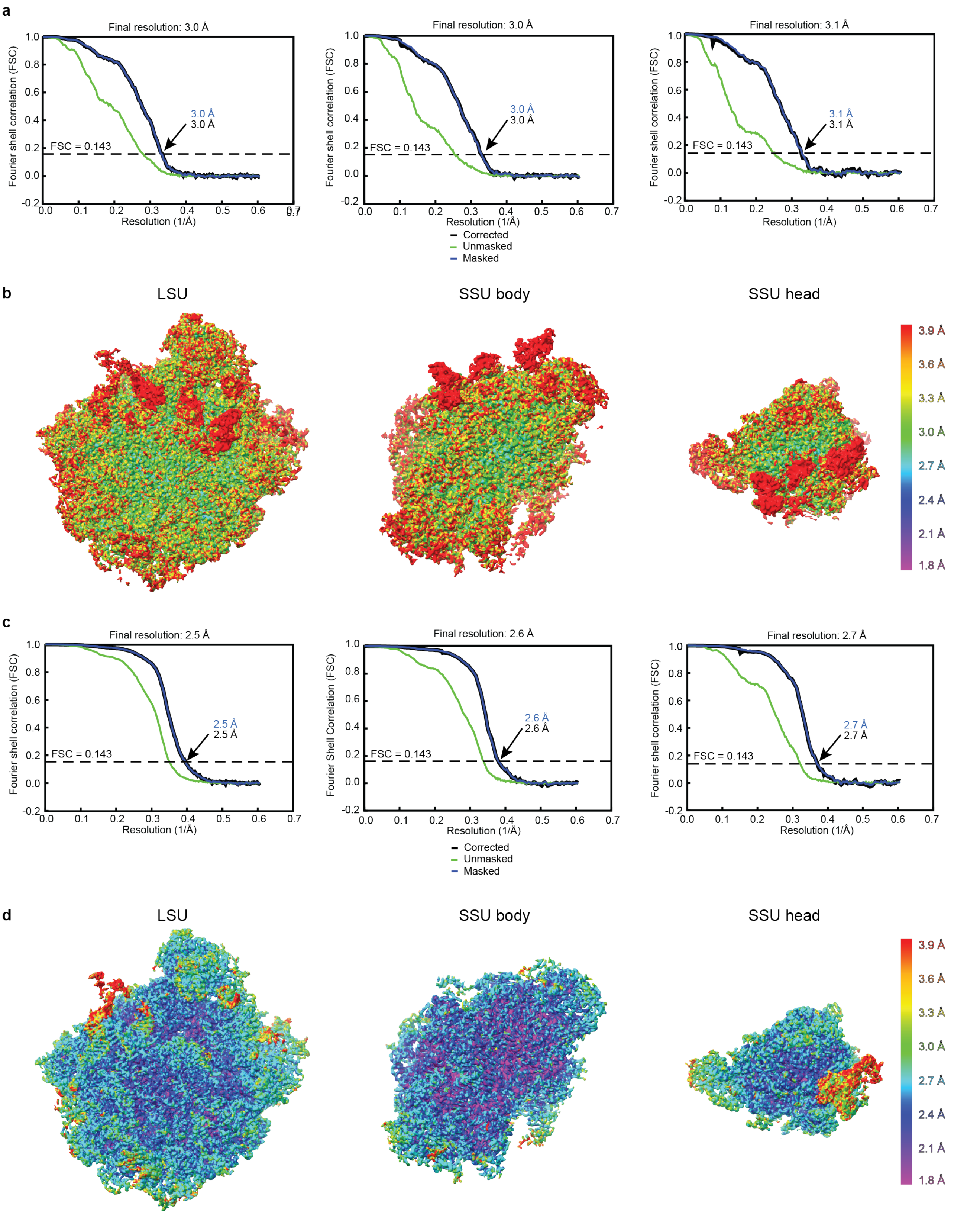

Supplementary Fig. 2 Resolution estimation of ribosome subdomains after multi-body refinement (related to Fig. 1). a 3DC1 Fourier shell correlation (FSC) curves as a function of spatial frequency showing a nominal resolution relative to the gold-standard FSC = 0.143 criterion of 3.0, 3.0, and 3.1 Å for the large subunit (LSU), the body of the small subunit (SSU), and the head of the SSU, respectively. Density is visible for tRNAs occupying the E, P, and A sites. **b** Estimated local resolution map of the interface view of the LSU, SSU body, and of the SSU head, respectively. **c** 3DC3 Fourier shell correlation (FSC) curves as a function of spatial frequency showing a nominal resolution relative to the gold-standard FSC = 0.143 criterion of 2.5, 2.6, and 2.7 Å for the LSU with an E-site tRNA density, the body of the SSU, and the head of the SSU with an E-site density, respectively. **d** Estimated local resolution map of the interface view of the LSU and an E-site tRNA, SSU body, and of the SSU head and E-site tRNA, respectively.

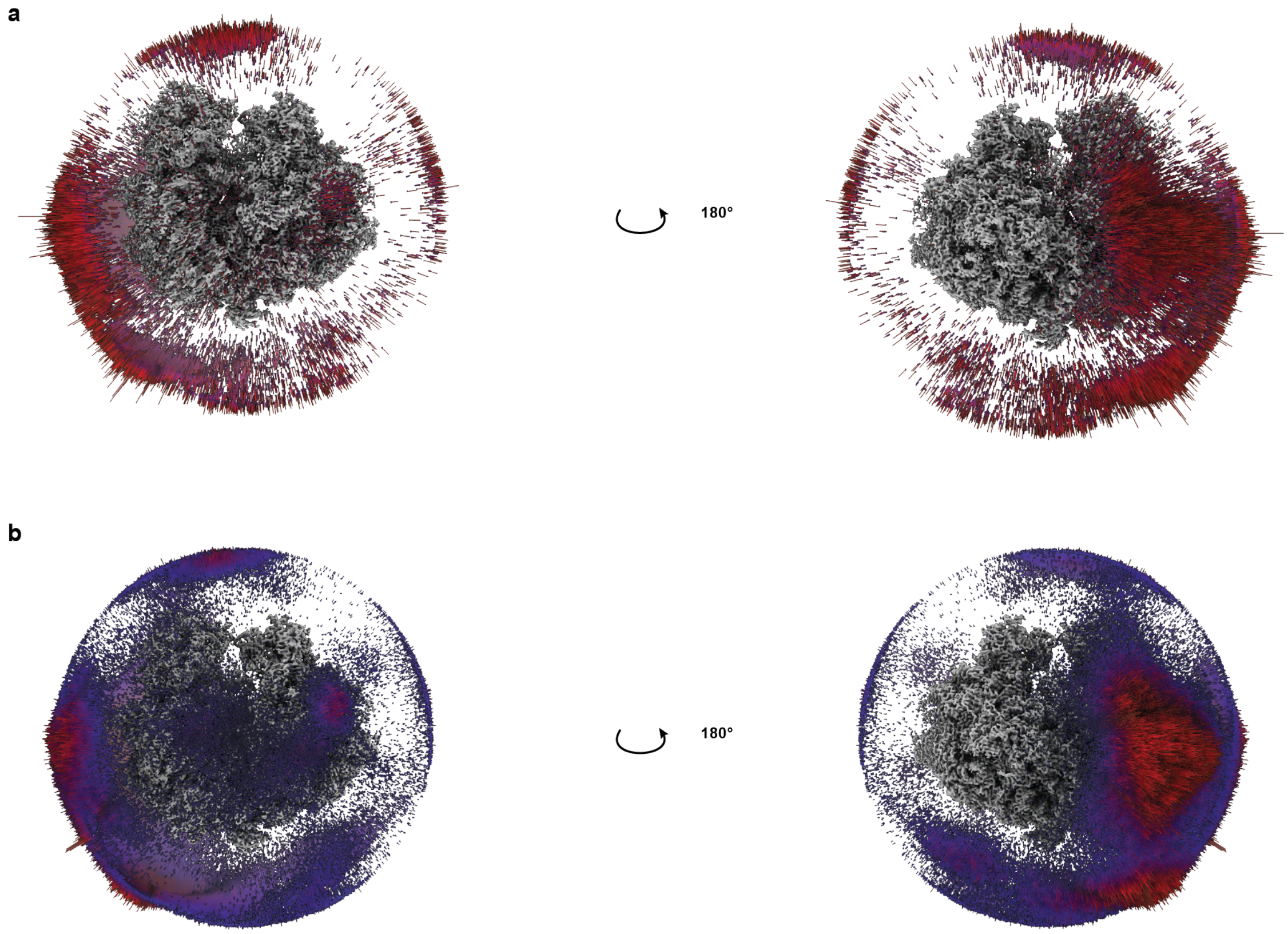

Supplementary Fig.3 3D representation of angular distribution of particles contributing to final reconstructions (related to Fig. 1). Number of views at each angular orientation is represented by cylinder length and color, where red indicates more views and purple less views. The cryo-EM density map is shown in gray at the center. For each class, two views related by a 180° rotation are shown. **a** conformational state 1 (3D Class 1). **b** Conformational state 2 (3D Class 3).

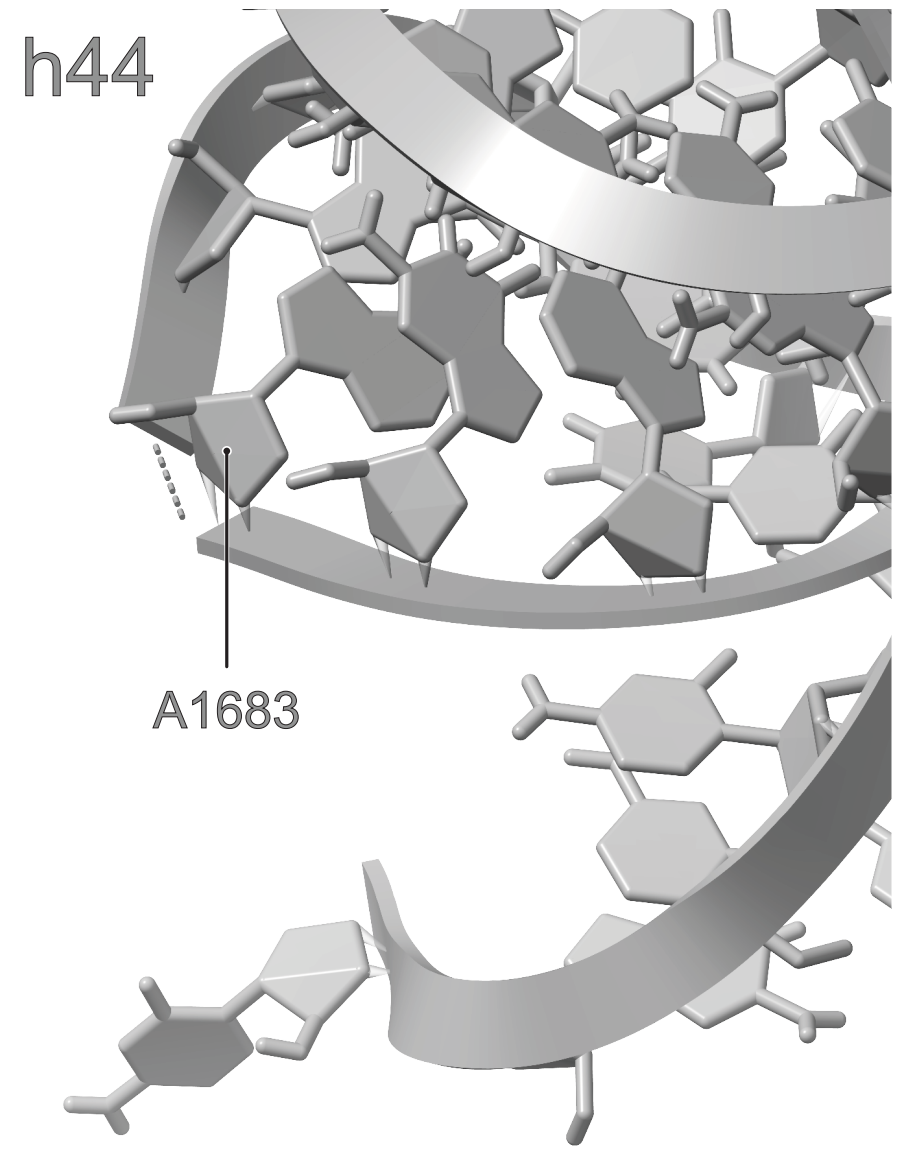

Supplementary Fig. 4 Enlarged view of the decoding center in *B*. *divergens* ribosome containing a single E-site tRNA (conformational state 2, related to Fig. 1). A1683 remains stacked within h44, while A1684 is unresolved and not modeled, consistent with increased flexibility when the A-site is unoccupied.

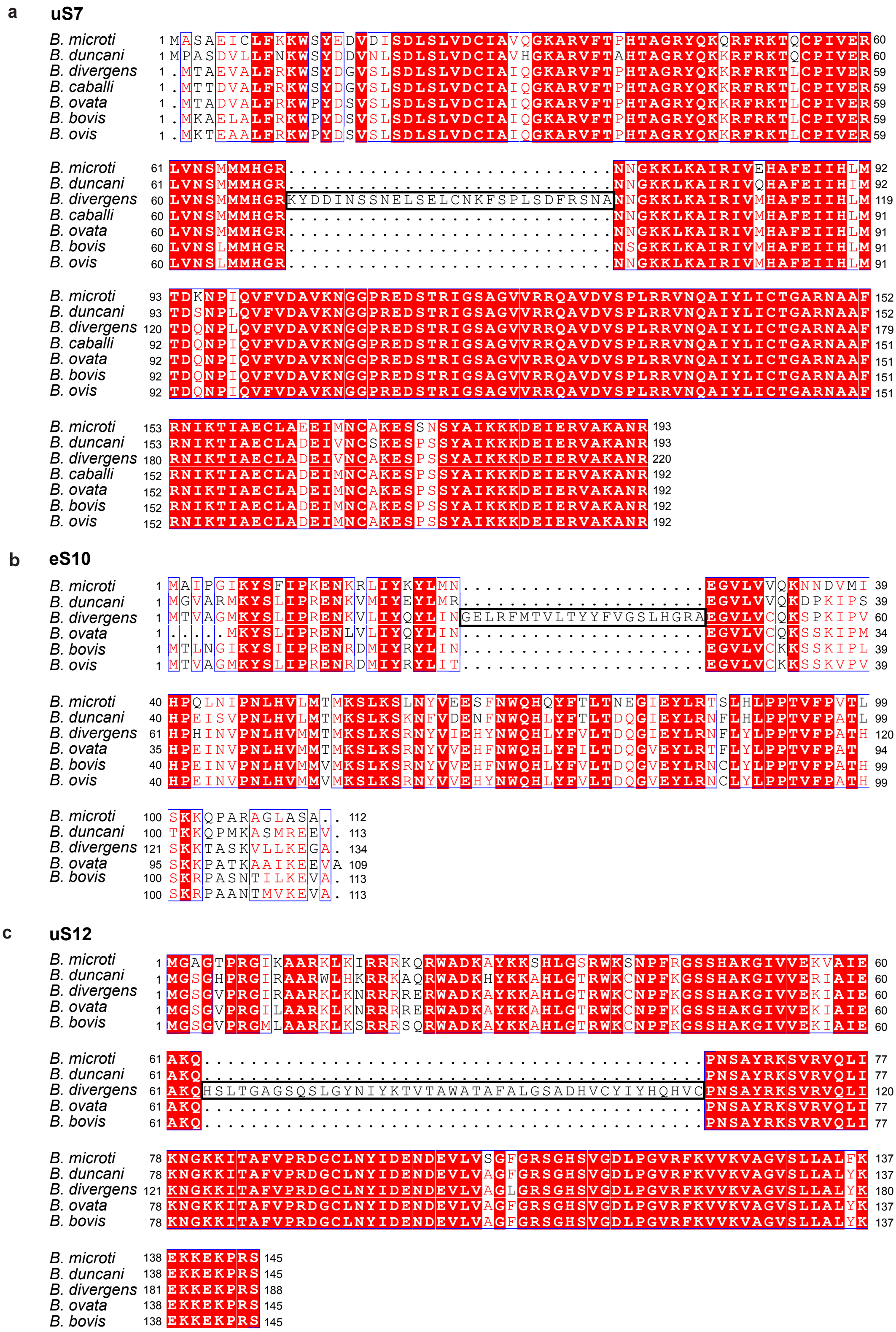

Supplementary Fig. 5 Sequence alignment of ribosomal proteins from the small subunit between *Babesia* species based on genome annotation from PiroplasmaDB (related to Fig. 1). Sequence alignment was generated using Clustal Omega and is displayed using ESPript3. White letters on a red background correspond to identical residues, while red letters on a white background indicate conservation. A black box indicates a portion of a sequence that was misannotated in *B. divergens* 1802A strain, as it overlaps with an intron in *B. divergens* Rouen strain, consistent with the other *Babesia* strains displayed in the alignment. The numbering is based on the *B. microti* sequence. **a** uS7 sequence alignment. PiroplasmaDB gene IDs: *B. microti*: BmR1_04g08092, *B. duncani*: BdWA1_002425, *B. divergens* (1802A): Bdiv_017820c, *B. caballi*: BcabD6B2_13830, *B. ovata*: BOVATA_042040, *B. bovis*: BBOV_I000230, and *B. ovis*:BaOVIS_000820. **b** eS10 sequence alignment. *B. microti*: BmR1_04g06575, *B. duncani*: BdWA1_001572, *B. divergens* (1802A): Bdiv_032170, *B. ovata*: BOVATA_000860, *B. bovis*: BBOV_III009270, and *B. ovis*: BaOVIS_009200. **c** uS12 sequence alignment. *B. microti*: BMR1_02g01585, *B. duncani*: BdWA1_002523, *B. divergens* (1802A): Bdiv_040390c, *B. ovata*: BOVATA_045610, and *B. bovis*:

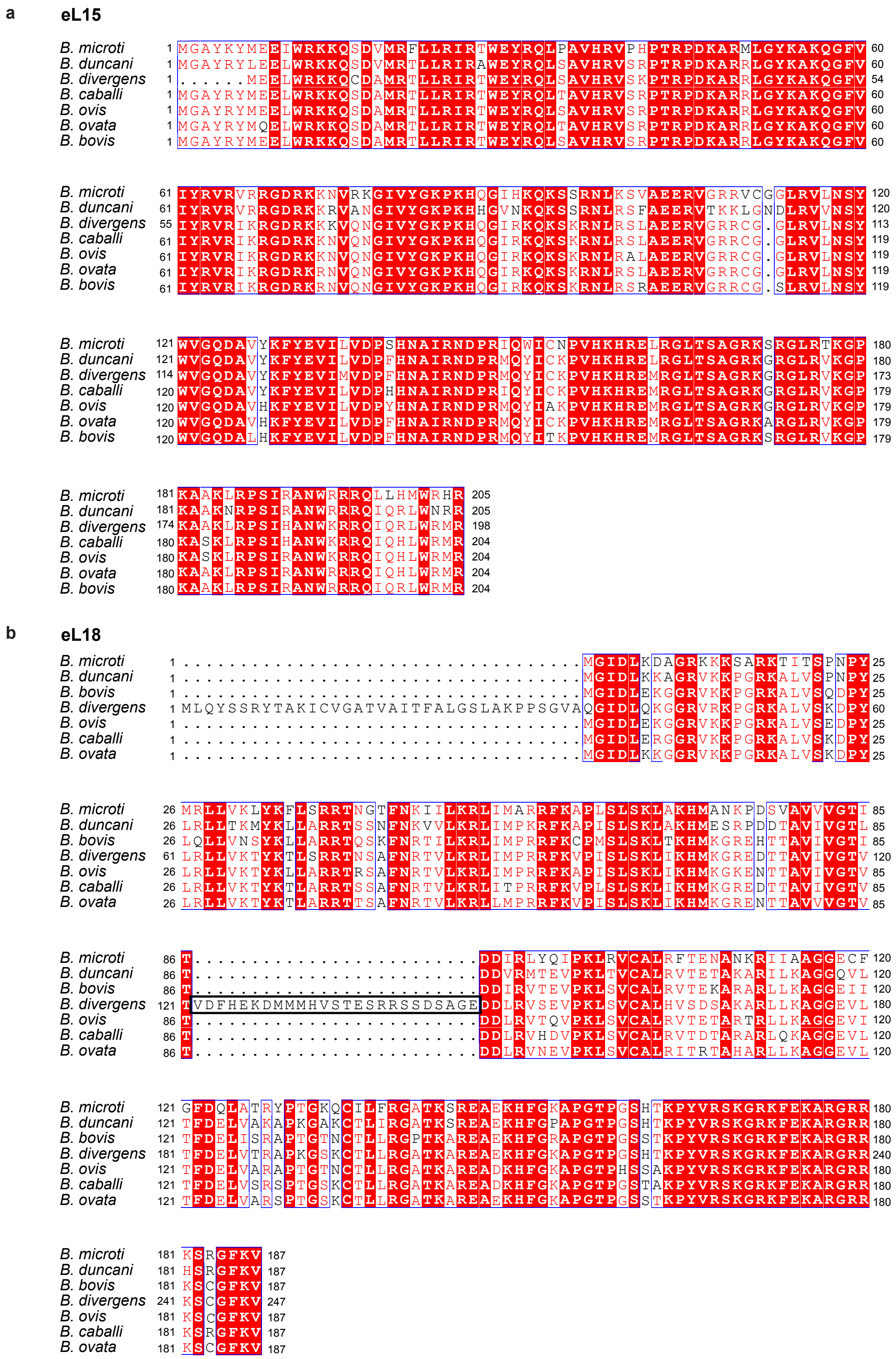
a
eL15
B. microti
B. duncani
B. divergens
B. caballi
B. ovis
B. ovata
B. bovis
b
eL18
B. microti
B. duncani
B. bovis
B. divergens
B. ovis
B. caballi
B. ovata

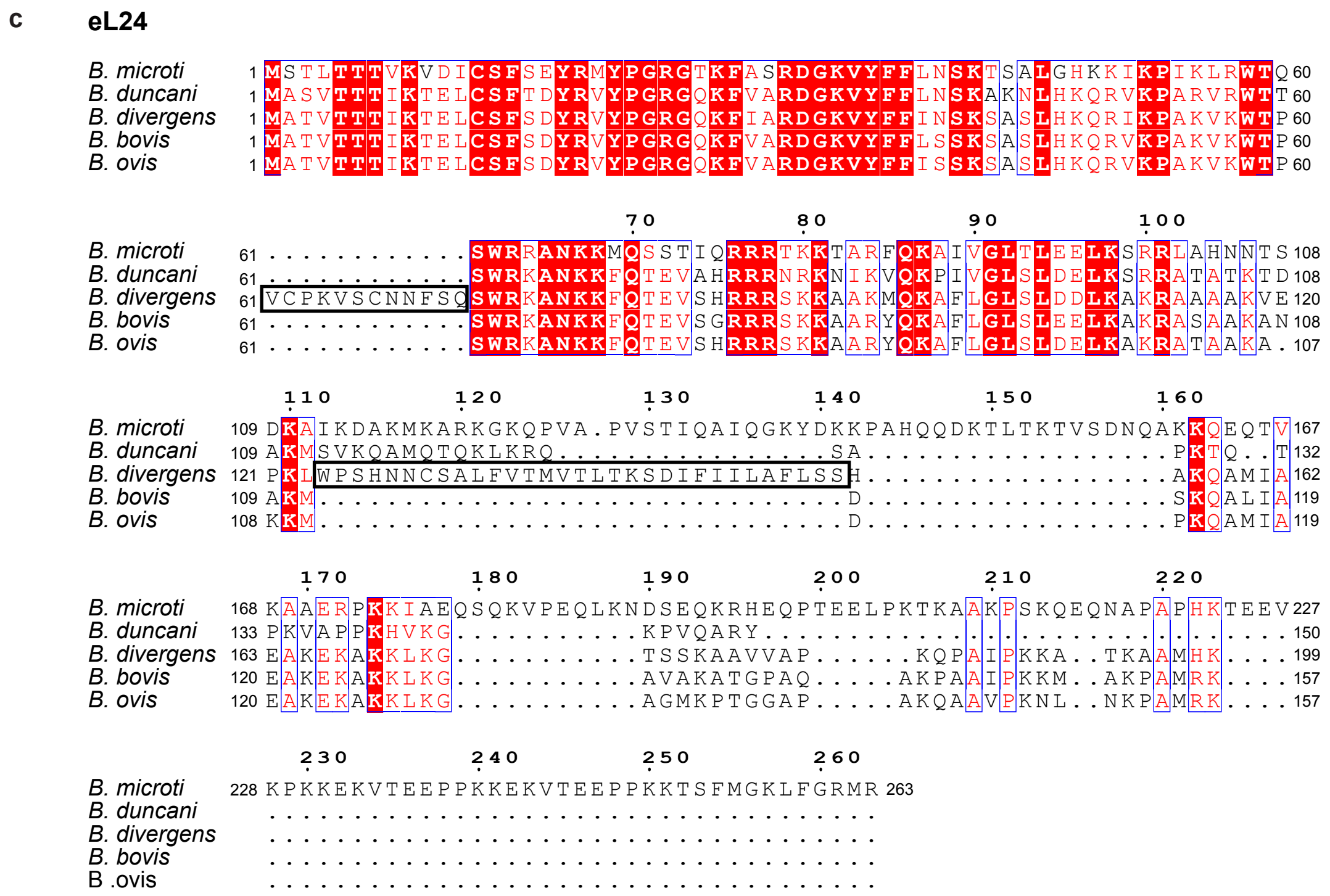

Supplementary Fig. 6 Sequence alignment of ribosomal proteins from the large subunit between *Babesia* species based on genome annotation from PiroplasmaDB (related to Fig. 1). Sequence alignment was generated and displayed as described in Fig. S4. **a** eL15 sequence alignment. Based on cryo-EM density analysis,the eL15 sequence was misannotated and contains the N-terminal sequence (MGAYRY) found in all other *Babesia* species included in the sequence alignment. PiroplasmaDB gene IDs: *B. microti*: BmR1_04g09685, *B. duncani*: BdWA1_002482, *B. divergens* (1802A): Bdiv_018330, *B. caballi*: BcabD6B2_14580, *B. ovis*: BaOVIS_001320, *B. ovata*: BOVATA_042510, and *B. bovis*: BBOV_III000550. **b** eL18 sequence alignment. PiroplasmaDB gene IDs: *B. microti*: BmR1_04g04880, *B. duncani*: BdWA1_000922, *B. divergens* (1802A): Bdiv_037540, *B. caballi*: BcabD6B2_03030, *B. ovata*: BOVATA_006680, *B. bovis*: BBOV_III004640, and *B. ovis*:BaOVIS_004890. **c** eL24 sequence alignment. *B. microti*: BMR1_01G02266, *B. duncani*: BdWA1_002225, *B. divergens* (1802A): Bdiv_036590c, *B. bovis*: BBOV_III003570, and *B. ovis*: BaOVIS_004000.

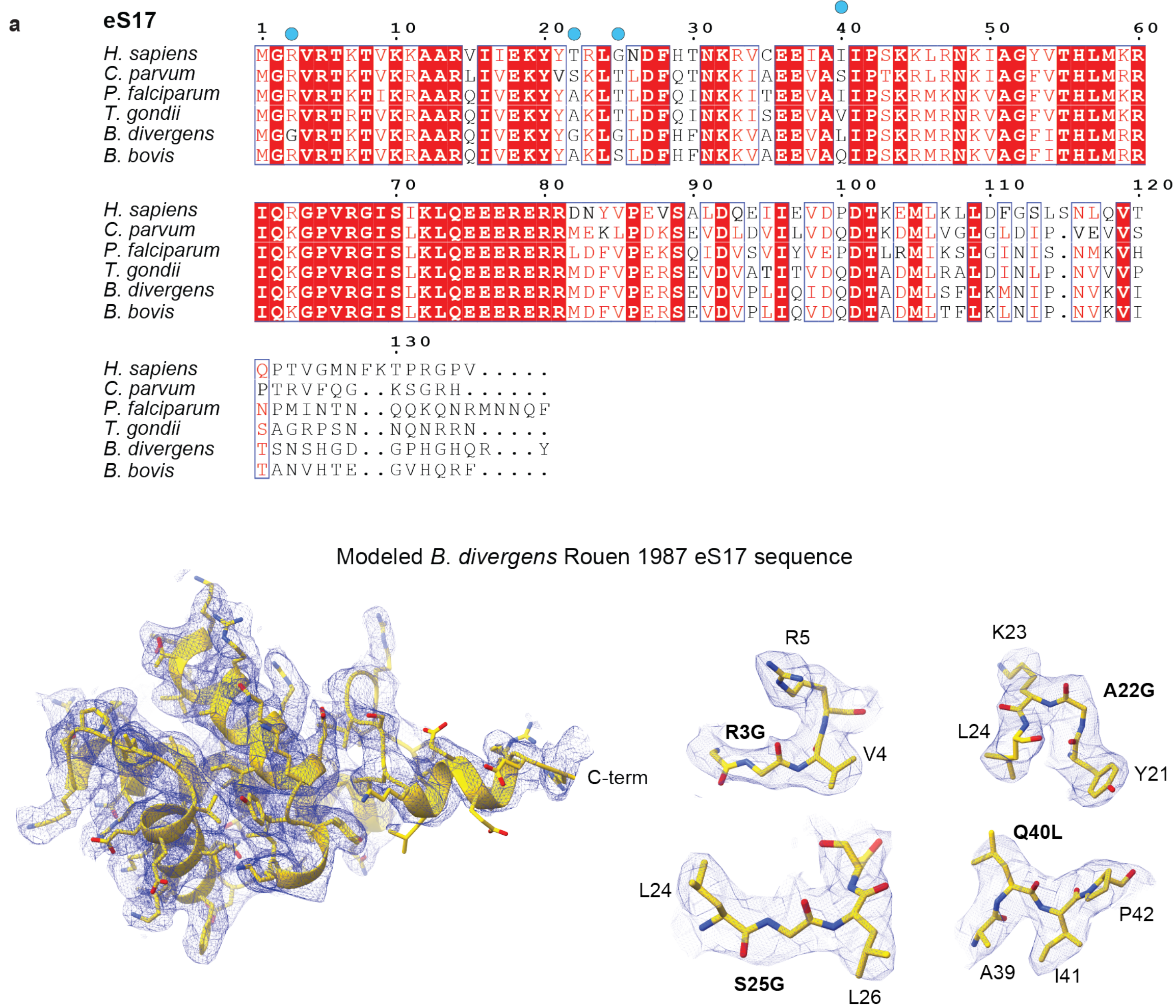

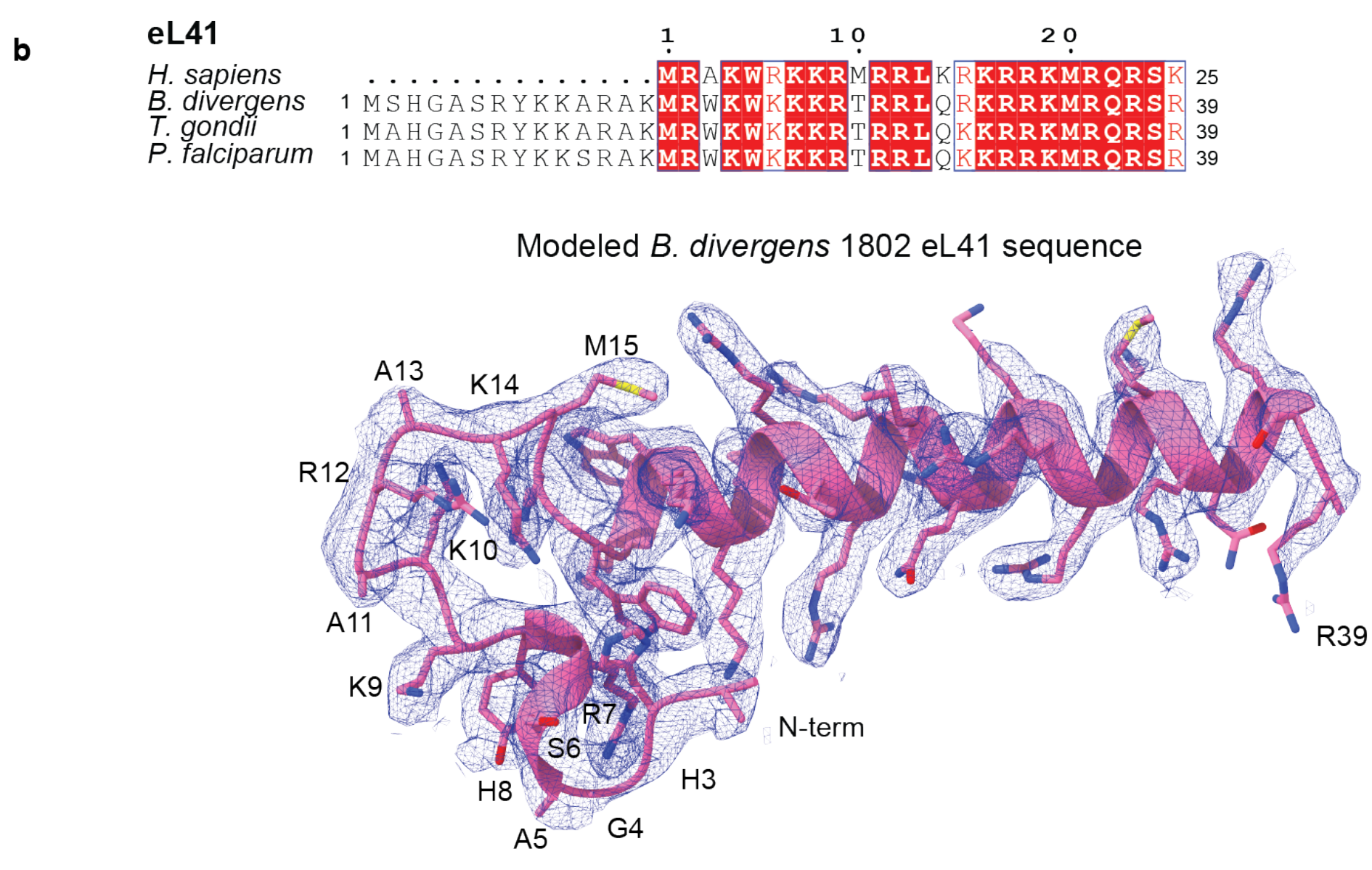

Supplementary Fig. 7 Model fitting of eS17 and eL41 into the *B. divergens* ribosome density (related to Figs. 1 and 3). The sequence alignment was generated and displayed as described in Fig. S4. The numbering is based on the human sequence. **a** The top panel shows the eS17 sequence alignment. Cyan circles indicate residue diffrences between *B. divergens* and *B. bovis*. *H. sapiens*: P08708, *C. parvum*: cgd5_3720, *P. falciparum*: PF3D7_1242700, *T. gondii*: TGME49_20784, *B. divergens* (Rouen 1987): CCSG02000001.1:1890202..1890772 (supercontig), *B. bovis*: BBOV_III001250. The bottom panel shows the atomic model of eS17. eS17 *B. bovis* sequence (BBOV_III001250) used for initial model building (Alphafold2) and further mutated over four residues (R3G, A22G, S25G, Q40L) to match the corresponding *B. divergens* protein sequence identified from the Rouen 1987 strain. The left panel displays the complete eS17 model (contour level of 5.0), while the right panel highlights the mutated residues from *B. bovis* to *B. divergens* (contour level of 6.0). **b** The top panel shows the eL41 sequence alignment. eL41 gene IDs. *H. sapiens* : P62945c, *B. divergens* (1802): JAHBMH010000034: 30674..30988 supercontig, *T. gondii*: TGME49_213580, and *P. falciparum*: PF3D7_1144300. The bottom panel shows the atomic model of eL41 in the density map (contour level of 5.0), including the N-terminal extension (residues 2–14) found in apicomplexan parasites.

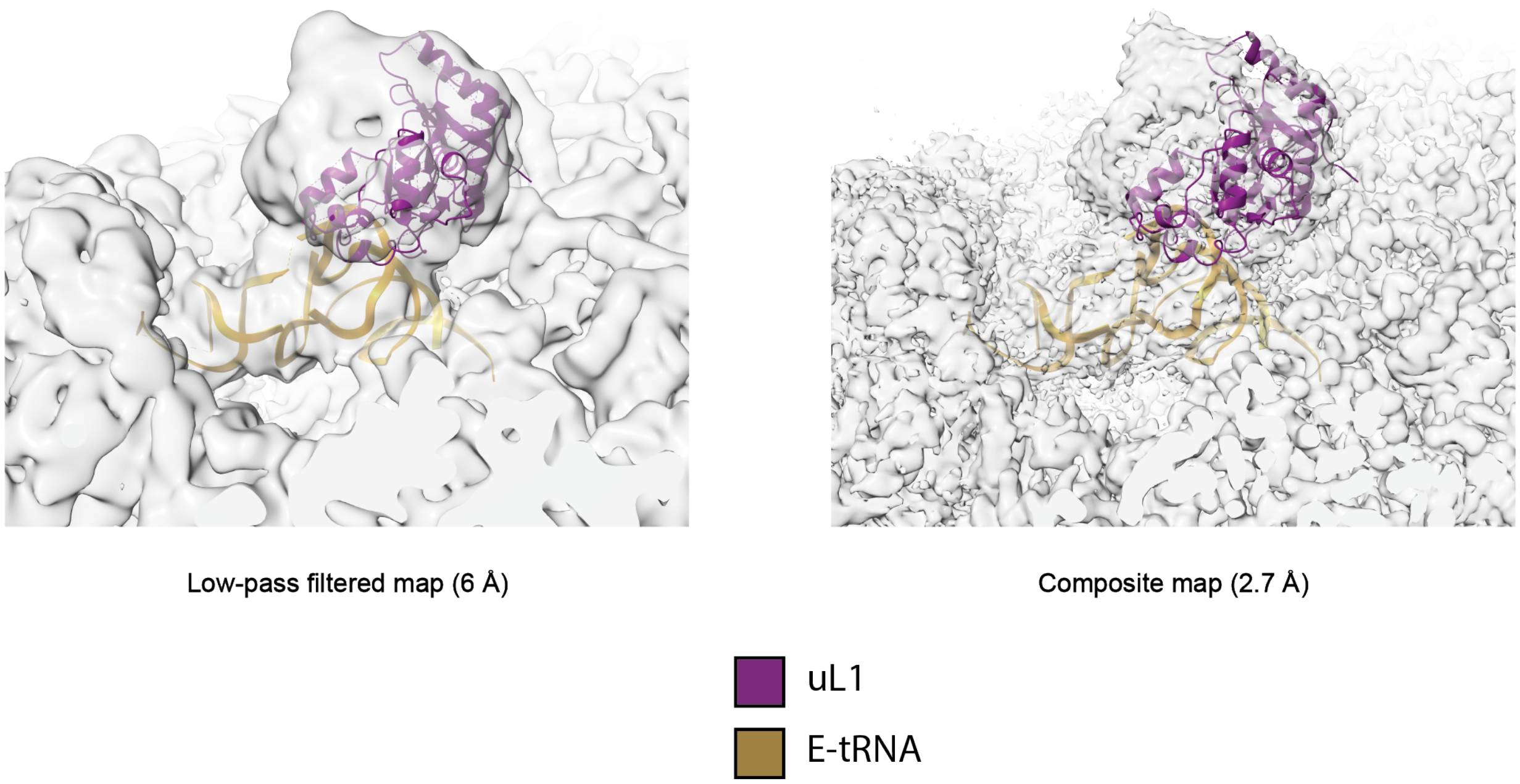

Supplementary Fig. 8 Transparent surface representations of the low-pass filtered map to 6 Å (left) and the corresponding composite multibody refinement map (right), contoured at sdLevel 2 (2σ) showing the L1 stalk (uL1) region (related to Fig. 1). The ribosomal protein uL1 and E-site tRNA are shown as ribbon representations in purple and orange, respectively. While the low-pass filtered map aided initial *B. divergens* uL1 docking, only partial density remained visible at higher contour levels (>3σ).

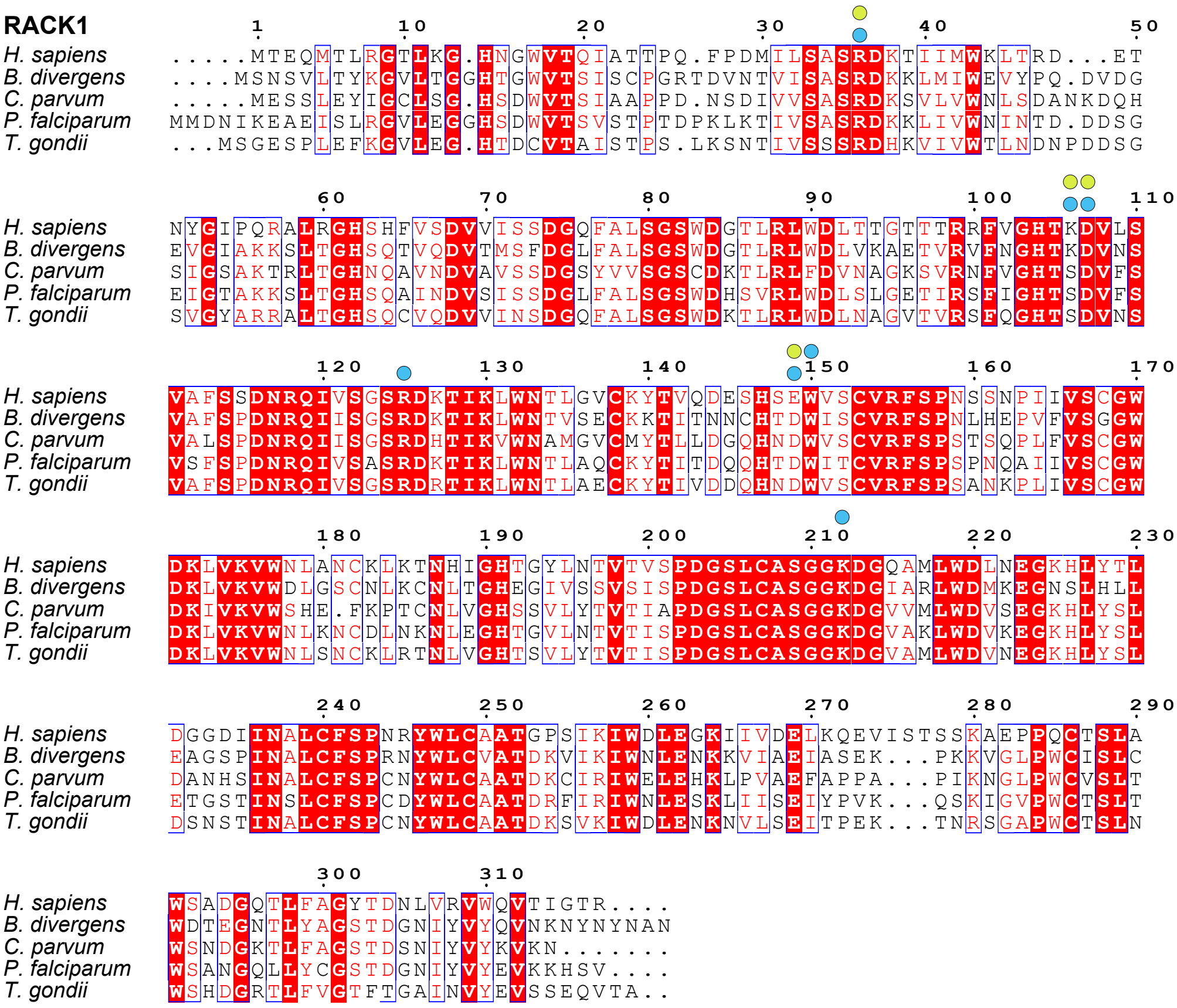

Supplementary Fig. 9 Sequence alignment of RACK1 (related to Figs. 1 and 3). The sequence alignment was generated and displayed as described in Fig. S3. The numbering is based on the human sequence. Cyan and green circles indicate RACK1 residues interacting with eS17 through hydrogen bonds or non-covalent interactions in *B. divergens* and humans, respectively. VEuPathDB gene IDs: *H. sapiens*: P63244, *B. divergens*: Bdiv_010290, *C. parvum*: cgd2_1870, *P. falciparum*: PF3D7_0826700, and *T. gondii*: TGME49_216880.

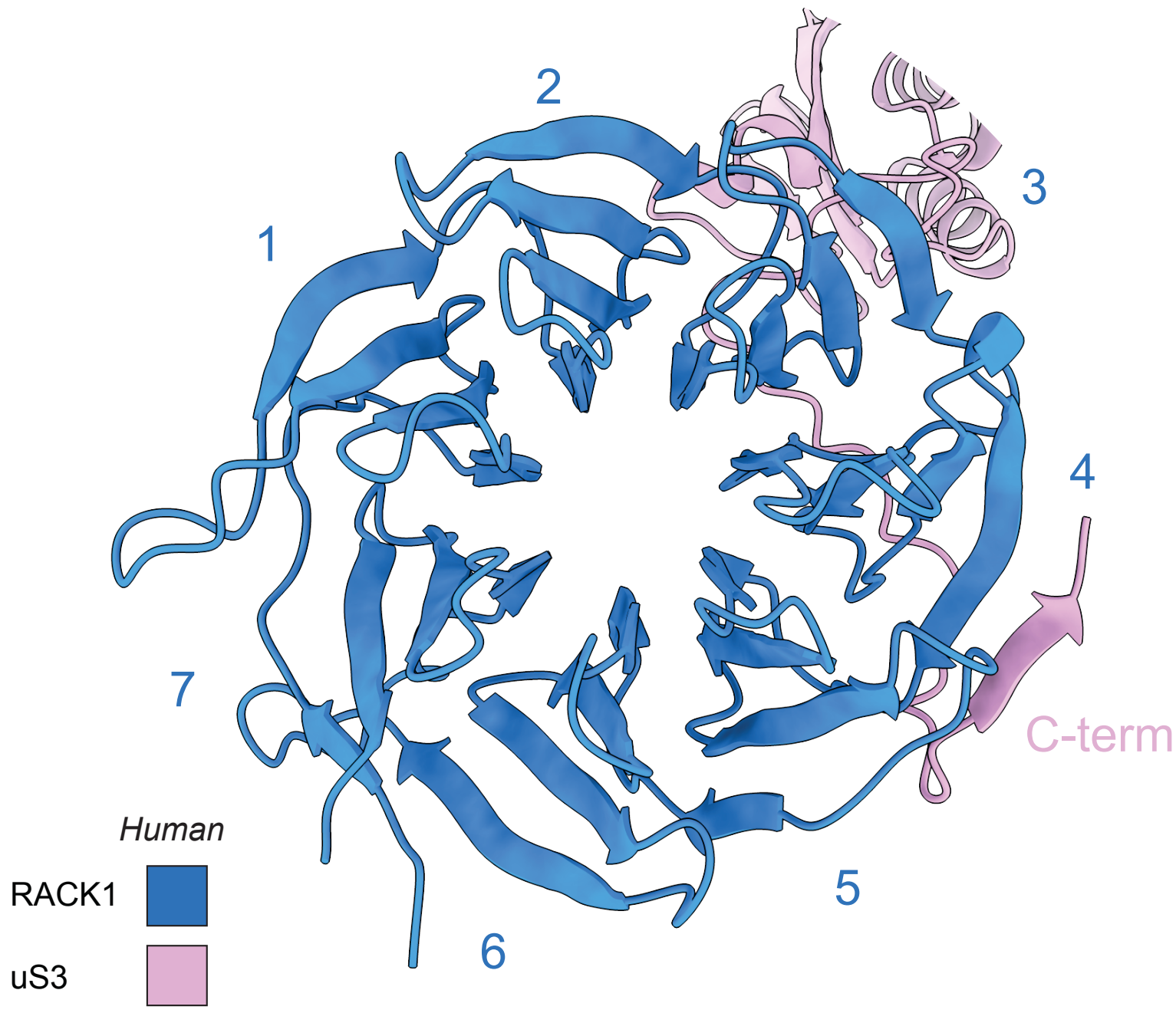

Supplementary Fig. 10 Overview of the localization of the human C-terminal tail of uS3 relative to human RACK1 within the head of the SSU (PDB 8QOI, related to Fig. 3). View from the top of RACK1, showing each propeller blade (1-6, blue). The C-terminal (C-term) tail of uS3 (pink) interacts with RACK1 between the propeller
