# Supplementary material for: Ribosomal architecture and rRNA modification landscape in the tick-borne parasite Babesia divergens
Source: Nat Commun. 2026 Aug 6;17:9475. doi: 10.1038/s41467-026-75282-4 (PMC13542104; doi:10.1038/s41467-026-75282-4)
Supplement: Supplementary file 2 [file 41467_2026_75282_MOESM2_ESM.pdf]

nature portfolio

Corresponding author(s): Melissa Leger-Abraham

Last updated by author(s): 06/18/2026

Reporting Summary

Nature Portfolio wishes to improve the reproducibility of the work that we publish. This form provides structure for consistency and transparency in reporting. For further information on Nature Portfolio policies, see our Editorial Policies and the Editorial Policy Checklist.

Statistics

For all statistical analyses, confirm that the following items are present in the figure legend, table legend, main text, or Methods section.

n/a	Confirmed	
☐	☒	The exact sample size (*n*) for each experimental group/condition, given as a discrete number and unit of measurement
☐	☒	A statement on whether measurements were taken from distinct samples or whether the same sample was measured repeatedly
☒	☐	The statistical test(s) used AND whether they are one- or two-sided<br>*Only common tests should be described solely by name; describe more complex techniques in the Methods section.*
☒	☐	A description of all covariates tested
☐	☒	A description of any assumptions or corrections, such as tests of normality and adjustment for multiple comparisons
☒	☐	A full description of the statistical parameters including central tendency (e.g. means) or other basic estimates (e.g. regression coefficient) AND variation (e.g. standard deviation) or associated estimates of uncertainty (e.g. confidence intervals)
☒	☐	For null hypothesis testing, the test statistic (e.g. *F*, *t*, *r*) with confidence intervals, effect sizes, degrees of freedom and *P* value noted<br>*Give P values as exact values whenever suitable.*
☒	☐	For Bayesian analysis, information on the choice of priors and Markov chain Monte Carlo settings
☒	☐	For hierarchical and complex designs, identification of the appropriate level for tests and full reporting of outcomes
☒	☐	Estimates of effect sizes (e.g. Cohen's *d*, Pearson's *r*), indicating how they were calculated

Our web collection on statistics for biologists contains articles on many of the points above.

Software and code

Policy information about availability of computer code

Data collection	FEI Titan Krios electron microscope, Serial EM for automated data acquisition (cryo-EM), Lumos Tribrid Orbitrap Mass Spectrometer equipped with Ultimate 3000 (Thermo Fisher) nano-HPLC, QuBit, TapeStation, PromethION flow cell (FLO-PRO004RA) (nanopore sequencing)
Data analysis	MotionCor2 (gain correction, alignment and dose-weight movies), CTFFIND-4.1.13 (aligned micrographs for CTF), crYOLO (particle picking), RELION 3.1.2 and RELION 5.0 (2D, 3 classifications), ChimeraX (maps processing and docking), Coot0.9.8.95 (model building), PHENIX1.21.2-5419 (refinement)<br>Proteome Discoverer 2.4 software (mass spectrometry raw data analysis)<br>MinKNOW v24.06.15 (nanopore sequencing basecalling)<br>Oxford Nanopore's basecaller Dorado 0.9.0: rna004_130pbs_sup@v5.1.0, pseudouridines)<br>Dorado v1.0.2 (rna004_130bps_sup@v5.2.0, for methylation)<br>SAMtools (reads alignement)<br>Modkit 0.5.0 (rRNA modification sites identification)

For manuscripts utilizing custom algorithms or software that are central to the research but not yet described in published literature, software must be made available to editors and reviewers. We strongly encourage code deposition in a community repository (e.g. GitHub). See the Nature Portfolio guidelines for submitting code & software for further information.

Data

Policy information about availability of data

All manuscripts must include a data availability statement. This statement should provide the following information, where applicable:

- Accession codes, unique identifiers, or web links for publicly available datasets
- A description of any restrictions on data availability
- For clinical datasets or third party data, please ensure that the statement adheres to our policy

The atomic coordinates and cryo-EM maps generated in this study have been deposited in the PDB and Electron Microscopy Data Bank (EMDB), respectively. For the conformational state 1 (3DC1, 80S ribosome with three tRNAs and mRNA), the PDB accession code is 9YXB [https://www.rcsb.org/structure/9YXB] with the following associated EMDB entries: EMD-73602 (composite: [https://www.ebi.ac.uk/emdb/EMD-73602]), EMD-73574 (consensus: [https://www.ebi.ac.uk/emdb/EMD-7373574]), EMD-73576 (LSU: [https://www.ebi.ac.uk/emdb/EMD-7373576]), EMD-73577 (SSU body: [https://www.ebi.ac.uk/emdb/EMD-73577]), EMD-73581 (SSU head: [https://www.ebi.ac.uk/emdb/EMD-73581]). For the conformational state 2 (3DC3, 80S ribosome with E-site tRNA), the PDB accession code is 9YGM [https://www.rcsb.org/structure/9YGM] with the following associated EMDB entries: EMD-72933 (composite: [https://www.ebi.ac.uk/emdb/EMD-72933]), EMD-72052 (consensus: [https://www.ebi.ac.uk/emdb/EMD-72052]), EMD-72055 (LSU: [https://www.ebi.ac.uk/emdb/EMD-72055]), EMD-72069 (SSU body: [https://www.ebi.ac.uk/emdb/EMD-72069]), EMD-72070 (SSU head: [https://www.ebi.ac.uk/emdb/EMD-72070]). The mass spectrometry proteomics data have been deposited to the ProteomeXchange Consortium via the PRIDE partner repository with the dataset identifier PXD069329 [https://proteomecentral.proteomexchange.org/?pxid=PXD069329]. Nanopore sequencing datasets have been deposited in NCBI Sequence Read Archive (SRA) under BioProject ID PRJNA1358264 [https://www.ncbi.nlm.nih.gov/bioproject/PRJNA1358264] and SRA ID SAMN53107339 [https://www.ncbi.nlm.nih.gov/sra/?term=SAMN53107339]. All other materials are available upon request and should be addressed to MLA.

Research involving human participants, their data, or biological material

Policy information about studies with human participants or human data. See also policy information about sex, gender (identity/presentation), and sexual orientation and race, ethnicity and racism.

Reporting on sex and gender	N/A
Reporting on race, ethnicity, or other socially relevant groupings	N/A
Population characteristics	N/A
Recruitment	N/A
Ethics oversight	N/A

Note that full information on the approval of the study protocol must also be provided in the manuscript.

Field-specific reporting

Please select the one below that is the best fit for your research. If you are not sure, read the appropriate sections before making your selection.

☒ Life sciences ☐ Behavioural & social sciences ☐ Ecological, evolutionary & environmental sciences

For a reference copy of the document with all sections, see nature.com/documents/nr-reporting-summary-flat.pdf

Life sciences study design

All studies must disclose on these points even when the disclosure is negative.

Sample size	Mass spectrometry analysis was conducted on one ribosome purification sample<br>Nanopore sequencing analysis resulted from one biological sample.<br>These sample size were chosen based on material availability (limited parasite ribosome purification) and cost of experiments (e.g., nanopore sequencing).
Data exclusions	Some micrographs were excluded during data processing, where 80.9% of all particles picked were use to obtain 3D classes. Also, in the rRNA modification analysis, we used specific threshold (30-30 %, and over 50%) throughout our analysis which is described thoroughly in the Methods section of the manuscript.
Replication	Mass spectrometry analysis was done once<br>Nanopore sequencing resulted from two independent runs (technical replication)
Randomization	N/A to cryo-EM study
Blinding	N/A to cryo-EM study

Reporting for specific materials, systems and methods

We require information from authors about some types of materials, experimental systems and methods used in many studies. Here, indicate whether each material, system or method listed is relevant to your study. If you are not sure if a list item applies to your research, read the appropriate section before selecting a response.

Materials & experimental systems

n/a	Involved in the study
☒	☐ Antibodies
☐	☒ Eukaryotic cell lines
☒	☐ Palaeontology and archaeology
☒	☐ Animals and other organisms
☒	☐ Clinical data
☒	☐ Dual use research of concern
☒	☐ Plants

Methods

n/a	Involved in the study
☒	☐ ChIP-seq
☒	☐ Flow cytometry
☒	☐ MRI-based neuroimaging

Eukaryotic cell lines

Policy information about cell lines and Sex and Gender in Research

Cell line source(s)	Babesia divergens Rouen 1987, red blood cells
Authentication	B. divergens Rouen strain was originally obtained from Kirk Deitsch/Laura Kirkman. Caucasian male O-positive human red blood cells are from Research Blood Components.
Mycoplasma contamination	N/A
Commonly misidentified lines (See ICLAC register)	*Name any commonly misidentified cell lines used in the study and provide a rationale for their use.*

Plants

Seed stocks	*Report on the source of all seed stocks or other plant material used. If applicable, state the seed stock centre and catalogue number. If plant specimens were collected from the field, describe the collection location, date and sampling procedures.*
Novel plant genotypes	*Describe the methods by which all novel plant genotypes were produced. This includes those generated by transgenic approaches, gene editing, chemical/radiation-based mutagenesis and hybridization. For transgenic lines, describe the transformation method, the number of independent lines analyzed and the generation upon which experiments were performed. For gene-edited lines, describe the editor used, the endogenous sequence targeted for editing, the targeting guide RNA sequence (if applicable) and how the editor was applied.*
Authentication	*Describe any authentication procedures for each seed stock used or novel genotype generated. Describe any experiments used to assess the effect of a mutation and, where applicable, how potential secondary effects (e.g. second site T-DNA insertions, mosiacism, off-target gene editing) were examined.*
