# Supplementary material for: Ribosomal architecture and rRNA modification landscape in the tick-borne parasite Babesia divergens
Source: Nat Commun. 2026 Aug 6;17:9475. doi: 10.1038/s41467-026-75282-4 (PMC13542104; doi:10.1038/s41467-026-75282-4)
Supplement: Supplementary file 3 [file 41467_2026_75282_MOESM3_ESM.pdf]

nature portfolio

Peer Review File

Ribosomal Architecture and rRNA Modification Landscape in the Tick-Borne Parasite *Babesia divergens*

Corresponding Author: Dr Mélissa Léger-Abraham

This file contains all reviewer reports in order by version, followed by all author rebuttals in order by version.

Version 0:

Reviewer comments:

Reviewer #1

(Remarks to the Author)
The manuscript by Gutierrez-Vargas et al. reports on the structural analysis of a peculiar eukaryotic ribosome that was isolated from a tick-borne parasite. The study focuses on the structural aspects and hints at functional implications of the specific structural features of this ribosome such as the rRNA and ribosomal proteins (e.g. eL28 appears to be specifically absent, RACK1 is well visible) and chemical modifications of the rRNA (cross-validated by nanopore analysis). Two cryo-EM structures were obtained at 3.1 and 2.6 Å resolution with either 3 tRNAs or only E-site tRNA bound, respectively. The study gives very interesting novel insights into the specific nature of a ribosome from a pathogenic species. The manuscript is overall well written but some aspects could be clarified as detailed below.

Detailed points:
- abstract and text: the therapeutic applications are certainly interesting per se, but maybe these need not be emphasized too much at this stage as direct conclusions on drug design are not drawn here yet. These are just new findings, which are interesting by themselves.
- abstract and text: implications in gene expression is a general statement that applies to other species also, it could be clarified what is specific about it here.
- abstract: no need to repeat “tick-borne apicomplexan pathogen”
- “atomic resolution”: even though the resolution of 2.6 Å is very good, in crystallography people would still argue that this is not atomic because individual atoms are not resolved (which would a resolution closer to 1 Å resolution).
- resolution estimation: the estimated overall resolution is indicated with 2.6 Å, but the estimation from map-model FSC is 2.74 Å, which may indicate some overestimation. What is the d99 value that the Phenix software provide? Also, the average resolution of 2.6-2.7 Å indicates that it is unlikely to have data extending to 1.7 Å. The features in the map do not support that really. The conclusions drawn on the structure appear solid, so there is no need to overstate resolution.
- introduction: explain apicomplexan
- it is interesting to see that some new modification sites are being proposed. However, some of these are only found according to the nanopore sequencing data, but not visible in the structure. How reliable are these? Until recently nanopore analysis of chemical modifications was considered complementary for validation, but not very precise by itself as a standalone technique. Is there independent evidence from MS, RiboMethSeq or other techniques for the new sites? Otherwise it might be better to list these sites apart or label them differently.
- methylation sites: the description in the results and discussion discusses 2’-O-methylations and nucleotide base methylations simultaneously, which is a bit confusing. It would be easier to follow if discussed separately. These are distinct types of modifications, also implying a different chemical reactivity and hydrogen-bonding properties in the structure.
line 104: morphology of 80S ribosomes: compared to which structure / organism?
line 109: human 1824/1825: reference? e.g. PMID: 25901680
line 113: data not shown, but the stacking inside helix h44 could be interesting to show
- cryo-EM data processing and usage of local/focused classifications and refinements and multibody refinement strategies: some references could be useful here; e.g. PMID: 14985767 (original concept in the field); PMID: 27572726 (review); PMID: 37659578 (recent paper & refs therein); PMID: 29856314 (multibody refinement)
- line 122: this is local resolution
- Fig. 1c, right: it seems that the 40S ribosomal subunit head could be refined further; apparently, focused refinement and multibody refinement were applied, but was this with partial signal subtraction and recentering of the particle images?
- Fig. 2 legend: “threshold” should better say “contour” level as no cut of the data is done when displaying maps; to be

checked throughout
- pseudo-uridine N1 coordination by a water molecule: this has been seen in other ribosome structures as well; maybe mention some. Are these exactly in plane with the nucleotide base? They should normally be.
- same for Zn2+ and Mg2+ ions, tetrahedral and octahedral have been described in detail for some other ribosome structures including human (PMID: 38844527); it seems that the concept of that described in lines 136-138 is taken from there.
- lines 150/151: not sure to understand this phrase but it seems obvious that introns are not present anymore in the maturated mRNA coding for the corresponding proteins. Does it imply that the genome annotations are incomplete? Interesting though to see it validated here from the protein sequence in the structure.
- L1 and P stalk are not visible, but usually in the presence of E-site tRNA (state 2) the L1 stalk is stabilized (e.g. in PMID: 27665925 where we discussed this). Maybe filtering the map to ~6 Å resolution could help visualizing the L1 stalk if it is less ordered. Is the E-site tRNA fully defined structurally on both 40S and 60S sides?
- line 176: uS3 in the human ribosome structure: mention which structure you refer to (probably PMID: 25901680). Same for lines 183 and 188. It is interesting to see the comparison with human ribosome structure that is being discussed, also because this may provide hints at how to generate species-specific drugs. Same regarding comparisons of chemical modifications (this would be PMID: 38844527).
- lines 192 etc.: the explanation for the apparent differences in RACK1 sequences might be fine, but maybe sample purification and image processing / structure sorting have led to identify the most stable protein species?
- rRNA expansion segments (ESs): interesting aspect; line 206: human ESs: which reference?
- annotation of ES elements: usually these carry the extra letter L or S to specify to which ribosomal subunit they belong to; e.g. ES12S instead of ES12 etc. (line 210 and possibly elsewhere)
- it is interesting to see that ribosomal protein eS28 is absent; is this possibly due to an expansion of ES10L (Fig. 4b)? or some other relationship between ES differences and eS28?
- line 262: specify which methylation type is being discussed
- line 263-265: if there are about 30 new methylation site and only a quarter of those visible, are the other 3/4 just not resolved possibly? The description here could benefit from discussing 2'-O-methylations and nucleotide base methylations separately
- Fig. 5c: the parallel lines are a bit confusing, a star-like labeling would be easier + less overlapping with the structure behind
- Figs 5 & 6a: there seems to be many new suggested sites; see comment above, as there are still relatively many not visible in the structure are there other data (RiboMethSeq) to confirm this independently? If not, it is not a problem as this is a new species being analysed, but it may be good to clarify in how far nanopore sequencing alone is safe to annotate new sites. Could there be any false positives by nanopore sequencing? Any data available from other parasite rRNA?
- line 273: specify which methylation type
- line 278: characteristic hydrogen bonding on the N1 position: indeed, as seen in the human ribosome structure that is being compared with afterwards (PMID: 38844527); the new PSU sites are interesting indeed.
- line 279: ref 64 is an MS-based analysis, ref 43 used MS too; there is also HydraPsiSeq etc.
- discussion: L1 and P stalks: see comment above. That the P stalk is not visible is not surprising, but the L1 stalk would be expected to be better ordered in the E-site tRNA complex.
- line 325: RACK1 in yeast: was this not also seen in the yeast 80S ribosome crystal structure (PMID: 22096102)? It is also present in the human ribosome structure (PMID: 25901680)
- outlook: divergent rRNA modifications: rather than mentioning the concept alone maybe cite some / detail more?
- methods: Relion etc.: the software Relion etc.
- lines 426-439: the procedure is good, one could include some refs for focused classification and refinement and multibody refinement
- nanopore sequencing (line 533 etc.): the procedure seems to use some new features, which could be explained more; are there any scripts etc. that could be provided to the readers to help reproducibility etc.?
- references to resolution estimation from Fourier shell correlation (FSC) typically comprise 1) van Heel M., Keegstra W., Schutter W. G. & van Bruggen E. F. J. Arthropod hemocyanin studied by image analysis. Life Chem Rep Suppl 69–73 (1982). 2) Saxton, W. O. & Baumeister, W. The correlation averaging of a regularly arranged bacterial cell envelope protein. J. Microsc. 127, 127–138 (1982). 3) Rosenthal, P. B. & Henderson, R. Optimal determination of particle orientation, absolute hand, and contrast loss in single-particle electron cryomicroscopy. J. Mol. Biol. 333, 721–745 (2003).
- validation report for the refined coordinates: there are quite some close contacts; it could help to refine the atomic model with the Phenix software including hydrogen atoms, which help removing clashes due to the van der Waals contacts being taken into account better. Those are not visible but help refining (as riding atoms with predefined geometry) and do not need to be included in the deposited model.
- generic point: cryo-EM maps, half maps, masks etc. and fully refined atomic models should be deposited in the EMDB and PDB; deposition of representative data onto the EMPIAR data base could be considered also.
Thank you for the opportunity to review this work.
Bruno Klaholz

Reviewer #2

(Remarks to the Author)
I would highly recommend publishing the manuscript “Ribosomal Architecture and rRNA Modification Landscape in the Tick-Borne Parasite Babesia divergens” by Gutierrez Vargas et al. with the additional revisions mentioned below:
Apicomplexans, obligate intracellular parasites, have highly reduced genomes, a complex life cycle that involves multiple hosts, and specialized cellular structures that reflect their parasitic lifestyle. The structure of apicomplexan ribosomes presents a remarkable example of cellular adaptation to intracellular parasitism.

In this manuscript, the structure of the Babesia divergence ribosome is reported as the first one from Babesia spp. And one of the few in the realm of apicomplexan. The CryoEM structure of Babesia divergence is 2.6 A resolution, with 1.7 A resolution of some regions. The structure shows the binding of RACK1, a signaling scaffold protein crucial in mRNA translation regulation associated with the B. divergence ribosome.
Further, combining CryoEM with nanopore sequencing reveals unique modifications at the functionally relevant ribosomal sites, not only showing the difference in ribosomal segments. New methylation in the decoding center, mRNA channel, and head-body interface. Methylation on the A28 site in the small subunit (SSU) is confirmed by nanopre and CryoEM. Methylation in Large Subunit (LSU) in the peptidyl transferase center, including helices 80, 86, 87, intersubunit bridge B2a, GTPase-associated center H95, H96, and truncated expansion segments like ES7L, ES10L, ES31L. The aforementioned modifications and end-expansion segment truncations have not been reported in other species.
They identified the unique sites of pseudouridylation, including key locations in the SSU (mRNA channel ES6S, ES7S, ES96) and in the LSU (H92, H90-91).
These discoveries indicate that the modification likely fine-tunes ribosome activity, tRNA selection, mRNA decoding fidelity, and mRNA translation efficiency.
In contrast to P. falciparum, the apicomplexan Babesia divergens has truncated extension segments, highlighting divergent evolution relative to other apicomplexans and human ribosomes.
The work supports conclusions and claims.
I do have several comments that need to be addressed:

- (line 124) For building the ribosome protein component, you used P. falciparum PDB ID 3J7A, 3J79. There are newer PDB files like 9J9H, pdb_00009J9H, https://doi.org/10.2210/pdb9J9H/pdb or 9BUS, pdb_00009bus, https://doi.org/10.2210/pdb9BUS/pdb
- (line 170-197) In the Features of Babesia RACK1 result section, several papers on RACK1 have to be added, and the comments that RACK1 does not bind P. falciparum ribosome have to be toned down. There is biochemical evidence that RACK1 binds ribosomes and actively participates in polysome assembly, suggesting its role in protein synthesis throughout the life cycle (Erath et al., 2022). Further, in Anton et al. (2023; https://doi.org/10.1101/2023.09.03.556115), they report a strong density in the 40S head region corresponding to RACK1, which was missing in the previous structure. And the paper by Go et al, 2025 (doi: 10.1016/j.str.2025.05.008. Epub 2025 Jun 5). Further, the paragraph from lines 193-197 is not supported by the abovementioned publications.

The methodology and data analysis support the conclusions, with the required changes mentioned above.

Reviewer #3

(Remarks to the Author)
General comments
In this manuscript, Cristina Gutierrez-Vargas et al., analyzed the ribosomal architecture and rRNA modification of Babesia divergens. Specifically, authors resolve the atomic structure of the cytoplasmic ribosome of Babesia divergens, a human pathogen, with the aim of finding potential drug targets. For the structural analysis, they use a new methodology, cryo-EM, that allows them to resolve in their near-native and hydrated state large, complex, and flexible supra-structural molecular complexes such as represented by ribosomes with their many post-translational modifications. Authors find important structural modifications in studied ribosomes with respect to those that have been described for other apicomplexan pathogens such as Plasmodium and Toxoplasma, but also to those of humans. These include variation of the association of the signaling protein RACK-1 with the ribosome, absence or length variation of ribosomal proteins and of expansion segments of LSU and SSU-RNA genes, and post translational modifications including methylation and pseudouridylation often at sites of functional importance. Hallmarks of this study is the high superior resolution that they achieve compared to ribosomes that have been previously analyzed by cryo-EM in Plasmodium and Toxoplasma, the finding of a high number of unique post-translations variations half of which occur at functional sites, and unique structural observations such as absence of the ribosomal protein eL28. Furthermore, the structural analysis allowed to improve the annotation of the genome of B. divergens.
The reviewer considers the contribution of much importance, because Babesia are understudied parasites and are also of high relevance in tropical and subtropical areas for the health of farm and other domestic animals. Furthermore, human babesiosis is globally expanding due to increasing traveling activity and due to climatic changes that broaden the distribution of the tick vectors. Thus, the results of this work are novel, original and highly relevant for the field of apicomplexans.
Although principally a well-written manuscript, the reviewer considers that authors generalize too much on several issues of apicomplexan pathogens Babesia, Plasmodium, and Toxoplasma, but also between different Babesia species that may lead to serious misconceptions on their biology but also to potential misinterpretations of the contextual importance of this study.
Abstract
Line 40 to 41: Babesia divergens, instead of Babesia. I suggest including more information in this section. Specifically, authors write: "Babesia is a tick-borne intracellular apicomplexan parasite responsible for diseases ranging from mild to fatal, with a broadening geographic distribution." This sentence is problematic because the reader remains disoriented with respect of the parasite that is subject of this study. To the reviewers view the reader should get from the beginning a complete understanding with which parasite the study is dealing. Babesia is a genera or genus that includes possibly more than 100 species, yet it is written as if Babesia refers to a single species. Babesia spp. are most notorious for the diseases

they cause in farm and pet animals, but accidental infections of humans occur and may result in disease or even death. In contrast, infections of wild mammals with Babesia are generally asymptomatic. If authors do not want to extend into all these details (or because of word constrains), then they should make clear in the abstract that: all Babesia are transmitted by ticks to mammals and sometimes to birds, that humans represent accidental hosts of some Babesia spp. and that this results in human babesiosis when the patient is immunocompromised (For their orientation, an important much cited overview article on babesia is: https://doi.org/10.1016/j.meegid.2012.07.004).
Line 46 "..tick-borne apicomplexan pathogen Babesia divergens.." authors should mention that cattle is the principal host and that humans are accidental hosts.
Introduction
Line 57: Arguably there are more than six Babesia species that infect humans. Authors should write "…more than eight species are known to accidentally infect humans…" (Please consult: https://doi.org/10.1017/erm.2025.10016). Maybe you want to list the most important Babesia species related to human babesiosis.
In the Introduction the following is missing: why do they study Babesia divergens and not Babesia microti? What is their justification for the parasite choice? Please provide an estimate of how many cases of B. divergens are currently observed per year and what the typical course of the disease and prognosis are. Please compare these figures and the course of the disease with those of B. microti.
Line 68: What is meant by "co-infection risk"? Do you refer to the risk of humans to be accidentally infected? Please consider that humans cannot be considered as part of the life cycle of the parasite since they are a dead-end host.
Line 70 Instead of saying that the life cycle is complex, I would think it is preferable to outline in very short the life cycle of Babesia (which is not too complex), which is the parasite that you study and that is lesser known.
Line: 73 Toxoplasma is not transmitted by an arthopod vector. Plasmodium by mosquitos and all Babesia without exception are transmitted by ticks which is a characteristic hallmark of this group of protozoan parasites and is therefore worth mentioning.
Line 74 Although it's not direct subject of your study: do you know how important is transcription regulation as compared to translational regulation in Babesia or Plasmodium parasites? It would be good to give a reference that compares transcriptional vs translational regulation through the life cycle (at different parasite stages).
Line 89 to 97: In this part you present some of the results of your study. However, typically, towards the end of the Introduction the study is justified, and the objectives of the study are defined.
Line 90: Authors generalize their results to all Babesia. This is very problematic since the genetic difference (genetic distance) between Babesia species of different phylogenetic lineages is considerable and usually largely underrated. With regard to different phylogenetic lineages of Babesia you could consult (https://doi.org/10.1016/j.pt.2019.01.007 and https://doi.org/10.1007/s00436-022-07424-8). In particular, Babesia microti belonging to the phylogenetic lineage of Clade I is on a molecular level very different to B. divergens, which belongs to the phylogenetic lineage Clade VI. To give authors an idea, even highly conserved proteins such as orthologous cysteine proteinases are difficult to align to subsequently construct a tree that gives a supporting bootstrap value between sequences of B. microti and B. divergens, which belongs to Clade VI (see Fig. 1 in https://doi.org/10.3390/vetsci5020041). Therefore, it is better to specifically refer to the parasite species that you study and raise the question how different would be its ribosome architecture to that of B. microti a Babesia species distantly related to Babesia divergens. In contrast, I would expect high similarities between ribosome structures of Babesia spp. that belong to Clade VI). Therefore, the results shown in this work are related to Babesia divergens, the Authors can't generalize to the entire genus; therefore, please, write Babesia divergens instead of Babesia. Do the same throughout the manuscript (lines 40, 132, 133, 143, 144, 185, 234, etc).
Results
Line 213: higher-eukaryote (vertebrate) specific segments (ES30L and ES44L) are absent from Babesia divergens. Is the same true for Toxoplasma sp. and Plasmodium sp.?

Methods
Line 373: Gentamicin is known to block splicing mRNA in eukaryotic cells, do the Authors consider whether the use of this antibiotic could cause artifactual changes in their observations?

Minor comments
Line 45: EM, should be introduced. Please check if according to journal policy you should introduce also the following abbreviations the first time they are used. Please, do the same in lines 114 (CTF), 128 (LC/MS-MS), 145 (LSU), 285 (CCA), 331 (cryo-FIB).
Line 120: From my point of view, both ribosomes' conformations have a tRNA bound to the E-site.
Line 125: The correct Babesia divergens strain is 1802A or 1802 (line 152)? Please, correct where it is needed. Do the same throughout the manuscript (lines 148, 157, 161, 306, etc).
Line 163: Babesia species: Please, clarify which species of Babesia.
Line 317: at the beginning of the sentence, species names should be written in full: Babesia divergens.
Line 372: Please, add Trademark and origin.

Reviewer #4

(Remarks to the Author)
I co-reviewed this manuscript with one of the reviewers who provided the listed reports. This is part of the Nature Communications initiative to facilitate training in peer review and to provide appropriate recognition for Early Career Researchers who co-review manuscripts.

Version 1:

Reviewer comments:

Reviewer #1

(Remarks to the Author)
Essentially all points have been taken into account in the revised version of the manuscript.
Some final points:
The average resolution has been re-estimated to be closer to 2.7 Å, which appears fine. But it might be worth looking into the pdb validation report, where per-chain average Q-scores can give an indication on the overall fit between the atomic model and the map. Fine features can also be evaluated from per residue resolution-dependent Q-scores (in Chimera, not in ChimeraX software; needs the MapQ (v2.9.7) plugin for that).
Regarding the pdb validation report, on page 126 it says: “This section was not generated. No FSC curve or half-maps provided”. That means that half maps (and probably masks also) have not been provided, but this would be useful for a proper evaluation here.
It is good to see that including hydrogen atoms during atomic model refinement indeed improving the clash score, as expected.
Lines 168 etc. of the answer and manuscript: “folded by Alfafold3”, probably “predicted” is meant; uL1 “remodeled”, probably modelled is meant.
Line 246 of the answer: 2.2 to 2.6 Å probably refers to local resolution; this aspect could be mentioned in the manuscript.
Lines >267: I appreciate the answer, but (original question) could there be any false positives by nanopore sequencing? Are there any RiboMethSeq data etc. available from other parasite rRNA?
“cryo-electron microscopy (cryo-EM)” should be without hyphen when written out (hyphen only for the abbreviation) to avoid the misleading impression of electrons being “cold”, hence: “cryo electron microscopy” or “cryo-EM”; to be checked throughout.
Reference 94 will be published soon, possibly update the reference later on.
Fig. S1: “Ab initio 3D model” should better say “Ab initio 3D reconstruction” (with ab initio in italics).
Fig. S2 legend: Fourier (not fourier), check throughout.
Fig. S9: pink refers to uS3, not RACK1. This seems to be a β-addition motif interaction (maybe for the main text).
Thank you for all other answers and changes in the manuscript, I have no other comments.

Reviewer #3

(Remarks to the Author)
I think the manuscript has much improved however there is the following very problematic paragraph that needs to be changed:

“Apicomplexans, including Plasmodium, Toxoplasma, and Babesia, are specialized parasitic protists that utilize an apical complex to invade host cells. Their life cycle typically involves alternating between sexual reproduction in a vector and asexual reproduction within a host. Notably, Babesia distinguishes itself by its ability to survive tick molting (Ixodidae) and to pass from mother to offspring (transovarial transmission), allowing it to persist without a vertebrate host and therefore, establish reservoirs 18–20.“

Please note that there are multiple errors and imprecisions in this part which have to be changed for the following reasons:
-First, protists include all unicellular eukaryotes while protozoa only the unicellular eukaryotse that are heterotrophs. Since protists are an extremely diverse and large group, the more specific and therefore preferred term is protozoan.
-Additionaly, Toxoplasma belongs with Eimeria and Sarcocystis to the group of coccidia, while Plasmodium and Babesi belong to hemoprotozoan. It is therefore preferable to reference this group as Toxoplasma, Plasmodium, and Babesia (that is Plasmodium neighboring Babesia).
-Furthermore, in the case of Toxoplasma there is no arthropod vector (such as a tick in Babesia and a mosquito in Plasmodium), but only a number of intermediate vertebrate hosts (vertebrate hosts are not considered vectors). Thus, for these three groups of apicomplexa, the definitive host can be a definitive vertebrate host in the case of Toxoplasma or a definitive arthropod vector host in the case of Plasmodium and Babesia, while for all three genera the intermediate host is always a vertebrate host.)
-The only hemoprotozoans that show transstadial and transovarial transmission is a subgroup of Babesia referred to as Babesia sensu stricto (e.g. B. divergens, B. crassa-like, B. venatorum, B. odocoilei, B. bovis, B. hegotelforum, and B. bigemina). Importantly, and as you mention, this characteristic allows that the arthropod-(tick) vector to function as vector and also as parasite reservoir. However, there is also the subgroup of Babesia sensu largo (e.g. B. duncani and B. microti) that shows only transstadial but no transovarial transmission. Therefore, your statement is erroneous as it assigns transovarial transmission to all Babesia. Probably the best solution is to delete this piece of information since it is of no relevance in the context of your study (transstadial transmission and transovarial transmission are relevant in an epidemiological context).

For the above reasons please delete the above paragraph and include instead the following sentence which considers and integrates all the concerns mentioned above:
"The life cycles of Toxoplasma, Plasmodium, and Babesia typically involve alternating between sexual reproduction in a definitive host and asexual reproduction within an intermediate vertebrate host18–20."
Line 41: Babesia microti instead of B. microti since it’s the start of the sentence.
Line 59: Add reference.
Line 74: Family names (Ixodidae!) should not be written in italic.

Line 167: 1802A instead of 1802.
Line 174: Add in which Babesia species.
Lines 182: Babesia divergens instead of Babesia. The same in Lines 213, 259, 321.
Line 297: amino group? Instead of imino.
Line 395, 399 and 493: five minutes or 5 minutes or 15 min? consistency throughout the manuscript. The same with seconds/sec, check please.
Line 402: tablet (Roche) instead of tablet from Roche).

Thank you for the opportunity to review this manuscript!

Reviewer #4

(Remarks to the Author)
I co-reviewed this manuscript with one of the reviewers who provided the listed reports. This is part of the Nature Communications initiative to facilitate training in peer review and to provide appropriate recognition for Early Career Researchers who co-review manuscripts.

Open Access This Peer Review File is licensed under a Creative Commons Attribution 4.0 International License, which permits use, sharing, adaptation, distribution and reproduction in any medium or format, as long as you give appropriate credit to the original author(s) and the source, provide a link to the Creative Commons license, and indicate if changes were made.
In cases where reviewers are anonymous, credit should be given to 'Anonymous Referee' and the source.
The images or other third party material in this Peer Review File are included in the article’s Creative Commons license, unless indicated otherwise in a credit line to the material. If material is not included in the article’s Creative Commons license and your intended use is not permitted by statutory regulation or exceeds the permitted use, you will need to obtain permission directly from the copyright holder.
To view a copy of this license, visit https://creativecommons.org/licenses/by/4.0/

Response to referees

The comments from **Reviewer 1** were very positive: "*The study gives very interesting novel insights into the specific nature of a ribosome from a pathogenic species. The manuscript is overall well written*" (grey). The major comments from the reviewer concerned the global and local resolution of the structural models, as well as the refinement of the head of the small ribosomal subunit. We address these comments (black) thoroughly in this rebuttal (red). We thank the reviewer for their helpful comments on this manuscript.

The comments from **Reviewer 2** were very positive: "*I would highly recommend publishing the manuscript "Ribosomal Architecture and rRNA Modification Landscape in the Tick-Borne Parasite Babesia divergens" by Gutierrez Vargas et al. with the additional revisions mentioned below: Apicomplexans, obligate intracellular parasites, have highly reduced genomes, a complex life cycle that involves multiple hosts, and specialized cellular structures that reflect their parasitic lifestyle. The structure of apicomplexan ribosomes presents a remarkable example of cellular adaptation to intracellular parasitism*"… "*The work supports conclusions and claims*." The reviewer had minor comments, which we believe are fully addressed below.

The comments from **Reviewers 3 and 4** were also very positive: "*Thus, the results of this work are novel, original, and highly relevant for the field of apicomplexans*." The major concerns were that we appeared to generalize too much on several issues regarding apicomplexan pathogens, *Babesia*, *Plasmodium*, and *Toxoplasma*, as well as between different *Babesia* species, "which could lead to serious misconceptions about their biology but also to potential misinterpretations of the contextual importance of this study." As described below, to keep the manuscript concise, we referred to *B. divergens* throughout without explicitly stating this, which could have given the impression that we were generalizing. We hope that the revised version will satisfy Reviewers 3 and 4.

Reviewer #1 (Remarks to the Author)

The manuscript by Gutierrez-Vargas et al. reports on the structural analysis of a peculiar eukaryotic ribosome that was isolated from a tick-borne parasite. The study focuses on the structural aspects and hints at functional implications of the specific structural features of this ribosome, such as the rRNA and ribosomal proteins (e.g., eL28 appears to be specifically absent, RACK1 is well visible) and chemical modifications of the rRNA (cross-validated by nanopore analysis). Two cryo-EM structures were obtained at 3.1 and 2.6 Å resolution with either 3 tRNAs or only E-site tRNA bound, respectively. The study gives very interesting novel insights into the specific nature of a ribosome from a pathogenic species. The manuscript is overall well written, but some aspects could be clarified as detailed below.

Detailed points:

- abstract and text: the therapeutic applications are certainly interesting per se, but maybe these need not be emphasized too much at this stage, as direct conclusions on drug design are not drawn here yet. These are just new findings, which are interesting by themselves.

We agree with the reviewer that, in both the abstract and the main text, the therapeutic potential may have been emphasized too strongly. Therefore, we removed the final portion of lines 53 from the abstract: "…uncovering new avenues for the therapeutic intervention against babesiosis."

We also removed the last sentence of the discussion (originally line 365): "The divergent rRNA modifications observed in *Babesia* species relative to their human hosts may provide a unique opportunity for selective therapeutic targeting."

- abstract and text: implications in gene expression is a general statement that applies to other species also, it could be clarified what is specific about it here.

This comment relates to a similar comment from Reviewers 3 and 4 (see lines 513-525 of this rebuttal), which we address in more detail below. We now specifically describe the complexity of the *Babesia* life cycle and relate it to the need for gene regulation to adapt and survive during tick molting and feeding (lines 72-77).

- abstract: no need to repeat "tick-borne apicomplexan pathogen."

We agree with the reviewer and have removed: "…the tick-borne apicomplexan pathogen… (now line 47).

- "atomic resolution": even though the resolution of 2.6 Å is very good, in crystallography, people would still argue that this is not atomic because individual atoms are not resolved (which would require a resolution closer to 1 Å).

We agree with the reviewer and have changed "atomic" to "high" (now line 49). We also removed from the abstract the specific resolution.

- resolution estimation: the estimated overall resolution is indicated with 2.6 Å, but the estimation from map-model FSC is 2.74 Å, which may indicate some overestimation. What is the d99 value that the Phenix software provides? Also, the average resolution of 2.6-2.7 Å indicates that it is unlikely to have data extending to 1.7 Å. The features in the map do not support that really. The conclusions drawn on the structure appear solid, so there is no need to overstate resolution.

We thank the reviewer for bringing this to our attention. After reevaluation of the data, we agree with the reviewer that the global resolution should be 2.7 Å with local resolution reaching 1.8 Å. In support of this, we are including a figure (see below) for the reviewer showing the distribution of voxel counts across bin resolutions. Following this reanalysis, we adjusted the stated resolution to the corrected number throughout the manuscript and in the figure legends (**Figs. 1, S1,** and **S2**).

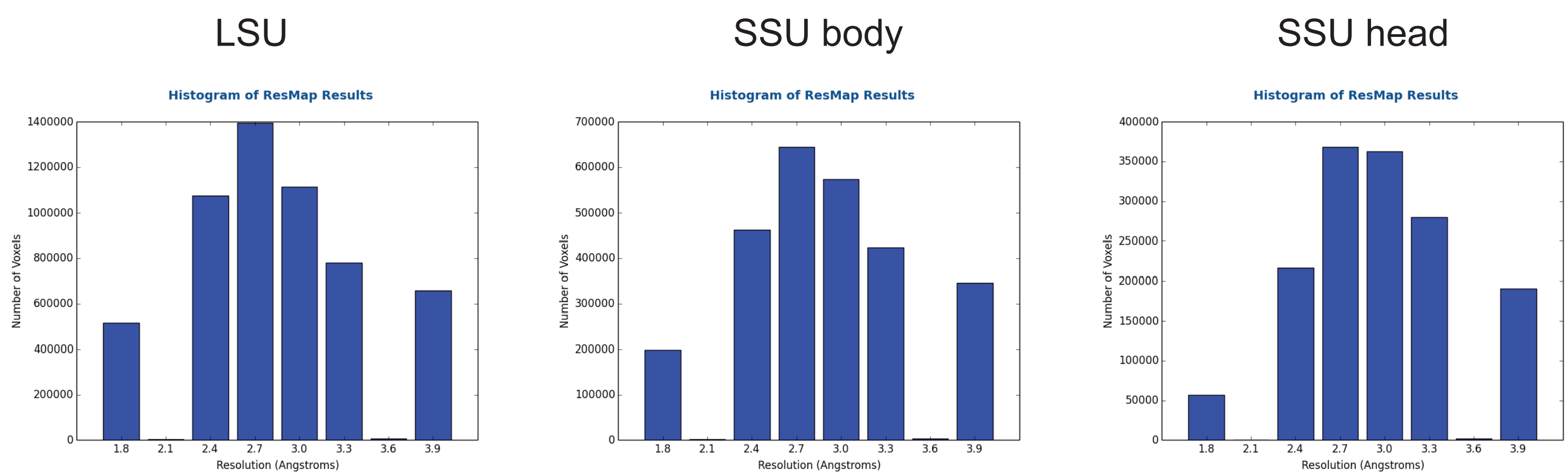

2D histograms of local resolution values for the large subunit (LSU), the body and the head of the small subunit (SSU) reconstructions obtained from multibody refinement.

- introduction:

Explain apicomplexan

As requested by the reviewer, we included an explanation of apicomplexan. The text was changed from: "Apicomplexan parasites, such as *Plasmodium*, *Toxoplasma,* and *Babesia*, have complex life cycles".

To: "Apicomplexans, including *Plasmodium*, *Toxoplasma,* and *Babesia*, are specialized parasitic protists that utilize an apical complex to invade host cells (now lines 71-72).

- it is interesting to see that some new modification sites are being proposed. However, some of these are only found according to the nanopore sequencing data, but not visible in the structure. How reliable are these? Until recently, nanopore analysis of chemical modifications was considered complementary for validation, but not very precise by itself as a standalone technique. Is there independent evidence from MS, RiboMethSeq, or other techniques for the new sites? Otherwise, it might be better to list these sites apart or label them differently.

As this comment relates to others from the reviewer, regarding rRNA modifications, we grouped our answers, starting from line 225 of this rebuttal.

- methylation sites: the description in the results and discussion discusses 2'-O-methylations and nucleotide base methylations simultaneously, which is a bit confusing. It would be easier to follow if discussed separately. These are distinct types of modifications, also implying a different chemical reactivity and hydrogen-bonding properties in the structure.

See our consolidated answer related to modification comments (lines 225-258 of this rebuttal).

line 104: morphology of 80S ribosomes: compared to which structure/organism?

In the manuscript, we originally used the term "typical morphology of an 80S ribosome" to indicate that the subunits were fully assembled and that the particle's size matched that of a eukaryotic ribosome (larger than that of a prokaryotic ribosome), rather than highlighting a specific eukaryotic organism. Following the reviewer's comments, we acknowledge that we could be more precise. We therefore changed the text: "distinctive morphology of ribosomes, in which both subunits are present" (now line 112).

line 109: human 1824/1825: reference? e.g. PMID: 25901680

We agree that references should be included for the 18S rRNA sequence numbering. We have added the reference PMID: 25901680, as suggested by the reviewer, as well as the reference with PMID: 23636399 (now line 118).

line 113: data not shown, but the stacking inside helix h44 could be interesting to show

As suggested by the reviewer, we added a figure (new **Fig. S3**), showing the stacking of A1683 and A1684 within H44 from *B. divergens*, as observed in ribosomes containing a single E-site tRNA (conformational state 2). All the supplemental figures were renumbered accordingly (now line 121).

- cryo-EM data processing and usage of local/focused classifications and refinements and multibody refinement strategies: some references could be useful here; e.g., PMID: 14985767 (original concept in the field); PMID: 27572726 (review); PMID: 37659578 (recent paper & refs therein); PMID: 29856314 (multibody refinement)

We agree with the reviewer, and we have added these references to the *Methods* section to improve clarity.

- line 122: this is local resolution

We agree with the reviewer that 1.7 Å refers to the local resolution. Therefore, the text was changed from:

"…with areas within the ribosome core reaching atomic resolution (approximate resolution of 1.7 Å, **Fig. 1c**)."

To: "…with areas within the ribosome core reaching high resolution (local resolution approximation of 1.8 Å, **Fig. 1c**)." Now line 130.

- Fig. 1c, right: it seems that the 40S ribosomal subunit head could be refined further; apparently, focused refinement and multibody refinement were applied, but was this with partial signal subtraction and recentering of the particle images?

We thank the reviewer for bringing this to our attention. Our initial multibody refinement did not include signal subtraction and recentering of the particle images. We therefore reprocessed the data to include these steps. This procedure resulted in a reconstruction with a global resolution of ~2.8 Å for the SSU head. A visual comparison of this map with the SSU head map obtained from multibody refinement (global resolution of ~2.7 Å) indicated that the interpretable density is largely identical between the two approaches. Consistent with this observation, the map-map correlation between the two reconstructions is high (fit-in-map correlation = 0.9785). Therefore, we retained the multibody refinement map because it provided better resolution. We have included on the right a figure showing the alignment between the two maps, contoured at 10σ for comparison. We also added a short description in the Methods section mentioning that this approach was also tried:

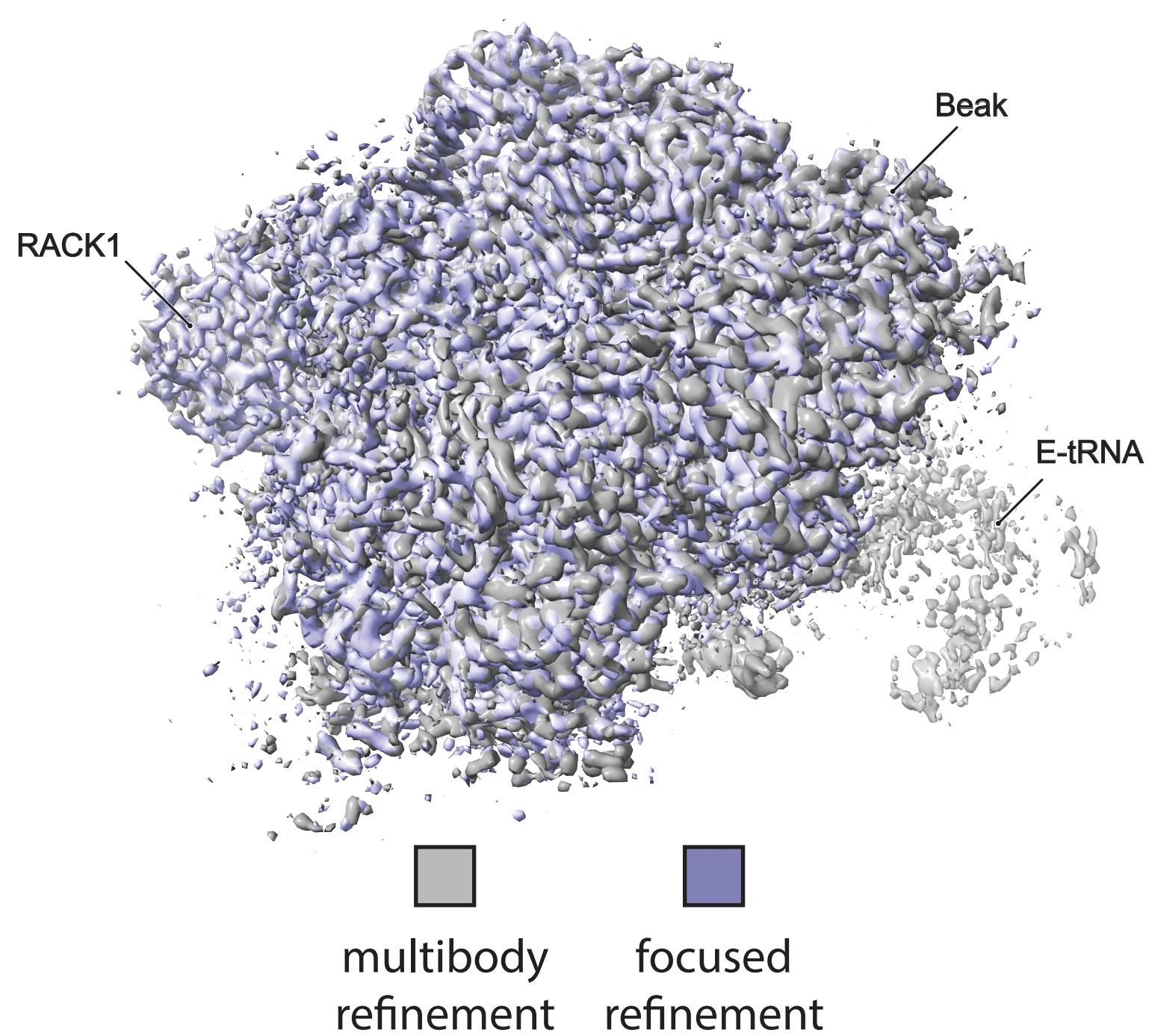

"We also tried partial signal subtraction with centering (center-subtracted images on mask option enabled), followed by focused refinement of the SSU head[93]. Still, it did not result in any additional interpretable density for model building compared with the map obtained from multibody refinement" (now lines 449-453).

- Fig. 2 legend: "threshold" should better say "contour" level, as no cut of the data is done when displaying maps; to be checked throughout

We agree with the reviewer. After careful revision, the Figure legends of **Fig. 2** and **Fig. S3** were modified, replacing "threshold" with "contour".

- pseudouridine N1 coordination by a water molecule: this has been seen in other ribosome structures as well; maybe mention some. Are these exactly in plane with the nucleotide base? They should normally be.

We have included an appropriate reference for this (see similar comment below). To answer the reviewer's question, yes, the water coordination is planar with the pseudouridine base. We also added a sentence in the legend of Fig. 6 to indicate so: "Among all the pseudouridylations observed by cryo-EM, all are coordinated with a planar water molecule."

- same for Zn2+ and Mg2+ ions, tetrahedral and octahedral have been described in detail for some other ribosome structures including human (PMID: 38844527); it seems that the concept of that described in lines 136-138 is taken from there.

The reviewer is absolutely correct, and we thank him for catching this omission. We have added the reference (now line 147).

- lines 150/151: not sure to understand this phrase but it seems obvious that introns are not present anymore in the maturated mRNA coding for the corresponding proteins. Does it imply that the genome annotations are incomplete? Interesting though to see it validated here from the protein sequence in the structure.

We thank the reviewer for pointing out that our wording was unclear. To answer the reviewer's question directly: we meant that these genomic regions were not annotated as introns; however, the RNA seq data agreed with our structural model, indicating that the "missing sequences" correspond to intronic regions in the genome. Therefore, we changed the sentence from:

"We found that *Babesia* uS7 (residues 70-97), eS10 (residues 26-46), uS12 (residues 64-106) (**Fig. S3**), eL18 (residues 122-146) and eL24 (residues 61-72) (**Fig. S4**) lack these sequence regions, as initially annotated in the *Babesia* 1802A strain genome. These findings are based on the density map analysis, which shows no corresponding density for these portions of the protein sequences. RNA-seq data also support these observations, indicating the presence of introns that would remove the sequences coding for each of those regions."

To: "We found that our cryo-EM density maps could not accommodate several sequence regions from *Babesia* uS7 (residues 70-97), eS10 (residues 26-46), uS12 (residues 64-106) (**Fig. S3**), eL18 (residues 122-146) and eL24 (residues 61-72) (**Fig. S4**), as previously annotated in the genome of the *Babesia* 1802A strain. After reexamining the corresponding RNA-seq data for each of these proteins, which likewise lacked these sequences, we conclude that the corresponding genomic regions are in fact misannotated introns." (now lines 156-161)

- L1 and P stalk are not visible, but usually in the presence of E-site tRNA (state 2), the L1 stalk is stabilized (e.g., in PMID: 27665925, where we discussed this). Maybe filtering the map to ~6 Å resolution could help visualizing the L1 stalk if it is less ordered. Is the E-site tRNA fully defined structurally on both 40S and 60S sides?

We noted the reviewer's suggestion and reexamined lower-resolution filtered maps to see if we could visualize the L1 stalk. Indeed, we see density for the L1 stalk, which allowed us to dock *B. divergens* uL1 folded by Alfafold3. However, in the composite map, the density for uL1 is greatly diminished; hence, we did not remodel it. We included this modification in the manuscript, cited PMID: 27665925, and added a new supplementary figure showing the differences between the maps (now **Fig. S7**). The supplementary figures were renumbered accordingly.

Therefore, the text was changed from:

"We did not model the proteins forming the ribosome L1 (uL1) and P stalks (uL10, uL11, P1, P2), because their corresponding density were too weak, despite being present in our sample based on mass spectrometry analysis (**Tables S5-S7**)."

To: "We did not model the ribosomal proteins forming the P stalk (uL10, uL11, P1, P2), because their corresponding densities were too weak, despite their presence in our sample as determined by mass spectrometry analysis (**Tables S5-S7**). As for the L1 stalk, formed by the ribosomal protein uL1, we were able to dock uL1 into a low-pass-filtered map at 6 Å[49]. However, only partial density remained visible in the composite maps (**Fig. S7**)." (now lines 174-178).

- line 176: uS3 in the human ribosome structure: mention which structure you refer to (probably PMID: 25901680).

Same for lines 183 and 188. It is interesting to see the comparison with human ribosome structure that is being discussed, also because this may provide hints at how to generate species-specific drugs. Same regarding comparisons of chemical modifications (this would be PMID: 38844527).

We thank the reviewer for pointing this out and have included the suggested references.

- lines 192 etc.: the explanation for the apparent differences in RACK1 sequences might be fine, but maybe sample purification and image processing/structure sorting have led to identify the most stable protein species?

We fully agree with the reviewer. However, to avoid potentially upsetting other groups that have worked on RACK1 in apicomplexans, we would prefer not to change this portion of the text.

- rRNA expansion segments (ESs): interesting aspect; line 206: human ESs: which reference

We included the reference of Anger et al, PMID: 23636399 (now line 221)

- annotation of ES elements: usually these carry the extra letter L or S to specify to which ribosomal subunit they belong; e.g., ES12S instead of ES12, etc. (line 210 and possibly elsewhere)

We thank the reviewer for identifying this issue. We have reviewed the manuscript and verified that the ES nomenclature is now consistent throughout the text (e.g., ES12S, ES7L, etc.).

- it is interesting to see that ribosomal protein eS28 is absent; is this possibly due to an expansion of ES10L (Fig. 4b)? or some other relationship between ES differences and eS28?

We thank the reviewer for his interest regarding the absence of eL28, which we also found to be a striking feature. The reviewer's comment made us realize that the placement of the eL28 description could be confusing, since we immediately turn to ES10L afterward. We therefore modified the text from:

"… This is contrasting with the equivalent area in *Toxoplasma*, where ES15L forms a pseudoknot with ES7Lc, which most likely reduces its flexibility (**Figs. 4c and 4d**).

eL28 is absent from the *Babesia* ribosome cryo-EM density (**Fig. 4a**). Although we identified eL28 homologs in other *Babesia* species that are pathogenic to humans, such as in *B. duncani* and *B.* microti, we did not identify eL28 in the genome of *B. divergens* strains. eL28 was also absent from mass spectrometry data analysis (**Table S6**). Within the same vicinity, ES10L in *Babesia* is expanded compared to *Plasmodium* and *Toxoplasma,* respectively (**Figs. 4b, c, and**)." to

"This is contrasting with the equivalent area in *T. gondii*, where ES15L forms a pseudoknot with ES7Lc, which most likely reduces its flexibility (**Figs. 4c and 4d**). Within the same vicinity, we found that eL28 is absent from the *Babesia* ribosome cryo-EM density (**Fig. 4a-c**). Although we identified eL28 homologs in other pathogenic *Babesia* species, such as *B. duncani* and *B. microti*, we did not identify eL28 in the genome of the *B. divergens* 1802A strain. eL28 was also absent from the corresponding mass spectrometry data (**Table S6**).

Sitting towards the back of ES7Lc, we found that ES10L is expanded in *Babesia* compared with *P. falciparum* and *T. gondii,* respectively (**Figs. 4b, c, and e**). While *Toxoplasma* ES10L is located underneath ES9L and ES12L (**Fig. 4c**), *Babesia* ES10L extends along the solvent-exposed surface and predominantly contacts uL30 (**Figs. 4b and 4e**). (now lines 254-258)

Further reasons why we believe eL28 is lost are also provided in the Discussion section: "… the cryo-EM dataset showed no corresponding density for the ribosomal protein eL28, whereas this protein is present in *P. falciparum*[29,30,32] and *T. gondii* cryo-EM ribosome structures[34]. In *P. falciparum*, eL28 interacts at the junction of two expansion segments, ES7La and ES15L, both of which are much shorter in *B. divergens*. We hypothesize that the reduction of the ESs in *B. divergens* may have led to the loss of eL28."

- line 262: specify which methylation type is being discussed

We thank the reviewer for noting that this part of the manuscript could be clearer. We now explicitly specify the types of methylations, modifying the text from: "By combining cryo-EM density map analysis with nanopore sequencing, we identified a total of 66 methylation sites (**Fig. 5**)." to

" By combining cryo-EM density map analysis with nanopore sequencing, we identified a total of 61 methylation sites. Among these, five corresponds to base methylations (m^7G, $m^6{}_2A$ (2), m^1A, and m^5C), which were observed in the cryo-EM density map, while the others correspond to 2'-*O*-ribose methylations (2'-*O*-Me) (**Fig. 5**)." (now lines 278-281).

- line 263-265: if there are about 30 new methylation sites and only a quarter of those visible, are the other 3/4 just not resolved, possibly? The description here could benefit from discussing 2'-O-methylations and nucleotide base methylations separately

We thank the reviewer for bringing this to our attention and have rewritten this section. It originally read: "Out of the 66 methylation sites identified, 59 were detected using nanopore sequencing, where more than 80 % are within the ≥ 50 % threshold. Among those 59 rRNA methylations, about half were new methylation sites, with a quarter of those being observable in the cryo-EM density maps. Among all the methylations identified, 2'-*O*-Me modifications were the most frequent, with very few m^1 and m^6 nucleotide methylations." to:

"Out of the 56 2'-*O*-Me sites identified, three modifications were identified exclusively by cryo-EM density maps analysis (Gm43, Am658, and Gm2899), while 31 modifications were identified by cryo-EM and nanopore sequencing. Nanopore sequencing allowed the identification of 22 additional sites, among which more than 50 % exceed the ≥ 50 % threshold" (now lines 281-285).

Among the ¾ of additional sites (21) identified by nanopore sequencing but not observed within the cryo-EM maps, all are found at a high enough resolution (2.2 to 2.6 Å) that if they were present, we should have been able to observe them as well. We found that, on average, these sites are modified at a lower occurrence (between 30-50%). We conclude that lower occurrence reflects the variability of these sites being modified throughout the developmental stage of the *B. divergens* within red blood cells. We clarified this idea by explicitly mentioning this in the text:

"We complemented our strategy with nanopore sequencing[69,70], which could enable the identification of rRNA modifications in regions of the ribosome with lower resolution. Also, because we used asynchronous cultures, nanopore sequencing could allow the identification of rRNA modifications that may be present at a specific developmental stage. We hypothesize that if that were the case, these modifications would have a lower occurrence, which would be averaged out in the cryo-EM dataset, and therefore, would be within noise level. For this reason, we applied two different rRNA modification occurrence thresholds during nanopore sequencing analysis: modifications identified and present more than 50 % (≥ 50 %) and between 30 and 50 % (≥ 30 % and ≤ 50 %) for a given position (see *Methods*). (lines 269-277)

- Fig. 5c: the parallel lines are a bit confusing, a star-like labeling would be easier + less overlapping with the structure behind

We agree with the reviewer and have modified **Figs. 5c and 6c** accordingly to avoid confusing overlapping lines.

- Figs 5 & 6a: there seems to be many new suggested sites; see comment above, as there are still relatively many not visible in the structure, are there other data (RiboMethSeq) to confirm this independently? If not, it is not a problem as this is a new species being analysed, but it may be good to clarify how far nanopore sequencing alone is safe to annotate new sites. Could there be any false positives by nanopore sequencing? Any data available from other parasite rRNA?

To the best of our knowledge, we are the first group to use Oxford Nanopore sequencing (basecaller Dorado 0.9.0) to detect de novo pseudouridine and Dorado v1.0.2 to additionally detect 2'-*O*-methylations in parasites. Nanopore sequencing has proven reliable for identifying modification sites, especially when using direct RNA sequencing with custom oligos targeting the 3' ends of the rRNAs (one each for 18S and 28S), instead of relying on an intermediate polyadenylation step. However, the method becomes less accurate for hypermodified molecules, e.g., tRNAs.

It would be possible to use either MS-HPLC or RiboMethSeq as complementary approaches. Still, these methods are less cost-effective than nanopore sequencing and require more material. Both points were limiting in the design of our experimental strategy.

Regarding the accuracy of nanopore sequencing specifically for this study, we added a few lines in the Method section, specifying the confidence in the modification state for each nucleotide relative to the 18S rRNA and 28S rRNA samples provided for analysis.

"Per-site coverage was capped (maximum depth) at 7.5 million reads. Modkit automatically determined a confidence threshold (10th percentile of the site-specific probability distribution) to exclude low-confidence basecalls. The resulting thresholds were 73.8% (C), 84.0% (U), 92.4% (G), and 75.6% (A) for 18S rRNA, and 73.0% (C), 73.4% (U), 83.4% (G), and 65.8% (A) for 28S rRNA. For pseudouridine modification calls, the thresholds were 79.1% and 77.9% for the 18S and 28S, respectively. Modification levels (occurrence) were calculated as the percentage of modified calls passing thresholds among the total number of calls at a given nucleotide (Nmod/Nvalid_cov × 100). I" (now lines 562-569). While the "A" nucleotide threshold is the lowest, with an average of ~71 %, only four methylated "A" nucleotides within the new modification sites were exclusively identified by nanopore sequencing, all of which were within the >50% occurrence threshold. Therefore, we assume that those are less likely to be false positives.

- line 273: specify which methylation type

We now clarify that these refer to 2'-*O*-Me. (now line 279)

- line 278: characteristic hydrogen bonding on the N1 position: indeed, as seen in the human ribosome structure that is being compared with afterwards (PMID: 38844527); the new PSU sites are interesting indeed.

We thank the reviewer for this clarification. We modified the text from: "Pseudouridylations are more challenging to identify in cryo-EM maps because they require high local resolution to observe their characteristic hydrogen bonding (Fig. 2e). to:

"Pseudouridylations are more challenging to identify in cryo-EM maps because they require high local resolution to observe their characteristic hydrogen bond formed between the N1 imino group (N1H) and a water molecule, as previously described in human ribosomes **(Fig. 2c)**[45]." We also added the corresponding citation (now lines 297-299).

- line 279: ref 64 is an MS-based analysis, ref 43 used MS too; there is also HydraPsiSeq, etc.

We thank the reviewer for pointing this out. We added a reference for the HydraPsiSeq method and included an additional review on methods for detecting pseudouridines.

- discussion: L1 and P stalks: see comment above. That the P stalk is not visible is not surprising, but the L1 stalk would be expected to be better ordered in the E-site tRNA complex.

As mentioned above, we are further exploring whether filtering to lower resolution reveals additional L1 stalk density. At present, the P stalk is not visible, and the L1 stalk remains poorly ordered despite the presence of E-site tRNA.

- line 325: RACK1 in yeast: was this not also seen in the yeast 80S ribosome crystal structure (PMID: 22096102)? It is also present in the human ribosome structure (PMID: 25901680)

The reviewer is correct, and the citations have been added.

- methods: Relion, etc.: the software Relion, etc.

We have implemented that change (now line 430).

- lines 426-439: the procedure is good, one could include some refs for focused classification and refinement, and multibody refinement

This comment has been addressed above; we have added the suggested references for focused classification, focus refinement, and multibody refinement.

- nanopore sequencing (line 533 etc.): the procedure seems to use some new features, which could be explained more; are there any scripts, etc., that could be provided to the readers to help reproducibility, etc.?

We did not develop any custom scripts for our analysis. Thus, other groups should be able to reproduce or adapt our nanopore sequencing approach, given that we provide the thresholds, basecaller versions, and the number of reads used.

- references to resolution estimation from Fourier shell correlation (FSC) typically comprise 1) van Heel M., Keegstra W., Schutter W. G. & van Bruggen E. F. J. Arthropod hemocyanin studied by image analysis. Life Chem Rep Suppl 69–73 (1982). 2) Saxton, W. O. & Baumeister, W. The correlation averaging of a regularly arranged bacterial cell envelope protein. J. Microsc. 127, 127–138 (1982). 3) Rosenthal, P. B. & Henderson, R. Optimal determination of particle orientation, absolute hand, and contrast loss in single-particle electron cryomicroscopy. J. Mol. Biol. 333, 721–745 (2003).

We thank the reviewer for highlighting this. Instead of referring to the FSC only in **Fig. S2**, we added a sentence in the Methods section to incorporate these references. Therefore, the text was modified from: "We extended the binary map by 4 pixels in every direction and added a cosine-shaped soft-edge of 4 pixels, resulting in an 80S map with a global resolution of 2.6 Å." to:

"We extended the binary map by 4 pixels in every direction and added a cosine-shaped soft edge of 4 pixels, resulting in an 80S map with a global estimated resolution of 2.7 Å (**Fig. S2**), followed by the references suggested by the reviewer. (now lines 445-447)

- validation report for the refined coordinates: there are quite some close contacts; it could help to refine the atomic model with the Phenix software, including hydrogen atoms, which help remove clashes due to the van der Waals contacts being taken into account better. Those are not visible but help refining (as riding atoms with predefined geometry) and do not need to be included in the deposited model.

We appreciate the reviewer's suggestion and have made additional refinement, including hydrogens. For 3D class 3, while all other statistics remained barely unchanged, the clash score decreased from 5.6 to 2.7 (Phenix refinement values) and from 5 to 3 (PDB EM Validation Report). Therefore, the new models have been deposited in the PDB for both Class 1 and Class 3, and Tables S1 and S2 have been updated accordingly.

- generic point: cryo-EM maps, half maps, masks, etc., and fully refined atomic models should be deposited in the EMDB and PDB; deposition of representative data onto the EMPIAR database could be considered also.

All of these have been deposited in their appropriate database. All the access codes are provided in Tables S1 and S2 and will be released upon manuscript publication.

Thank you for the opportunity to review this work.

Bruno Klaholz

Review #2 (Remarks to Author)

I would highly recommend publishing the manuscript "Ribosomal Architecture and rRNA Modification Landscape in the Tick-Borne Parasite Babesia divergens" by Gutierrez Vargas et al. with the additional revisions mentioned below:

Apicomplexans, obligate intracellular parasites, have highly reduced genomes, a complex life cycle that involves multiple hosts, and specialized cellular structures that reflect their parasitic lifestyle. The structure of apicomplexan ribosomes presents a remarkable example of cellular adaptation to intracellular parasitism.

In this manuscript, the structure of the Babesia divergence ribosome is reported as the first one from Babesia spp. And one of the few in the realm of apicomplexan. The CryoEM structure of Babesia divergence is 2.6 A resolution, with 1.7 A resolution of some regions. The structure shows the binding of RACK1, a signaling scaffold protein crucial in mRNA translation regulation associated with the B. divergence ribosome.

Further, combining CryoEM with nanopore sequencing reveals unique modifications at the functionally relevant ribosomal sites, not only showing the difference in ribosomal segments. New methylation in the decoding center, mRNA channel, and head-body interface. Methylation on the A28 site in the small subunit (SSU) is confirmed by nanopre and CryoEM. Methylation in Large Subunit (LSU) in the peptidyl transferase center, including helices 80, 86, 87, intersubunit bridge B2a, GTPase-associated center H95, H96, and truncated expansion segments like ES7L, ES10L, ES31L. The aforementioned modifications and end-expansion segment truncations have not been reported in other species.

They identified the unique sites of pseudouridylation, including key locations in the SSU (mRNA channel ES6S, ES7S, ES96) and in the LSU (H92, H90-91).

These discoveries indicate that the modification likely fine-tunes ribosome activity, tRNA selection, mRNA decoding fidelity, and mRNA translation efficiency.

In contrast to P. falciparum, the apicomplexan Babesia divergens has truncated extension segments, highlighting divergent evolution relative to other apicomplexans and human ribosomes.

The work supports conclusions and claims.

I do have several comments that need to be addressed:

• (line 124) For building the ribosome protein component, you used P. falciparum PDB ID 3J7A, 3J79. There are newer PDB files like 9J9H, pdb_00009J9H, https://doi.org/10.2210/pdb9J9H/pdb or 9BUS, pdb_00009bus, https://doi.org/10.2210/pdb9BUS/pdb

We thank the reviewer for bringing this to our attention. At the time when the *B. divergens* ribosome model was done, these PDBs were not available. Therefore, we listed the one we used. To clarify, we used only the *P. falciparum* ribosome structure to retrieve ribosomal protein sequences. Those sequences were then blasted against PiroplasmaDB to identify *B. divergens* homologues, which were then folded and docked independently of the *P. falciparum* ribosome structure used. However, we do cite the work mentioned by the reviewer in our manuscript in other circumstances.

• (line 170-197) In the Features of Babesia RACK1 result section, several papers on RACK1 have to be added, and the comments that RACK1 does not bind P. falciparum ribosome have to be toned down. There is biochemical evidence that RACK1 binds ribosomes and actively participates in polysome assembly, suggesting its role in protein synthesis throughout the life cycle (Erath et al., 2022). Further, in Anton et al. (2023; https://doi.org/10.1101/2023.09.03.556115), they report a strong density in the 40S head region corresponding to RACK1, which was missing in the previous structure. And the paper by Go et al, 2025 (doi: 10.1016/j.str.2025.05.008. Epub 2025 Jun 5). Further, the paragraph from lines 193-197 is not supported by the abovementioned publications.

We thank the reviewer for pointing out that the paragraph describing the interaction between RACK1 and the SSU was somewhat confusing. The reviewer is right that we omitted to cite Erath's work and have included this reference. Relative to Go et al. and Anton et al., we did cite those references in the discussion. We agree with the reviewer that that section should be included in the results section to make our conclusions stronger and clearer.

Therefore, the text in the manuscript was changed from: "The molecular details of RACK1 and ribosomes purified from apicomplexans untreated with any drugs have not been reported, as density for RACK1 was either not observed or very weak, and unmodeled in *Plasmodium* and *Toxoplasma* single-particle cryo-EM ribosome structures, respectively[29,30,33,34]. … This observation would not be sufficient to explain the absence of RACK1 in the other apicomplexan ribosome structures mentioned above. Other factors may contribute to this discrepancy, such as during cryo-EM sample preparation or the fact that the *Babesia* ribosome preparation encompasses all the host-parasite's life stages, as the culture was asynchronously grown.

To: In previous single-particle cryo-EM ribosome structures from *Plasmodium* and *Toxoplasma*, density for RACK1 was either not observed or very weak, and unmodeled[32,35,36]. However, using homology modeling and cellular and molecular biology studies, RACK1 from *P. falciparum* was found to associate with ribosomes from polysome fractions[57]. Moreover, *in situ* structures of ribosomes from cryo-FIB milled from *P. falciparum*-infected human erythrocytes across all asexual stages show the presence of RACK1 on the SSU, although the lower resolution limited the detailed characterization of the interaction[34]. … Although the interactions between RACK1 and eS17 are of the same nature in human ribosomes and in the *B. divergens* ribosome (hydrogen bonds, salt bridges, and hydrophobic contacts), the binding interface is slightly larger in *Babesia*, where five eS17 residues interact with RACK1 compared to four in humans. The molecular details of the interaction between RACK1 and the SSU from *B. divergens* are consistent with a recent study describing the interaction between RACK1 and the ribosomes from *P. falciparum* when treated with artemisinin[31] (now lines 192-212).

The methodology and data analysis support the conclusions, with the required changes mentioned above.

Reviewer #3 (Remarks to the Author)

General comments

In this manuscript, Cristina Gutierrez-Vargas et al., analyzed the ribosomal architecture and rRNA modification of Babesia divergens. Specifically, authors resolve the atomic structure of the cytoplasmic ribosome of Babesia divergens, a human pathogen, with the aim of finding potential drug targets. For the structural analysis, they use a new methodology, cryo-EM, that allows them to resolve in their near-native and hydrated state large, complex, and flexible supra-structural molecular complexes such as represented by ribosomes with their many post-translational modifications. Authors find important structural modifications in studied ribosomes with respect to those that have been described for other apicomplexan pathogens such as Plasmodium and Toxoplasma, but also to those of humans. These include variation of the association of the signaling protein RACK-1 with the ribosome, absence or length variation of ribosomal proteins and of expansion segments of LSU and SSU-RNA genes, and post translational modifications including methylation and pseudouridylation often at sites of functional importance. Hallmarks of this study is the high superior resolution that they achieve compared to ribosomes that have been previously analyzed by cryo-EM in Plasmodium and Toxoplasma, the finding of a high number of unique post-translations variations half of which occur at functional sites, and unique structural observations such as absence of the ribosomal protein eL28. Furthermore, the structural analysis allowed to improve the annotation of the genome of B. divergens.

The reviewer considers the contribution of much importance, because Babesia are understudied parasites and are also of high relevance in tropical and subtropical areas for the health of farm and other domestic animals. Furthermore, human babesiosis is globally expanding due to increasing traveling activity and due to climatic changes that broaden the distribution of the tick vectors. Thus, the results of this work are novel, original and highly relevant for the field of apicomplexans.

Although principally a well-written manuscript, the reviewer considers that authors generalize too much on several issues of apicomplexan pathogens Babesia, Plasmodium, and Toxoplasma, but also between different Babesia species that may lead to serious misconceptions on their biology but also to potential misinterpretations of the contextual importance of this study.

Abstract

Line 40 to 41:

Babesia divergens, instead of Babesia. I suggest including more information in this section. Specifically, authors write:" Babesia is a tick-borne intracellular apicomplexan parasite responsible for diseases ranging from mild to fatal, with a broadening geographic distribution." This sentence is problematic because the reader remains disoriented with respect of the parasite that is subject of this study. To the reviewers view the reader should get from the beginning a complete understanding with which parasite the study is dealing. Babesia is a genera or genus that includes possibly more than 100 species, yet it is written as if Babesia refers to a single species. Babesia spp. are most notorious for the diseases they cause in farm and pet animals, but accidental infections of humans occur and may result in disease or even death. In contrast, infections of wild mammals with Babesia are generally asymptomatic. If authors do not want to extend into all these details (or because of word constrains), then they should make clear in the abstract that: all Babesia are transmitted by ticks to mammals and sometimes to birds, that humans represent accidental hosts of some Babesia spp. and that this results in human babesiosis when the patient is immunocompromised (For their orientation, an important much cited overview article on Babesia is: https://doi.org/10.1016/j.meegid.2012.07.004).

We thank the reviewer for putting us back on track. Unfortunately, some of these specifications were present in previous versions that were lost during revisions aimed at shortening the manuscript. We appreciate the reviewer's perspectives, which help clarify that the study focuses on *B. divergens*.

Following the reviewer's comment, we changed the abstract's first few sentences from: "*Babesia* is a tick-borne intracellular apicomplexan parasite responsible for diseases ranging from mild to fatal, with a broadening geographic distribution."

To: "*Babesia* are tick-borne intracellular apicomplexan parasites that infect a wide range of wild and domestic animals (e.g., cattle), resulting in significant economic losses to the livestock industry. Humans are considered accidental hosts for a few *Babesia* species. *B. microti* and *B. divergens* are the most prevalent causes of human babesiosis that are showing a broadening geographic distribution." (now lines 39-43)

Line 46 "...tick-borne apicomplexan pathogen Babesia divergens." authors should mention that cattle is the principal host and that humans are accidental hosts.

This point is addressed in the modifications described above (lines 39-41), where we clarify the host range and the accidental nature of human infections.

Introduction

Line 57: Arguably there are more than six Babesia species that infect humans. Authors should write "…more than eight species are known to accidentally infect humans…" (Please consult: https://doi.org/10.1017/erm.2025.10016). Maybe you want to list the most important Babesia species related to human babesiosis.

We thank the reviewer for noting this and mentioning the reference, which we included in the manuscript. We changed the text from: Among the six *Babesia* species pathogenic to humans, *B. microti* (in the United States) and *B. divergens* (in Europe) are the most prevalent, causing babesiosis[3–6]. To: "At least nine *Babesia* species (*B. divergens, B. duncani, B. crassa*-like, *B. microti, B. venatorum*, *B. odocoilei, B. bovis, B. hegotelforum,* and *B. bigemina)* are known to be pathogenic to humans, who are considered accidental hosts. *Babesia microti* (in the United States) and *B. divergens* (in Europe) are the most prevalent species that cause human babesiosis[3–7]." (now lines 57-61)

In the Introduction, the following is missing: why do they study Babesia divergens and not Babesia microti? What is their justification for the parasite choice? Please provide an estimate of how many cases of B. divergens are currently observed per year and what the typical course of the disease and prognosis are. Please compare these figures and the course of the disease with those of B. microti.

We thank the reviewer for bringing this to our attention. At the time we initiated this study, *B. microti* could not be easily cultivated (limited passage time, lower achievable parasitemia, etc.). Although *in vitro* culture methods for *B. microti* have recently been published, it would still be extremely difficult to obtain sufficient material for high-resolution studies.

Because this justification is primarily technical rather than based on relative clinical importance or incidence, we included this justification at the beginning of the Results section. The text was modified from: "We isolated *Babesia* ribosomes from an asynchronously grown *Babesia divergens* Rouen 1987 strain."

To: "Among the *Babesia sp.* pathogenic to humans, *B. microti* and *B. divergens* are the most prevalent. The ease and readily available continuous *in vitro* culture propagation of *B. divergens*, compared to *B. microti*, made it the ideal organism for ribosome structure determination. We isolated ribosomes from an asynchronously grown *B. divergens* Rouen 1987 strain." (now lines 106-109)

Line 68: What is meant by "coinfection risk"? Do you refer to the risk of humans to be accidentally infected? Please consider that humans cannot be considered as part of the life cycle of the parasite since they are a dead-end host.

We apologize for the lack of clarity. We meant coinfection with other diseases, such as HIV, rather than inclusions of humans in the parasite's natural life cycle. To avoid confusion, we removed this part of the sentence. The sentence was changed from: "This need is further underscored by the expanding geographic distribution of babesiosis and its coinfection risks in northern regions, particularly in the mid-Atlantic region of the USA."

To: "This need is further underscored by the expanding geographic distribution of babesiosis in northern regions, particularly in the mid-Atlantic region of the USA." (now lines 69-71)

Line 70 Instead of saying that the life cycle is complex, I would think it is preferable to outline in very short the life cycle of Babesia (which is not too complex), which is the parasite that you study and that is lesser known.

The reviewer is absolutely right. We now include key features of the *Babesia* life cycle. Thus, we modified the text from: "Apicomplexan parasites, such as *Plasmodium*, *Toxoplasma*, and *Babesia*, have complex life cycles. Their survival depends on the precise control of gene expression, which is primarily regulated by epigenetic, transcriptional, and post-transcriptional mechanisms."

To: "Apicomplexans, including *Plasmodium*, *Toxoplasma*, and *Babesia*, are specialized parasitic protists that utilize an apical complex to invade host cells. Their life cycle typically involves alternating between sexual reproduction in a vector and asexual reproduction within a host. Notably, *Babesia* distinguishes itself by its ability to survive tick molting (Ixodidae) and to pass from mother to offspring (transovarial transmission), allowing it to

persist without a vertebrate host and, therefore, establish reservoirs 17–19. These survival strategies are maintained through gene regulation at the epigenetic, transcriptional, and post-transcriptional levels." (now lines 72-77)

Line: 73 Toxoplasma is not transmitted by an arthopod vector. Plasmodium by mosquitos and all Babesia without exception are transmitted by ticks which is a characteristic hallmark of this group of protozoan parasites and is therefore worth mentioning.

This section of the text has been revised as described in the previous answer (lines 72–81), where we now decouple transmission modes and focus on the shared apicomplexan features while explicitly mentioning tick transmission for *Babesia*.

Line 74 Although it's not direct subject of your study: do you know how important is transcription regulation as compared to translational regulation in Babesia or Plasmodium parasites? It would be good to give a reference that compares transcriptional vs translational regulation through the life cycle (at different parasite stages).

To answer the reviewer's direct question: This is complicated, but to our knowledge, there are better examples of this in *Plasmodium.* It has recently become apparent that malaria parasites have limited transcriptional control ability (e.g., fewer transcription factors are available). There is also discordance between mRNA steady-state levels and protein abundance, suggesting that parasite genome expression is controlled in part by post-transcriptional mechanisms.

Line 89 to 97: In this part, you present some of the results of your study. However, typically, towards the end of the Introduction, the study is justified, and the objectives of the study are defined.

We thank the reviewer for bringing this to our attention. The rationale was outlined in lines 83-93 and perhaps was missed: "Protozoan ribosomes present unique regulation opportunities, as they exhibit heterogeneity and plasticity in their ribosomal protein composition, recruitment of accessory proteins, and the types of ribosomal RNA (rRNA) incorporated[21]. Ribosomal RNA modifications are also of particular interest because they have been linked to altered translation landscapes with biological relevance[22–24]; for example, they differ in patterns between healthy and tumor cells[25–27], or by altering antibiotic binding in bacteria as a mechanism of antibiotic resistance[28]. Although only a few rRNA modifications have been reported in a recent cryo-EM ribosome structure from *P. falciparum*[29], no comprehensive characterization of rRNA modifications has been conducted concomitantly with ribosome structural analysis from any apicomplexans[29–32], limiting our understanding of the mechanisms and potential roles of individual modified nucleotides in these parasites."

Line 90: Authors generalize their results to all Babesia. This is very problematic since the genetic difference (genetic distance) between Babesia species of different phylogenetic lineages is considerable and usually largely underrated. With regard to different phylogenetic lineages of Babesia you could consult (https://doi.org/10.1016/j.pt.2019.01.007 and https://doi.org/10.1007/s00436-022-07424-8). In particular, Babesia microti belonging to the phylogenetic lineage of Clade I is on a molecular level very different to B. divergens, which belongs to the phylogenetic lineage Clade VI. To give authors an idea, even highly conserved proteins such as orthologous cysteine proteinases are difficult to align to subsequently construct a tree that gives a supporting bootstrap value between sequences of B. microti and B. divergens, which belongs to Clade VI (see Fig. 1 in https://doi.org/10.3390/vetsci5020041). Therefore, it is better to specifically refer to the parasite species that you study and raise the question how different would be its ribosome architecture to that of B. microti a Babesia species distantly related to Babesia divergens. In contrast, I would expect high similarities between ribosome structures of Babesia spp. that belong to Clade VI). Therefore, the results shown in this work are related to Babesia divergens, the Authors can't generalize to the entire genus; therefore, please, write Babesia divergens instead of Babesia. Do the same throughout the manuscript (lines 40, 132, 133, 143, 144, 185, 234, etc).

We thank the reviewer for emphasizing this important point, and we apologize if our writing gave the impression that we were generalizing to all *Babesia sp*. We indeed note, for example, that eL28 is absent in *B. divergens* but present in the genomes of *B. duncani* and *B. microti*, underscoring species-species differences even within the genus.

We intended to simplify the narrative and to use *B. divergens* as a representative contrast apicomplexan ribosome with those of humans, rather than to describe all *Babesia species*.

We thus modified the manuscript to precise B. divergens each time.

Results

Line 213: higher-eukaryote (vertebrate) specific segments (ES30L and ES44L) are absent from Babesia divergens. Is the same true for Toxoplasma sp. and Plasmodium sp.?

Yes, these ES (ES30L and ES44L) are also absent in *P. falciparum* and *T. gondii* (**Table 2**).

Methods

Line 373: Gentamicin is known to block splicing mRNA in eukaryotic cells, do the Authors consider whether the use of this antibiotic could cause artifactual changes in their observations?

Gentamicin is widely used as a standard antibiotic to maintain sterility in parasite cultures without compromising viability, including at the same concentration used in our study (25 µg/mL) to prevent bacterial contamination (e.g., PMID: 28117431). At this concentration, gentamicin is unlikely to affect ribosome biogenesis or assembly significantly. We have added a brief statement to this effect in the Methods (lines 391-392).

Minor comments

Line 45: EM, should be introduced. Please check if according to journal policy you should introduce also the following abbreviations the first time they are used. Please, do the same in lines 114 (CTF), 128 (LC/MS-MS), 145 (LSU), 285 (CCA), 331 (cryo-FIB).

We thank the reviewer for this careful reading. We have revised the manuscript and ensured that all abbreviations are defined on first use (e.g., EM, CTF, LC-MS/MS, CCA, cryo-FIB), in accordance with journal policy.

Line 120: From my point of view, both ribosomes' conformations have a tRNA bound to the E-site.

The reviewer is correct; both conformations contain an E-site tRNA. We meant to emphasize ribosomes containing a *single* E-site tRNA. Therefore, we changed the text from:"… all our analysis originates from ribosomes bound to the E-site tRNA, due to its higher resolution, with areas within the ribosome core reaching atomic resolution (approximate resolution of 1.7 Å, **Fig. 1c**)."

To: "…all our analysis originates from ribosomes bound to the single E-site tRNA, due to its higher resolution, with areas within the ribosome core reaching atomic resolution (approximate resolution of 1.7 Å, **Fig. 1c**). (now line 128)

We also modified from: "We assigned rRNA modifications based on protruding density that is continuous with the ribose or base in the high-resolution maps of the *Babesia* ribosome containing an E-site tRNA (Conformational state 2, 3D class 3).

To: "… of the *Babesia* ribosome containing a single E-site tRNA (Conformational state 2, 3D class 3)." (now lines 265-266)

Line 125: The correct Babesia divergens strain is 1802A or 1802 (line 152)? Please, correct where it is needed. Do the same throughout the manuscript (lines 148, 157, 161, 306, etc).

We thank the reviewer for catching this inconsistency. The correct strain designation is 1802A; we have corrected it throughout the manuscript.

Line 163: Babesia species: Please, clarify which species of Babesia.

See previous answers.

Line 317: at the beginning of the sentence, species names should be written in full: Babesia divergens.

We have made this correction in the text.

Line 372: Please, add Trademark and origin.

We have added the manufacturer information for "Thermo Fisher Scientific", as RPMI-1640 medium is a non-trademark reagent.

Reviewer #4 (Remarks to the Author)

I co-reviewed this manuscript with one of the reviewers who provided the listed reports. This is part of the Nature Communications initiative to facilitate training in peer review and to provide appropriate recognition for Early Career Researchers who co-review manuscripts.

Response to referees

The comments from **Reviewer 1** were very positive: "*Essentially all points have been taken into account in the revised version of the manuscript.*" Although most of the final points are minor, one comment concerns the PDB validation report (line 34). In this revised version of our manuscript (track changes), we address these comments thoroughly, which are summarized in this rebuttal (red). We thank the reviewer for their helpful comments on this manuscript.

The comments from **Reviewers 3 and 4** were also very positive: "*I think the manuscript has much improved*..." However, the reviewers found a problematic paragraph, for which we are grateful for their advice on how to make it more accurate, while less complicated. All other comments were minor and have been fully addressed in the revised version of the manuscript.

Reviewer #1 (Remarks to the Author)
Essentially all points have been taken into account in the revised version of the manuscript.

Some final points:

1. The average resolution has been re-estimated to be closer to 2.7 Å, which appears fine. But it might be worth looking into the pdb validation report, where per-chain average Q-scores can give an indication on the overall fit between the atomic model and the map. Fine features can also be evaluated from per residue resolution-dependent Q-scores (in Chimera, not in ChimeraX software; needs the MapQ (v2.9.7) plugin for that).

For the ribosome structure including an E-site tRNA, which is the structure from which all our analyses originate, the overall *Q*_model average is 0.599, consistent with the resolution of 2.7 Å. We thus included this additional information in the Methods section and added a reference for the *Q*-score metric (PMID: 40654171): "We also evaluated the overall fit between the atomic model and the composite map using the *Q*-score metric[98] (overall *Q*_model per chain = 0.599). (Lines 485-486).

For the ribosome bound to three tRNAs, the overall *Q*-score average is 0.521, consistent with its lower resolution (3.2Å) when compared to the ribosome structure bound to one E-tRNA. The tRNAs have a lower *Q*-score (≤ 0.3), consistent with lower local resolution and less accurate fitting of the model into the maps. The following sentence in the manuscript was thus changed from: "The model was further refined with iterative rounds of manual adjustment in Coot and real-space refinement as described above." To: "The model was further refined with iterative rounds of manual adjustment in Coot and real-space refinement as described above (overall *Q*_model per chain = 0.521)." (Line 490-191).

2. Regarding the pdb validation report, on page 126 it says: "This section was not generated. No FSC curve or half-maps provided". That means that half maps (and probably masks also) have not been provided, but this would be useful for a proper evaluation here.

We apologize for the confusion. Each multibody map (LSU, SSU body, and SSU head) has its own corresponding half-maps and mask, and were deposited with their respective EMDB entries. The associated accession codes for all entries were included in the Data availability section when the manuscript was initially submitted. However, we apologize for omitting the validation reports for these related entries; they have now been provided.

3. It is good to see that including hydrogen atoms during atomic model refinement indeed improving the clash score, as expected. No answer required

4. Lines 168 etc. of the answer and manuscript: "folded by Alfafold3", probably "predicted" is meant uL1 "remodeled", probably modelled is meant. This is correct related to Alphafold3 and uL1. In the manuscript, to

avoid confusion, “In combination, the remodeling of ES7L, ES10L, and ES15L results in a significant modification of the LSU surface.” was changed to: “In combination, the rearrangement of ES7L, ES10L, and ES15L results in a significant modification of the LSU surface.” (Line 260). “…compact architecture of the *B. divergens* rRNA, particularly within the LSU, results in significant remodeling of its surface.” was also changed to: …compact architecture of the *B. divergens* rRNA, particularly within the LSU, results in significant modification of its surface.” (Line 324).

5. Line 246 of the answer: 2.2 to 2.6 Å probably refers to local resolution; this aspect could be mentioned in the manuscript. We agree with the reviewer and modified the text from: “We hypothesize that if that were the case, these modifications would have a lower occurrence, which would be averaged out in the cryo-EM dataset, and therefore, would be within noise level.” to “We hypothesize that if that were the case, these modifications would have a lower occurrence, which would be averaged out in the cryo-EM dataset despite being present within high local resolution (e.g., 2.2 to 2.6 Å), and therefore, would be within the noise level.” (now line 276-277).

6. Lines >267: I appreciate the answer, but (original question) could there be any false positives by nanopore sequencing? Are there any RiboMethSeq data etc. available from other parasite rRNA?

The most comprehensive rRNA modification analysis in parasites has been conducted by the group of Shulamit Michaeli, which works on *Trypanosoma brucei* and *Leishmania species*. These are completely different parasites compared to apicomplexans. To our knowledge, there are no RiboMethSeq for any apicomplexan parasites.

7. “cryo-electron microscopy (cryo-EM)” should be without hyphen when written out (hyphen only for the abbreviation) to avoid the misleading impression of electrons being “cold”, hence: “cryo electron microscopy” or “cryo-EM” to be checked throughout. We thank the reviewer for bringing this to our attention. We fully spelled cryogenic electron microscopy and abbreviated it to cryo-EM accordingly.

8. Reference 94 will be published soon, possibly update the reference later on. We take note of this, and we will update the reference upon publication.

9. Fig. S1: “Ab initio 3D model” should better say “Ab initio 3D reconstruction” (with ab initio in italics). We thank the reviewer for catching this mistake. We changed it to …3D reconstruction in Fig. S1, and italicized ab initio, both in Fig. S1 and in the manuscript (Line 439).

10. Fig. S2 legend: Fourier (not fourier), check throughout. We thank the reviewer for bringing this to our attention. We checked the manuscript and all the figure legends and made the changes accordingly.

11. Fig. S9: pink refers to uS3, not RACK1. This seems to be a β-addition motif interaction (maybe for the main text).

We thank the reviewer for bringing this to our attention. This has been changed in the figure legend.

Thank you for all other answers and changes in the manuscript, I have no other comments.

<u>Reviewer #3/4 (Remarks to the Author)</u>

General comments

I think the manuscript has much improved however there is the following very problematic paragraph that needs to be changed:

“Apicomplexans, including Plasmodium, Toxoplasma, and Babesia, are specialized parasitic protists that utilize an apical complex to invade host cells. Their life cycle typically involves alternating between sexual reproduction in a vector and asexual reproduction within a host. Notably, Babesia distinguishes itself by its ability to survive tick molting (Ixodidae) and to pass from mother to offspring (transovarial transmission), allowing it to persist without a vertebrate host and therefore, establish reservoirs 18–20.”

Please note that there are multiple errors and imprecisions in this part which have to be changed for the following reasons:

-First, protists include all unicellular eukaryotes while protozoa only the unicellular eukaryotse that are heterotrophs. Since protists are an extremely diverse and large group, the more specific and therefore preferred term is protozoan. We thank the reviewer for being specific. We thus changed "…protists…" to "…protozoan…" (line 71).

-Additionaly, Toxoplasma belongs with Eimeria and Sarcocystis to the group of coccidia, while Plasmodium and Babesia belong to hemoprotozoan. It is therefore preferable to reference this group as Toxoplasma, Plasmodium, and Babesia (that is Plasmodium neighboring Babesia). We thank the reviewer for bringing this to our attention. We changed the order of the parasites accordingly.

-Furthermore, in the case of Toxoplasma there is no arthropod vector (such as a tick in Babesia and a mosquito in Plasmodium), but only a number of intermediate vertebrate hosts (vertebrate hosts are not considered vectors). Thus, for these three groups of apicomplexa, the definitive host can be a definitive vertebrate host in the case of Toxoplasma or a definitive arthropod vector host in the case of Plasmodium and Babesia, while for all three genera the intermediate host is always a vertebrate host.) We thank the reviewer for their clarification and have changed the sentence to the one mentioned at line 146 of this rebuttal.

-The only hemoprotozoans that show transstadial and transovarial transmission is a subgroup of Babesia referred to as Babesia sensu stricto (e.g. B. divergens, B. crassa-like, B. venatorum, B. odocoilei, B. bovis, B. hegotelforum, and B. bigemina). Importantly, and as you mention, this characteristic allows that the arthropod-(tick) vector to function as vector and also as parasite reservoir. However, there is also the subgroup of Babesia sensu largo (e.g. B. duncani and B. microti) that shows only transstadial but no transovarial transmission. Therefore, your statement is erroneous as it assigns transovarial transmission to all Babesia. Probably the best solution is to delete this piece of information since it is of no relevance in the context of your study (transstadial transmission and transovarial transmission are relevant in an epidemiological context). We agree with the reviewer that this sentence is misleading. However, we rather chose to correct it, because it is the link to the "survival strategies" sentence below that allows to bring into context the gene expression regulation.

Therefore, the paragraph was changed from: "Apicomplexans, including Plasmodium, Toxoplasma, and Babesia, are specialized parasitic protists that utilize an apical complex to invade host cells. Their life cycle typically involves alternating between sexual reproduction in a vector and asexual reproduction within a host. Notably, Babesia distinguishes itself by its ability to survive tick molting (Ixodidae) and to pass from mother to offspring (transovarial transmission), allowing it to persist without a vertebrate host and therefore, establish reservoirs 18–20" to: "Apicomplexans, including *Toxoplasma*, *Plasmodium*, and *Babesia*, are specialized parasitic protozoans that utilize an apical complex to invade host cells. Their life cycle typically involves alternating between sexual reproduction in a definitive host and asexual reproduction within an intermediate host. Notably, *Babesia spp.* that are pathogenic to humans and that belong to the *sensu stricto* phylogenetic clade (e.g., *B. divergens*, *B. crassa-like*, *B. venatorum*, *B. odocoilei*, *B. bovis*, *B. hegotelforum*, and *B. bigemia*) distinguish themselves by their ability to survive tick molting (Ixodidae) and to pass from mother to offspring (transovarial transmission), allowing them to persist without a vertebrate host and therefore, establish reservoirs[18–20]."

For the above reasons please delete the above paragraph and include instead the following sentence which considers and integrates all the concerns mentioned above:

"The life cycles of Toxoplasma, Plasmodium, and Babesia typically involve alternating between sexual reproduction in a definitive host and asexual reproduction within an intermediate vertebrate host18–20."

Line 41: Babesia microti instead of B. microti since it's the start of the sentence. We corrected this.

Line 59: Add reference. We added a review PMID: 40908571 from 2025.

Line 74: Family names (Ixodidae!) should not be written in italic. We removed the italic.

Line 167: 1802A instead of 1802. We corrected this.

Line 174: Add in which Babesia species. We thank the reviewer for bringing this to our attention. The text was changed from: "… which is absent in humans but present in *P. falciparum*, *T. gondii*, and other *Babesia* species (**Fig. S6b**)." to "…which is absent in humans but present in *P. falciparum*, *T. gondii*, and *B. divergens* (**Fig. S6b**)."

Lines 182: Babesia divergens instead of Babesia. The same in Lines 213, 259, 321. We specify along the text that the structure is from *B. divergens*; abstract, introduction, etc. We disagree with the reviewer's suggestion to change it within the section titles, as it would look repetitive and make the text heavier. Therefore, this remained unchanged.

Line 297: amino group? Instead of imino. An amino group corresponds to a single bond between the carbon and the NH2 group (*sp^3*), while an imino group has a double bond between the carbon and the NH1 (*sp^2*). Pseudouridine contains an additional imino group, which can participate in a hydrogen bond with a water molecule.

Line 395, 399 and 493: five minutes or 5 minutes or 15 min? consistency throughout the manuscript. The same with seconds/sec, check please. We thank the reviewer for seeing this. The nomenclature used is always minute(s) or second(s). Numbers below 10 are fully written, while 10 and above are numbered. There were some discrepancies, which were fixed.

Line 402: tablet (Roche) instead of tablet from Roche). This was changed in the text.

Thank you for the opportunity to review this manuscript!
